# The burden of atrial fibrillation/atrial flutter in Europe from 1990 to 2021, with a forecast of incidence through 2044

**DOI:** 10.3389/fcvm.2025.1606024

**Published:** 2025-06-18

**Authors:** Min Xie, Xia Zhao, Bin He, Chunyan Lian, Jin Zhao, Xiaobo Li, Qi Lin

**Affiliations:** ^1^ Department of Cardiology, Chengdu Seventh People’s Hospital, Chengdu, China; ^2^ Department of Respiratory, Chengdu Seventh People’s Hospital, Chengdu, China

**Keywords:** atrial fibrillation/flutter, Europe, incidence, deaths, disability-adjusted life years, global health

## Abstract

**Background:**

The objective of this analysis was to assess the impact of atrial fibrillation (AF)/atrial flutter (AFL) across various European regions and countries from 1990 to 2021.

**Methods:**

Using the global burden disease 2021 analytical tools, this study evaluated the incidence, prevalence, disability-adjusted life years (DALYs) and death associated with AF/AFL across the European Region, as defined by the World Health Organization, which includes 53 member countries (EU-53), as well as the European Union, as defined in 2019, consisting of 28 member countries (EU-28) from 1990 to 2021.

**Results:**

The EU-53, in 2021, there were 957,812 incident cases [95% uncertainty interval (UI): 773,898 to 1,178,186], 103,043 deaths (95% UI: 86,887 to 111,924), and 2,196,895 DALYs (95% UI: 1,847,967 to 2 596 530) attributed to AF/AFL. The age-standardized rates (ASRs) of incidence, prevalence, death, and DALYs were respectively 1.16, 1.14, 1.06, and 1.10 times higher in the EU-28 compared to the EU-53. The absolute number of AF/AFL incidents is projected to increase from 2021 to 2044 across Western Europe (from 600,735 to 723,218), Eastern Europe (from 176,794 to 190,803), and Central Europe (from 122,625 to 135,877).

**Conclusion:**

Despite substantial efforts to manage AF/AFL in Europe, it remains a significant public health challenge. The burden of AF/AFL varies considerably across European countries and subregions, as well as between different EU classifications (EU-28 vs. EU-53). In Western Europe and the EU-28, which include many developed nations, higher ASRs of deaths, DALYs, prevalence, and incidence have been reported.

## 1 Introduction

Atrial fibrillation (AF)/atrial flutter (AFL) is the most common persistent arrhythmia, with both its incidence and prevalence increasing worldwide worldwide (1, 2). The growing burden is driven by multiple factors, including an aging population, a surge in obesity rates, enhanced detection methods, and improved survival rates among individuals with AF/AFL and other cardiovascular diseases (2–4). The worldwide prevalence of AF/AFL has seen a two-fold increase between 1990 and 2019, with the total number of cases rising to 59.7 million as of 2019 (5). This upward trend is expected to continue (6). Even more alarming, AF can lead to numerous complications, including stroke, depression, and heart failure (1, 7). These complications contribute to rising healthcare costs, greater morbidity, and higher mortality rates (8). Emerging treatments are expected to place a greater economic burden due to the expanding and aging patient, with estimates indicating that AF/AFL accounts for approximately 1% of the UK National Health Service's expenditure (9). Therefore, AF/AFL are expected to pose significant public health challenges and increase the financial costs to society in the future.

However, few recent epidemiological studies had examined the burden of AF/AFL across Europe, including both the European Union (EU-28, comprising the 27 member countries plus the United Kingdom as defined in 2019) and the broader European Region as defined by the World Health Organization (EU-53), which includes 53 member countries. Moreover, no study has offered a detailed analysis of factors such as incidence, prevalence, disability-adjusted life years (DALYs), death, and key risk factors for AF/AFL in Europe over time by year, sex and region. This study aims to provide a comprehensive and comparable assessment of the burden of atrial fibrillation and atrial flutter (AF/AFL) across Europe, using the most recent estimates from the Global Burden of Disease (GBD) 2021 study. Our analysis covers the period from 1990 to 2021, includes regional and country-level trends, and presents projections of incidence through 2044.

## 2 Methods

### 2.1 Data sources

All the data and materials are freely accessible on the GBD website, which can be found at https://vizhub.healthdata.org/gbd-results/. The methods and details used in the GBD 2021 study have been published in earlier reports (10, 11). The University of Washington's Institutional Review Board approved a waiver of informed consent for this study because it used de-identified, aggregated data. The methodology is grounded in an extensive network of international collaborators, comprising over 9,000 researchers from more than 160 countries (12). The majority of the raw data originate from external partner organizations and are obtained through various approaches, such as national censuses, structured interviews, published research, medical records and billing data, as well as biometric measurements (12). The frequency of data updates varies depending on the original source. Once collected, the data are curated and analyzed by the Institute for Health Metrics and Evaluation (IHME).

### 2.2 Study design

The International Classification of Diseases (ICD-9 and ICD-10) was used to identify cases of AF/AFL. Cardiovascular diseases coded as 427.3–427.32 in the ICD-9 and I48–I48.92 in the ICD-10 were classified as AF/AFL in this study. Consistent with earlier GBD research, AF/AFL diagnoses were based on electrocardiograms (11, 13).

This study examined the burden of AF/AFL using GBD data across all countries in the EU-53 (as defined by the World Health Organization's definition of Europe), its three subregions (Central, Eastern, and Western Europe), the EU-28 (comprising the countries in the European Union as of 2019), and all 53 individual nations within the EU-53 for both 1990 and 2021 (Supplementary Table S1). Epidemiological estimates for incidence, prevalence, death, and DALYs related to AF/AFL are provided from 1990 to 2021, broken down by sex. The analysis also includes age-standardized rates (ASRs) and numbers for both populations under 70 years old and those aged 70 and above. Additionally, we predict incidence numbers and age-standardized rates from 2022 to 2044 by country and sex.

In GBD 2021, six modifiable risk factors—namely increased systolic blood pressure, elevated body mass index (BMI), tobacco use, high-sodium diets, alcohol consumption, and lead exposure—were found to be associated with mortality from AF/AFL. These risk factors were selected based on three criteria: established causal links with AF/AFL, the availability of reliable exposure data, and their potential for intervention (13). We confirm that the attribution of risk factor contributions (e.g., high BMI, smoking) in our study was based on the comparative risk assessment framework developed by the GBD study (14).

### 2.3 Statistical analysis

We calculated the number of cases, ASRs per 100,000 individuals, and 95% uncertainty intervals (UI) to assess key GBD 2021 metrics for the AF/AFL burden, including prevalence, incidence, DALYs, and death. These metrics were analyzed by age, sex, and geographical location. The ASRs were derived through direct standardization to the global age distribution. The GBD database employed Monte Carlo simulation methods to quantify uncertainty across various model parameters (15). For each estimate, 1,000 draws were generated from the relevant probability distributions, and the 2.5th and 97.5th percentiles were used to define the lower and upper bounds of the 95% uncertainty interval. UIs were determined using 1,000 draw-level estimates for each parameter, with the 95% UI defined as the range between the 25th and 975th values among these draws. Additionally, we calculated the estimated annual percentage change (EAPC) to assess the global burden of AF/AFL. To project the number of new cases and age-standardized incidence rates from 2022 to 2044, stratified by country and sex, we applied a log-linear age-period-cohort (APC) model using the Nordpred package in R, has proven effective in accurately forecasting future incidence trends (16). The model assumes that the logarithm of the incidence rate is a linear function of age, period, and cohort effects. The model incorporates a drift term to capture long-term linear trends and uses a power-5 attenuation function to reduce the influence of more distant future projections. To ensure model adequacy, we assessed residuals and deviance statistics and compared observed vs. fitted values. Statistical significance was determined by a 95% UI that excludes zero. All statistical analyses were performed using R software, version 4.3.4.

## 3 Results

### 3.1 Incidence

#### 3.1.1 EU-53

**Figure 1:**
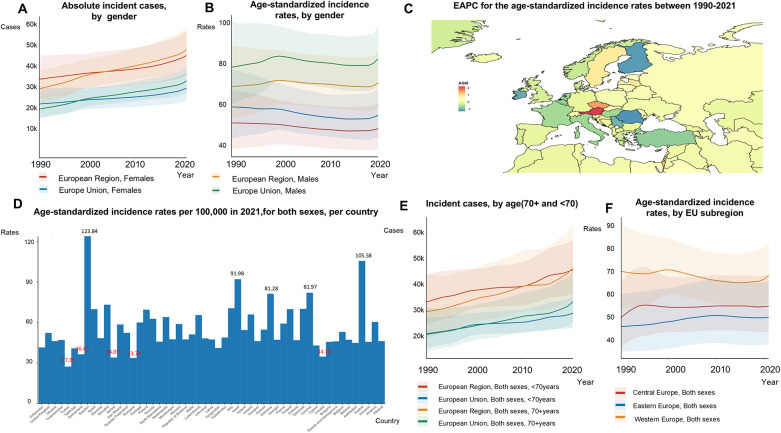
Trends in atrial fibrillation/flutter incidence rates in the EU-53 and EU-28 from 1990 to 2021. **(A)** Trend diagram showing the absolute number of incident atrial fibrillation/flutter cases from 1990 to 2021 in the EU-53 and EU-28, categorized by gender. **(B)** Trend diagram displaying the age-standardized incidence rates from 1990 to 2021 in the EU-53 and EU-28, also categorized by gender. **(C)** Heatmap illustrating the age-standardized annual percentage change in atrial fibrillation/flutter incidence rates between 1990 and 2021, per 100,000 people, across both sexes. **(D)** Age-standardized incidence rates in 2021 for all countries within the EU-53, per 100,000 people, across both sexes. **(E)** Trend diagram of incident atrial fibrillation/flutter cases in populations aged 70+ and under 70 from 1990 to 2021 in the EU-53 and EU-28, across both sexes. **(F)** Trend diagram showing the age-standardized incidence rates from 1990 to 2021, categorized by EU-53 subregion. EAPC, estimated annual percentage change; K, thousand.

The EU-53 experienced a 48% increase in the overall number of new AF/AFL cases from 1990 to 2021, reaching a total of 957,812 incident cases in 2021 (Figures 1A,E; Supplementary Table S3). However, the ASRs of incidence have remained slightly decreasing from 59.47 to 58.93 per 100,000 individuals (Figure 1B). Although men had higher ASRs of incidence of AF/AFL (70.10 in men compared to 48.75 in women in 2021), the total number of incident cases was comparable between the sexes, with 464,566 cases in women and 493,246 cases in men (Figures 1A,B). The most substantial rise in EAPC for age-standardized incidence rates was noted in Austria (2.18), the Czech Republic (1.50), Israel (1.20), and Croatia (0.86) (Figure 1C). Among the EU-53 countries, Sweden reported the highest age-standardized incidence rate, reaching 124.84 per 100,000, which is 4.56 times higher than that of Turkey in the 2021 (Figure 1D). We found that people aged less than 70 years had higher EAPC than those aged 70 years and above in both EU-53 (0.84 vs. −0.41) and EU-28 (0.95 vs. −0.57). There was also a 10% decrease in age-standardized incidence rates among those hose aged 70 years and above, dropping from 482.68 to 433.47 per 100,000, while rates among those under 70 increased by 35%, rising from 43.18 to 58.36 per 100,000, between 1990 and 2021 (Supplementary Table S3). A similar trend is seen in the EU-28 countries, and is more pronounced. Age-standardized incidence rates decreased in Western Europe, they remained significantly higher compared to those in Eastern Europe and Central Europe (Figure 1F).

#### 3.1.2 EU-28

The EU-28 experienced a 49% rise in the number of new AF/AFL cases from 1990 to 2021, with a total of 624,058 cases in 2021 (Figure 1A; Supplementary Table S2). Between 1990 and 2021, age-standardized incidence rates for AF/AFL remained largely stable, changing slightly from 68.83 to 68.50 per 100,000. In 2021, these rates were 1.16 times higher in the EU-28 compared to the EU-53 (Figure 1C). Within the EU-28, ASRs of incidence for women and men decreased by 7% and 12%, respectively.

### 3.2 Prevalence

#### 3.2.1 EU-53

**Figure 2:**
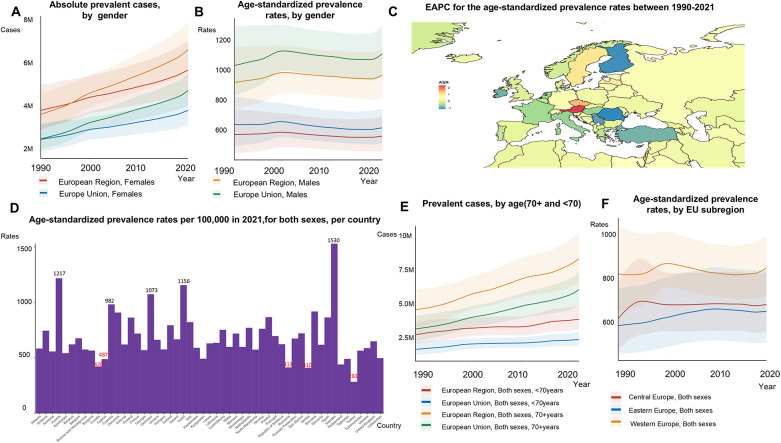
Trends in atrial fibrillation/flutter prevalence rates and numbers in the EU-53 and EU-28 from 1990 to 2021. **(A)** Trend diagram depicting the absolute number of prevalent atrial fibrillation/flutter cases between 1990 and 2021 in the EU-53 and EU-28, by gender. **(B)** Trend diagram of age-standardized prevalence rates from 1990 to 2021 in the EU-53 and EU-28, by gender. **(C)** Heatmap of the age-standardized annual percentage change in atrial fibrillation/flutter prevalence rates between 1990 and 2021, per 100,000 people, across both sexes. **(D)** Age-standardized prevalence rates in 2021 for all countries in the EU-53, per 100,000 people, across both sexes. **(E)** Trend diagram showing the prevalence of atrial fibrillation/flutter cases in populations aged 70+ and under 70 from 1990 to 2021 in the EU-53 and EU-28, across both sexes. **(F)** Trend diagram of age-standardized prevalence rates between 1990 and 2021, categorized by EU-53 subregion. EAPC, estimated annual percentage change; M, million.

**Figure 3:**
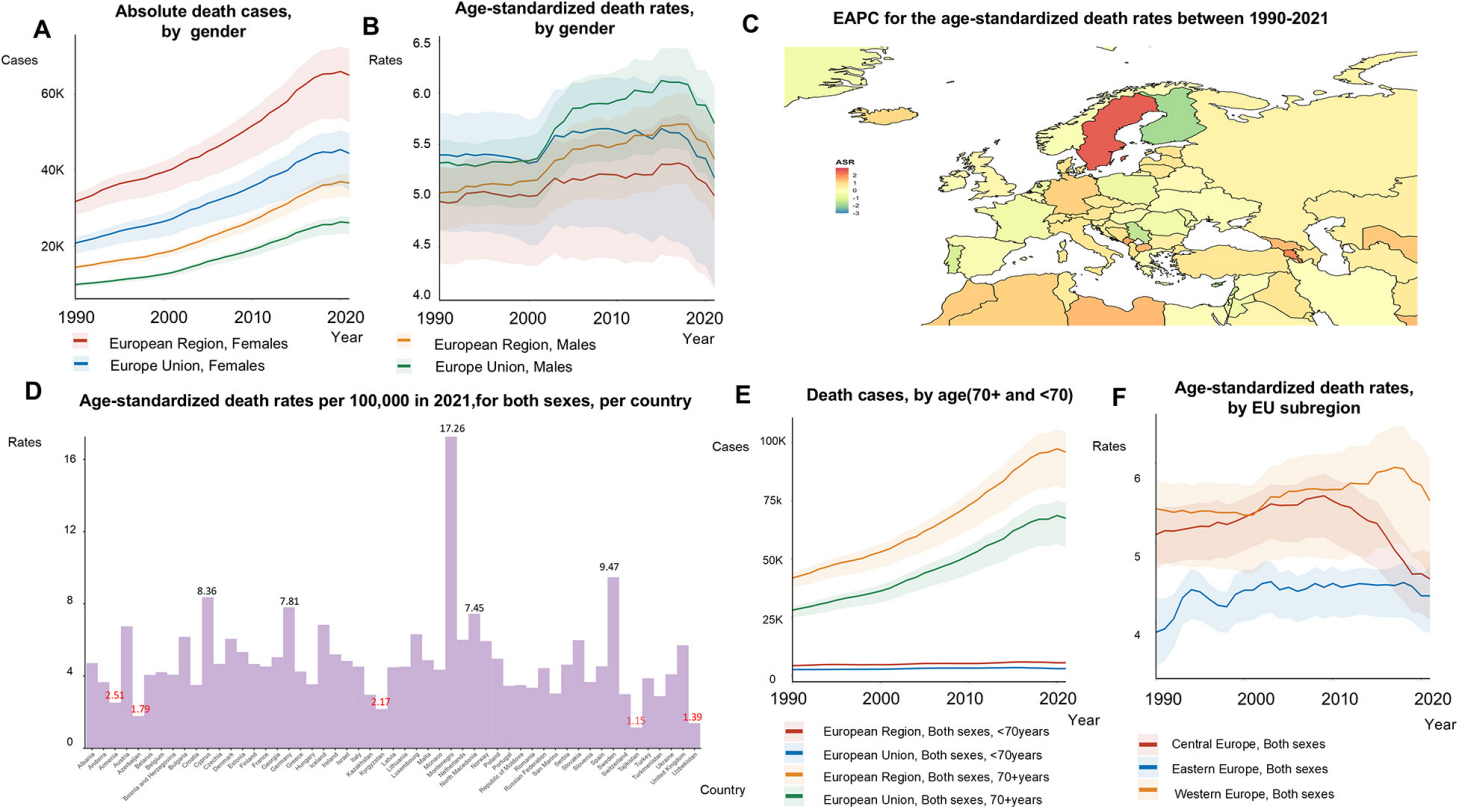
Trends in atrial fibrillation/flutter death rates and numbers in the EU-53 and EU-28 from 1990 to 2021. **(A)** Trend diagram illustrating the absolute number of atrial fibrillation/flutter-related deaths between 1990 and 2021 in the EU-53 and EU-28, by gender. **(B)** Trend diagram showing age-standardized mortality rates from 1990 to 2021 in the EU-53 and EU-28, by gender. **(C)** Heatmap of the age-standardized annual percentage change in atrial fibrillation/flutter mortality rates between 1990 and 2021, per 100,000 people, across both sexes. **(D)** Age-standardized mortality rates in 2021 for all countries within the EU-53, per 100,000 people, across both sexes. **(E)** Trend diagram of atrial fibrillation/flutter-related deaths cases in populations aged 70+ and under 70 from 1990 to 2021 in the EU-53 and EU-28, across both sexes. **(F)** Trend diagram of age-standardized mortality rates from 1990 to 2021, categorized by EU-53 subregion. EAPC, estimated annual percentage change; K, thousand.

The total number of prevalent AF/AFL cases increased by 67% from 1990 to 2021, reaching 12,922,170 cases in 2021 (Figures 2A, 3E; Supplementary Table S3). However, age-standardized prevalence rates rose by only 4% during this period, from 713.03 to 741.43 per 100,000 people (Figure 2B). Although men had higher ASRs of incidence of AF/AFL (70.10 in men compared to 48.75 in women in 2021), the total number of incident cases was comparable between the sexes, with 464,566 cases in women and 493,246 cases in men (Figures 2A, 2B). In the EU-53, the most notable reductions in the EAPC of age-standardized prevalence rates between 1990 and 2021 were observed in Finland (−1.23), Romania (−1.17), and Serbia (−1.03) (Figure 2C). Conversely, the countries with the fastest growth in the EAPC of age-standardized prevalence rates were Austria (2.33), Israel (1.46), and the Czech Republic (1.44). In 2021, Turkey had the lowest age-standardized prevalence rate, with 282.89 cases per 100,000 people. In contrast, Austria, Germany, Israel, and Sweden reported the highest age-standardized prevalence rates, each exceeding 1,000 cases per 100,000 people (Figure 2D). We found that individuals under the age of 70 exhibited a higher EAPC compared to those aged 70 and above in both the EU-53 (0.89 vs. 0.04) and EU-28 (1.00 vs. 0.05) regions. For those under 70, the rates increased by 38%, from 368.23 to 507.46 per 100,000 (Supplementary Table S3) in the EU-53 from 1990 to 2021. Age-standardized prevalence rates decreased in Western Europe, they remained significantly higher compared to Eastern Europe and Central Europe (Figure 2F), which was 1.30 times higher than that of Eastern Europe.

#### 3.2.2 EU-28

The total number of prevalent cases in the EU-28 rose by 24% from 1990 to 2021, reaching 6,209,999 in 2021 (Figure 2A). During the same period, age-standardized prevalence rates increased by 4%, from 813.56 to 848.59 per 100,000 (Figure 2B). Although both the EU-53 and EU-28 saw increases in age-standardized prevalence rates, the rate in the EU-28 was 1.14 times higher than in the EU-53. Supplementary Table S7 details the age-standardized incidence and prevalence rates for both sexes all the countries in the EU-53.

### 3.3 Deaths

#### 3.3.1 EU-53

From 1990 to 2021, the number of deaths attributed to AF/AFL surged by 120%, with a total of 103,043 AF/AFL-related fatalities recorded in 2021 (Figures 3A,E). The age-standardized mortality rate increased by 4% during this period, rising from 4.92 to 5.10 per 100,000 (Figure 3B). The absolute number of deaths was higher in women (65,882 compared to 37,161 in men). Between 1990 and 2021, Finland (−1.85), Serbia (−1.49), and Portugal (−1.17) experienced the most significant reductions in the EAPC of age-standardized death rates (Figure 3C). By 2021, Montenegro experienced the highest ASRs of death, with death rates reaching a staggering 15-fold increase compared to those in Tajikistan (17.26 vs. 1.15) (Figure 3D). Among individuals aged 70 and older, death rates increased by 33%, while they remained stable for those under 70 (Supplementary Table S3). The age-standardized death rates in Central Europe rose by 11% (from 3.87 to 4.33), decreased by 11% in Eastern Europe (from 5.10 to 4.55), and remained stable in Western Europe (5.43 to 5.52) between 1990 and 2021 (Figure 3F).

#### 3.3.2 EU-28

The EU-28 experienced a 130% increase in AF/AFL-related deaths, reaching a total of 70,530 in 2021. Although age-standardized death rates fluctuated between 1990 and 2021 (Figure 3B), the EU-28 consistently had higher death rates compared to the EU-53. In 2021, mortality rates for individuals aged 70 and older were higher in the EU-28 than in the EU-53 (Figure 3C).

### 3.4 Disability-adjusted life years

#### 3.4.1 EU-53

**Figure 4:**
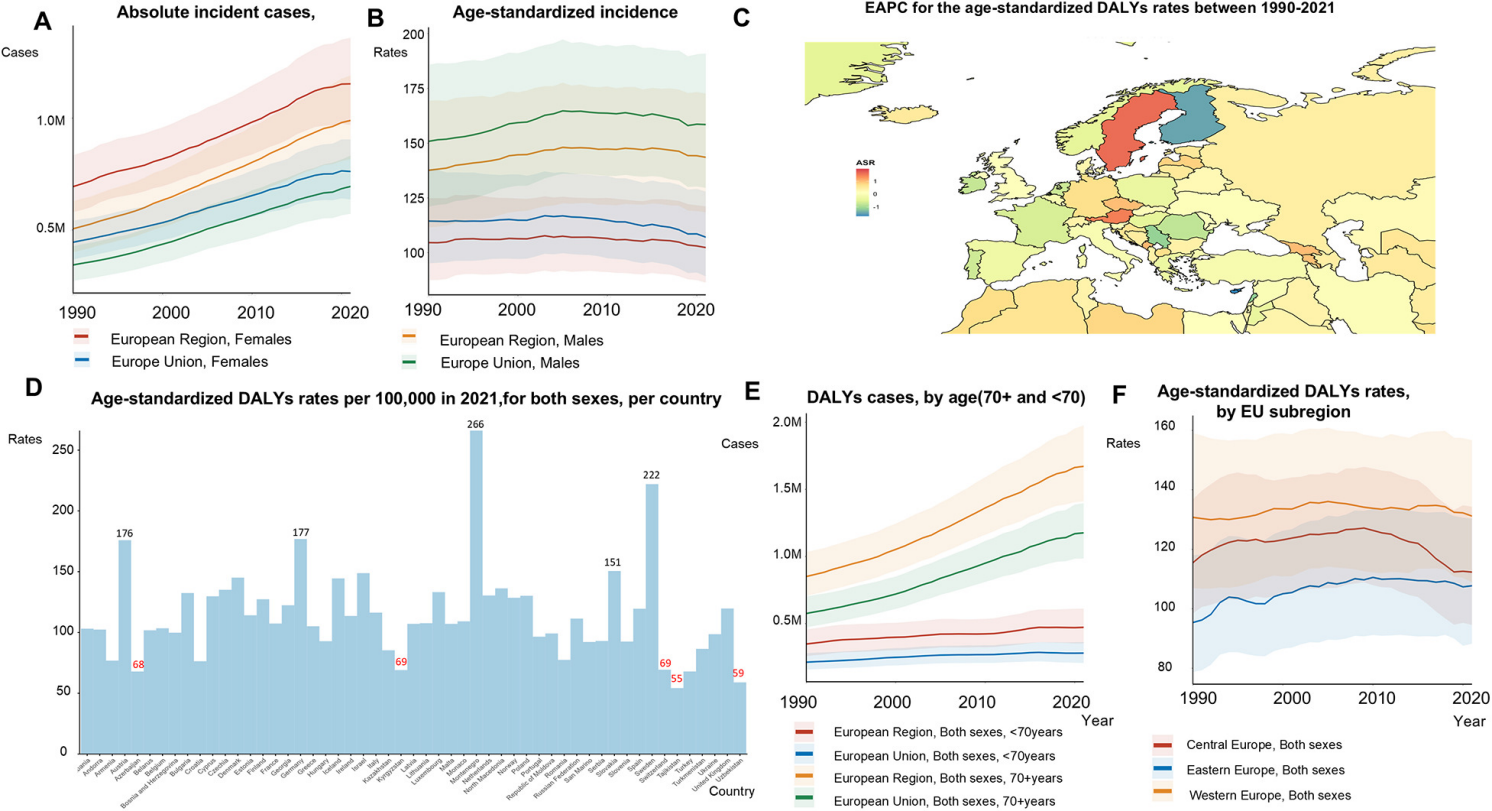
Trends in atrial fibrillation/flutter DALYs rates and numbers in the EU-53 and EU-28 from 1990 to 2021. **(A)** Trend diagram showing the absolute number of atrial fibrillation/flutter-related DALYs (disability-adjusted life years) between 1990 and 2021 in the EU-53 and EU-28, by gender. **(B)** Trend diagram of age-standardized DALYs rates between 1990 and 2021 in the EU-53 and EU-28, by gender. **(C)** Heatmap of the age-standardized annual percentage change in atrial fibrillation/flutter DALYs rates between 1990 and 2021, per 100,000 people, across both sexes. **(D)** Age-standardized DALYs rates in 2021 for all countries within the EU-53, per 100,000 people, across both sexes. **(E)** Trend diagram of atrial fibrillation/flutter-related DALYs cases in populations aged 70 + and under 70 from 1990 to 2021 in the EU-53 and EU-28, across both sexes. **(F)** Trend diagram of age-standardized DALYs rates between 1990 and 2021, categorized by EU-53 subregion. DALYs, disability-adjusted life years; EAPC, estimated annual percentage change; M, million.

The absolute number DALYs increased by 80%, from 1,223,481 in 1990 to 2,196,895 in 2021 (Figure 4A,E; Supplementary Table S3). ASRs of DALYs remained stable from 1990 to 2021 (130.74 to 131.15 per 100,000) in the EU-53. Although the absolute number of AF/AFL DALYs was higher in women, ASRs of DALYs were 0.71 times lower in women compared to men for AF/AFL (Figures 4A,B). Among the EU-53 countries, Cyprus (−1.71), and Finland (−1.41), experienced the largest decreases in the EAPC of age-standardized DALYs rates between 1990 and 2021, followed by Serbia (−1.12) (Figure 4C). In contrast, the countries with the fastest growth in the EAPC of age-standardized DALYs rates were Sweden (1.61), Austria (1.46), Montenegro (1.04), Georgia (1.00), and Czech Republic (0.99). In 2021, Tajikistan had the lowest age-standardized DALYs rates, with 54.26 per 100,000 people. In contrast, Austria, Germany, Montenegro, and Sweden reported age-standardized DALYs rates exceeding 150 per 100,000 people (Figure 4D). The absolute number of DALYs continued to rise in Central, Eastern, and Western Europe, although significant reductions in age-standardized DALYs rates were observed in Central Europe (Figure 4F).

#### 3.4.2 EU-28

The total number of DALYs attributable to AF/AFL increased, reaching 1,455,927 in 2021 (Figure 4A) in the EU-28. During this period, the ASRs of DALYs in the EU-28 remained relatively stable, changing slightly from 130.82 in 1990 to 130.70 in 2021. The ASRs of DALYs for AF/AFL were 1.10 times higher in the EU-28 compared to the EU-53. Supplementary Table S10 details the ASRs of DALYs and deaths for all the countries in the EU-53 and EU-28.

### 3.5 Risk factors and prediction of incidence

**Figure 5:**
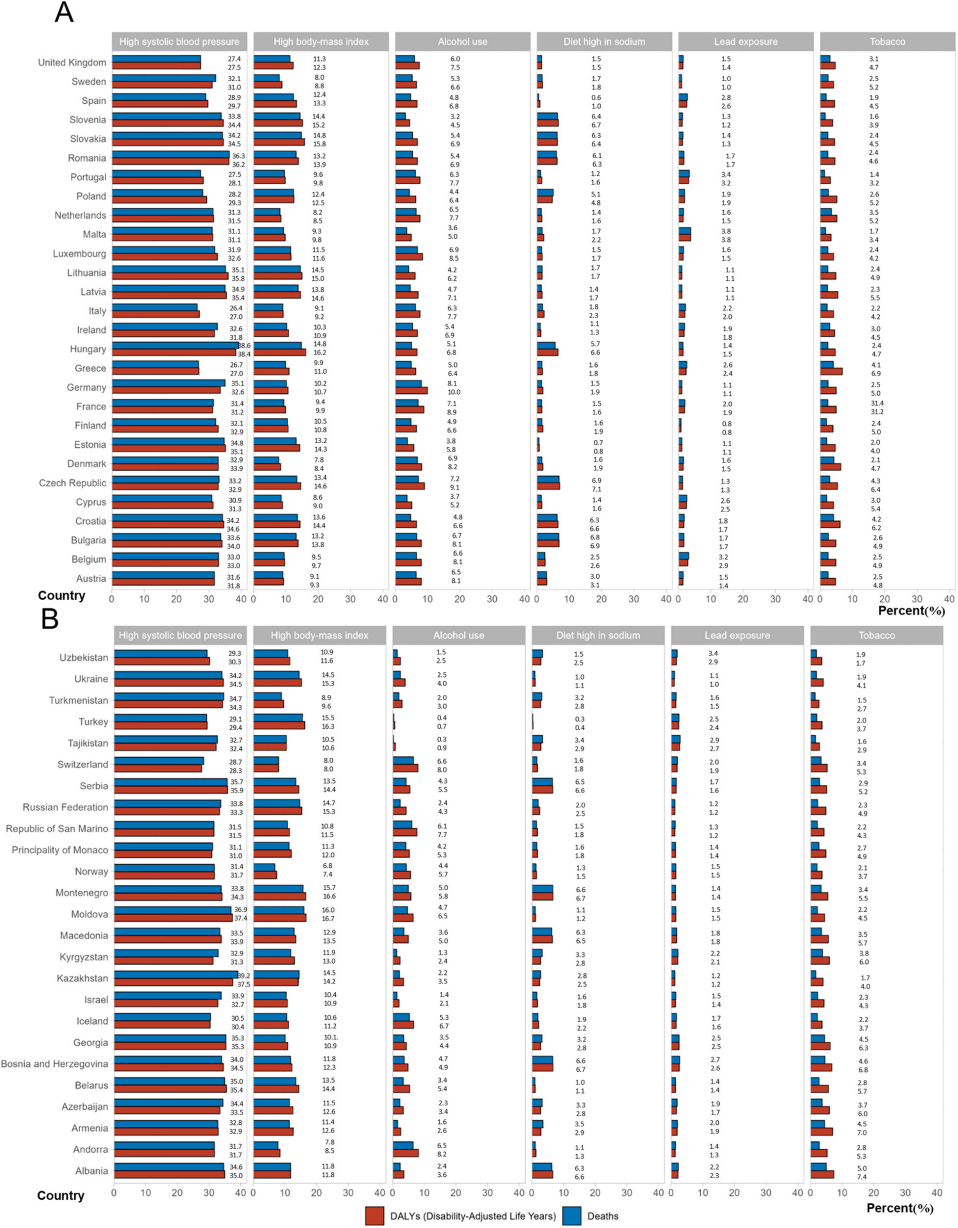
Percentage contributions of major risk factors to age-standardized death/DALYs of atrial fibrillation/flutter in 2021. **(A)** EU-28 countries, **(B)** Other countries in EU-53 except EU-28. DALYs, disability-adjusted life years.

The estimates of modifiable risk factors provided by the GBD do not account for 100% of the total AF/AFL risk. Among these risk factors, high systolic blood pressure is the most significant across all EU-53 countries, followed by high BMI (Figure 5; Supplementary Tables S11–S12). From 1990 to 2021, the percentage contribution of high BMI and lead exposure to age-standardized AF/AFL deaths and DALYs increased, while the contributions of smoking and high blood pressure declined. In 2021, high systolic blood pressure caused more than 35% of AF/AFL-related deaths/DALYs in 12 countries, including Serbia, Moldova, Kazakhstan, Georgia, Belarus, Azerbaijan, Albania, Germany, Romania, Latvia, Lithuania, and Hungary. In 8 countries, high BMI caused more than 15% of AF/AFL-related deaths/DALYs, including Turkey, Montenegro, Moldova, Hungary, Slovakia, Slovenia, Ukraine, and Russia.

**Figure 6:**
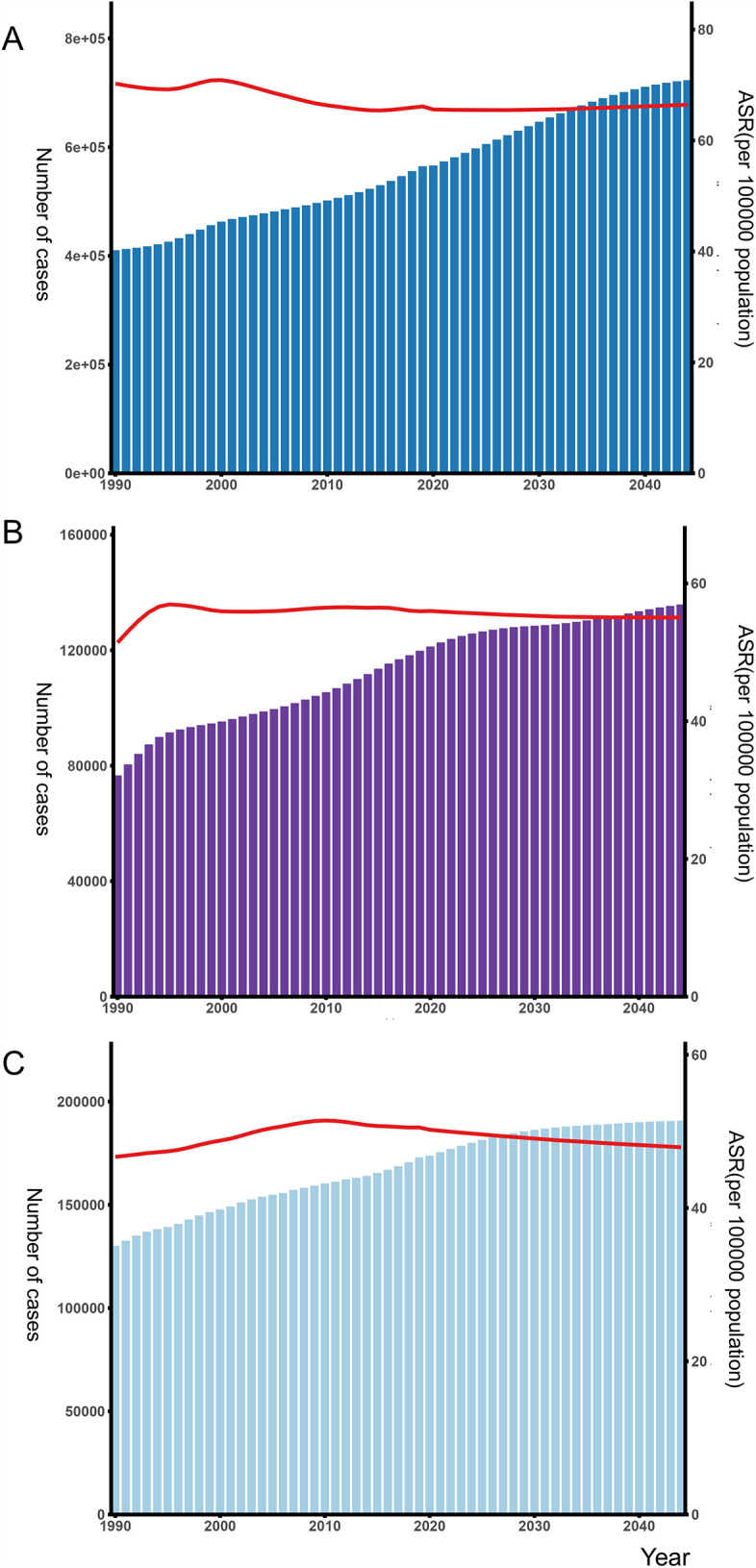
Number of atrial fibrillation/flutter and age-standardized incidence rates in the **(A)** Western Europe, **(B)** Central Europe **(C)** Eastern Europe from 1990 to 2044 by sex. ASR, age-standardized rate.

Absolute incident numbers of AF/AFL are projected to increase from 2021 to 2044 in Western Europe (from 600,735 to 723,218), Eastern Europe (from 176,794 to 190,803), and Central Europe (from 122,625 to 135,877) (Figure 6). ASRs of incidence are expected to decrease slightly from 2022 to 2044 in Western Europe (from 68.19 to 66.1), Eastern Europe (from 50.47 to 47.92), and Central Europe (from 55.16 to 55.04). Despite this decrease in rates, the total number of cases has risen in all regions. In Western and Central Europe, both overall incidence rates and the number of AF/AFL cases are consistently higher in males compared to females. However, in Eastern Europe, women have a higher overall incidence of AF/AFL than men, although their age-standardized incidence rate remains lower. In our projections from 2021 to 2044, only two countries, Serbia and Finland, show a decline in incidence numbers. The forecast data for all countries can be seen in Supplementary Figures S1–S7.

## 4 Discussion

This study reflects significant geographic variations in the burden of AF/AFL across EU-53, EU-28, Western, Central, and Eastern Europe, as well as between individual countries. In addition, males have higher age-standardized rates of AF/AFL-related incidents, deaths and DALYs than females. We also found that the incidence and prevalence of AF increased rapidly in people under 70 years of age compared with those over 70 years of age. Based on our forecasts, the number of people with AF/AFL will continue to rise over the next 20 years. Our results reveal that AF/AFL remains a major health issue, highlighting the urgent need to both identify and develop more effective treatment strategies for this condition.

The current study reveals significant geographic variability in the AF/AFL burden across Europe. Although the absolute numbers of AF/AFL incidence and prevalence have increased throughout Europe, the age-standardized rates for deaths and DALYs remain consistently higher than the global average. Earlier projections indicated that the burden of AF would escalate to endemic levels across Europe, leading to higher hospitalization rates (6). We found that age-standardized incidence rates showed an overall downward trend. This contrasts with other studies that have reported a growing incidence of AF over time (17). Age-standardized rates of incidence, prevalence, DALYs, and death related to AF/AFL are higher in the EU-28 compared to the EU-53. Additionally, Western Europe consistently shows higher cases and rates of incidence, prevalence, death, and DALYs than Eastern and Central Europe. The EU-28 and Western Europe predominantly consist of developed countries. Consistent with our findings, other studies also indicate that developed nations bear a greater overall AF burden (17, 18). In wealthier countries, there may be a “survivor effect,” where individuals live long enough to be diagnosed with AF or its severe complications (1). A previous study by Ohlrogge et al. suggested a potential link between greater AF/AFL burden and higher physician density in high-income nations, based on findings from earlier GBD analyses (12). Additionally, poorer health systems with limited access to ECGs might underdiagnose AF, leading to misclassification of death causes in these regions (19). People in low GDP European countries experience wealth inequality, with a larger proportion of the population belonging to lower socioeconomic classes and lower per capita healthcare expenditure (20). It may seem counterintuitive that these countries—primarily non-EU nations within the EU-53—exhibited lower AF/AFL-related mortality and incidence in our study. In contrast, Germany and Sweden, with per capita health expenditures significantly above the EU average, recorded some of the highest AF/AFL-related mortality and DALYs rates in 2021. We have expanded our analysis using GBD 2021 data to compare AF trends (1990–2021) across Canada, the United States, China, and the EU-53 (11). Canada showed declining EAPCs in incidence, prevalence, mortality, and DALYs, while all indicators increased in the U.S. In China, mortality and DALYs declined, but incidence and prevalence rose (11). In the EU-53, only incidence declined.

Understanding how risk factors are distributed across Europe may clarify why AF outcomes differ, particularly favoring less-developed member states. The prevalence of modifiable risk factors—such as obesity, alcohol consumption, smoking, and physical inactivity—along with related comorbidities like hypertension, coronary artery disease, and diabetes, varies significantly by region (21). For instance, alcohol consumption, and rates of dyslipidemia are generally higher in Western European countries, while these factors are lower in Eastern Europe (21). Conversely, diabetes mellitus and hypertension are more prevalent in lower-income European countries (22). This suggests that the differences in AF incidence, deaths, and DALYs are influenced by factors beyond just risk factor distribution. Overall, the disease burden of AF is higher in the highest GDP/developed countries.

Previous studies have found that women are more prone to higher morbidity and complications associated with AF/AFL (23), which may be attributed to the underuse of rhythm control strategies and a lower rate of oral anticoagulant treatment (24). A meta-analysis also highlights an increased risk of thrombosis and stroke in women (25). A study analyzing AF-related mortality in Europe over the past decade found that, despite a larger increase in age-standardized mortality rates among men, the total number of deaths was higher in women (26). However, in our study we found that males had higher age-standardized rates of AF/AFL-related incidents, deaths and DALYs than females in Europe.

The long-established Framingham Heart Study highlights aging as the most significant risk factor for AF, outweighing other contributing factors (27). Aging is associated with mitochondrial dysfunction, oxidative stress, ion channel inactivation, and cardiomyocyte hypertrophy, all of which promote atrial remodeling, thereby increasing the risk of AF (2). Additionally, we observed that the number and age-standardized rates of DALYs for AF patients under 70 are also increasing. Numerous studies indicate that younger AF patients face more severe consequences (28, 29). We speculate that the increase in younger patients with atrial fibrillation may be attributed to the following factors. First, advances in diagnostic tools and greater accessibility to electrocardiograms (ECGs), including the use of wearable and ambulatory monitoring devices, have improved the detection of asymptomatic or paroxysmal AF, particularly in younger populations (17). Second, there has been a global rise in modifiable risk factors such as obesity, hypertension, physical inactivity, and alcohol consumption, which are more prevalent among middle-aged adults and are strongly associated with AF pathogenesis (30). Additionally, increased public and clinical awareness, as well as more widespread screening in primary care settings, may contribute to earlier diagnoses. Long-term exposure to AF in younger patients leads to a notable decline in quality of life and psychological health and affects life expectancy (31). Young AF patients remain at risk for stroke, particularly those with other cardiovascular risk factors, and the association of AF with a significantly higher risk of sudden cardiac arrest is notable (32). The management of relatively young patients with atrial fibrillation is an issue that needs attention.

Based on data provided by the GBD, we projected the incidence number and ASRs for the next two decades. Overall, the number of new cases continues to rise, while the age-standardized incidence rate remains relatively stable. The AF/AFL incidence numbers and rates are expected to remain high in the coming decades. This trend is primarily driven by an aging population, lifestyle changes, and the widespread presence of cardiovascular risk factors such as obesity, hypertension, and diabetes (33, 34). Advancements in AF screening technologies and their widespread adoption have led to the detection of a significant number of asymptomatic cases (35). Most importantly, AF is often accompanied by multiple comorbidities; implementing comprehensive management strategies to address these coexisting conditions is crucial for reducing AF-related risks (36). Overall, whether in Europe or other regions, AF/AFL remains a significant global public health issue, and managing AF patients continues to be a considerable challenge.

### 4.1 Limitation

While these findings are important, several limitations should be recognized. Frist, the data collection for the GBD database depends on a broad network of international collaborators and relies on access to administrative records related to healthcare interactions and death certification. Access to this information may vary by country, potentially affecting the robustness of the data. Second, although AF/AFL were grouped together due to the data granularity in the GBD database, it is important to acknowledge that these two arrhythmias do not present the same risks. Patients with AF experience higher rates of stroke, heart failure, hospitalization, and mortality compared to those with AFL (37). Third, the GBD studies have yet to incorporate additional risk factors such as heart failure, diabetes, chronic kidney disease, and hyperthyroidism into their analyses. Fourth, we acknowledge that the APC model has limitations, such as the identifiability issue among age, period, and cohort effects, and the assumption that past trends persist without major disruptions. Nevertheless, it remains a widely accepted and robust tool for projecting disease trends. Finally, it is important to note that there are various types of AF; however, the GBD database does not account for this complexity and categorizes populations under the general AF classification.

## 5 Conclusions

This study highlights significant geographic variations in the burden of AF/AFL across EU-53, EU-28, and different regions of Europe. Notably, the incidence and prevalence of AF are increasing rapidly in individuals under 70, while the overall number of AF/AFL cases is projected to rise over the next 20 years. These findings underscore the significant public health challenge for AF/AFL.

## Data Availability

The data used in this study are publicly available from the Global Burden of Disease (GBD) database, provided by the Institute for Health Metrics and Evaluation (IHME). Researchers can access the data at the following website: http://ghdx.healthdata.org/gbd-results-tool.
