# Supplementary material for: The burden of atrial fibrillation/atrial flutter in Europe from 1990 to 2021, with a forecast of incidence through 2044
Source: Front Cardiovasc Med. 2025 Jun 18;12:1606024. doi: 10.3389/fcvm.2025.1606024 (PMC12216976; doi:10.3389/fcvm.2025.1606024)
Supplement: Supplementary file 1 [file Datasheet1.docx]

Supplemental Material

The burden of atrial fibrillation/atrial flutter in Europe from 1990 to 2021, with a forecast of incidence through 2044

Table S1. List of included countries in the EU-53 region, Western, Eastern and Central Europe subregions, and EU-28.

Table S2: Absolute and relative measures of the burden of atrial fibrillation/ flutter in the EU-28 in 1990 and 2021

Table S3: Absolute and relative measures of the burden of atrial fibrillation/ flutter in the EU-53 in 1990 and 2021

Table S4: Burden of atrial fibrillation/flutter in the Western Europe in 1990 and 2021

Table S5: Burden of atrial fibrillation/flutter in the Eastern a Europe in 1990 and 2021

Table S6: Burden of atrial fibrillation/flutter in the Central Europe in 1990 and 2021

Table S7: Atrial fibrillation/flutter in EU-53: Incidence by country, sex, and age

Table S8: Atrial fibrillation/flutter in EU-53: Prevalence by country, sex, and age

Table S9: Atrial fibrillation/flutter in EU-53: Death by Country, sex, and age

Table S10: Atrial fibrillation/flutter in EU-53: DALYs by Country, sex, and age

Table S11: Percentage contributions of major risk factors to age-standardized death/DALYs rates of atrial fibrillation/flutter in 1990(EU-28 countries). DALYs: Disability-Adjusted Life Years.

Table S12: Percentage contributions of major risk factors to age-standardized death/DALYs rates of atrial fibrillation/flutter in 1990 (other countries in EU-53 except EU-28)

Figure S1 Percentage contributions of major risk factors to age-standardized death/DALYs rates of atrial fibrillation/flutter in 1990. A: EU-28 countries, B: Other countries in EU-53 except EU-28. DALYs: Disability-Adjusted Life Years.

Figure S2 Age-standardized atrial fibrillation/flutter incidence rates in 1990, 2010, and 2021 and predicted rates in 2044, both sexes combined

Figure S1 Number of AF/AFL Cases and Age-Standardized Incidence Rates by Sex in the EU-28 (A)Austria, (B)Belgium, (C)Bulgaria, (D)Croatia, (E)Cyprus, (F)Czech Republic, (G)Denmark, (H)Estonia from 1990 to 2044

Figure S2 Number of AF/AFL Cases and Age-Standardized Incidence Rates by Sex in the EU-28 (A) Latvia, (B)Finland, (C) France, (D)Germany (E)Greece, (F) Hungary, (G) Ireland, (H) Italy from 1990 to 2044

Figure S3 Number of AF/AFL Cases and Age-Standardized Incidence Rates by Sex in the EU-28 (A) Lithuania, (B) Luxembourg, (C) Malta, (D) Netherlands (E) Poland, (F) Portugal, (G) Romania, (H) Slovakia from 1990 to 2044

Figure S4 Number of AF/AFL Cases and Age-Standardized Incidence Rates by Sex in the EU-28 (A) Slovenia, (B) Spain, (C) Sweden, (D) United Kingdom from 1990 to 2044

Figure S5 Incident number of AF/AFL cases and age-standardized incidence rates by sex for other countries in the EU-53 (not part of the EU-28) (A) Albania, (B) Andorra, (C) Armenia, (D) Azerbaijan, (E) Belarus, (F) Bosnia and Herzegovina, (G) Georgia, (H) Iceland from 1990 to 2044

Figure S6 Incident number of AF/AFL cases and age-standardized incidence rates by sex for other countries in the EU-53 (not part of the EU-28) (A) Israel, (B) Kazakhstan, (C) Kyrgyzstan, (D) Montenegro, (E) North Macedonia, (F) Norway, (G) Republic of Moldova, (H) Russian Federation from 1990 to 2044

Figure S7 Incident number of AF/AFL cases and age-standardized incidence rates by sex for other countries in the EU-53 (not part of the EU-28) (A)Serbia, (B) Switzerland, (C) Tajikistan, (D) Turkey, (E) Turkmenistan, (F) Ukraine, (G) Uzbekistan from 1990 to 2044

Table S1 List of included countries in the EU-53 region, Western, Eastern and Central Europe subregions, and EU-28.

| Region | Countries Included | N |
| --- | --- | --- |
| EU-53 | Albania, Andorra, Armenia, Austria, Azerbaijan, Belarus, Belgium, Bosnia & Herzegovina, Bulgaria, Croatia, Cyprus, Czech Republic, Denmark, Estonia, Finland, France, Georgia, Germany, Greece, Hungary, Iceland, Ireland, Israel, Italy, Kazakhstan, Kyrgyzstan, Latvia, Lithuania, Luxembourg, Malta, Monaco, Montenegro, Netherlands, North Macedonia, Norway, Poland, Portugal, Republic of Moldova, Romania, Russian Federation, San Marino, Serbia, Slovakia, Slovenia, Spain, Sweden, Switzerland, Tajikistan, Turkey, Turkmenistan, Ukraine, United Kingdom, Uzbekistan. | 53 |
| Western Europe | Andorra, Austria, Belgium, Cyprus, Denmark, Finland, France, Germany, Greece, Iceland, Ireland, Israel, Italy, Luxembourg, Malta, Monaco, Netherlands, Norway, Portugal, San Marino, Spain, Sweden, Switzerland, United Kingdom | 24 |
| Central Europe | Albania, Bosnia & Herzegovina, Bulgaria, Croatia, Czech Republic, Hungary, Montenegro, North Macedonia, Poland, Romania, Serbia, Slovakia, Slovenia. | 13 |
| Eastern Europe | Belarus, Estonia, Latvia, Lithuania, Republic of Moldova, Russian Federation, Ukraine. | 7 |
| Not included in a  subregion* | Armenia, Azerbaijan, Georgia, Kazakhstan, Kyrgyzstan, Tajikistan, Turkey, Turkmenistan, Uzbekistan. | 9 |
| EU-28 | Austria, Belgium, Bulgaria, Croatia, Cyprus, Czech Republic, Denmark, Estonia, Finland, France, Germany, Greece, Hungary, Ireland, Italy, Latvia, Lithuania, Luxembourg, Malta, Netherlands, Poland, Portugal, Romania, Slovakia, Slovenia, Spain, Sweden, United Kingdom | 28 |

Legend:

EU-53: Refers to the 53 countries in Europe as defined by the World Health Organization (WHO).

Subregions: Europe is divided by the WHO into three subregions: Western, Central, and Eastern Europe.

EU-28: Refers to the European Union as it was defined in 2019, including its 27 current member states plus the United Kingdom.

Note: Not all countries in the EU-53 are included in the WHO-defined subregions. Therefore, a separate row has been created to list the countries in the EU-53 that are not part of any subregion.

Table S2 Absolute and relative measures of the burden of atrial fibrillation/ flutter in the EU-28 in 1990 and 2021

|  | **EU28** |  |  |
| --- | --- | --- | --- |
|  | **1990 n (95% UI)** | **2021 n (95% UI)** | **1990-2021EAPC**  **(95% CI)** |
| **INCIDENCE** |  |  |  |
| Absolute Numbers | 419003(317903- 549057) | 624058(765809-508018) | 1.15 (1.10 to 1.20) |
| Age-Standardized Rates | 68.83(53.23- 88.58) | 68.50(56.79 -82.34) | -0.19 (-0.25 to -0.14) |
| Age-standardized Rates Females | 59.31(44.15-77.32) | 55.21(44.71 -68.02) | -0.38 (-0.43 to -0.34) |
| Age-standardized Rates Males | 78.21(60.73- 99.96) | 68.50(56.79 -82.34) | -0.03 (-0.1 to 0.04) |
| Rate in the population 70+ | 569.34(377.59-841.60) | 497.44(378.00- 677.10) | -0.57 (-0.65 to -0.49) |
| Rate in the population < 70 | 54.16(40.42-71.12) | 76.31(61.09 -92.55) | 0.95 (0.86 to 1.03) |
| **PREVALENCE** |  |  |  |
| Absolute Numbers | 5023176(3937752-6434732) | 6209999(4988632-7564774) | 1.69 (1.63 to 1.74) |
| Age-Standardized Rates | 813.56(643.73- 1037.45) | 848.59(717.92-1001.49) | -0.02 (-0.10 to 0.05) |
| Age-standardized Rates Females | 640.66(493.29-831.72) | 618.60(513.18-748.57) | -0.24 (-0.3 to -0.18) |
| Age-standardized Rates Males | 1040.61(831.71- 1310.62) | 1119.28(964.89-1308.75) | 0.05 (-0.04 to 0.14) |
| Rate in the population 70+ | 8832.79(6364.94-11542.91) | 9230.29(7414.90-11243.97) | 0.05 (-0.04 to 0.14) |
| Rate in the population < 70 | 455.77(346.76-600.96) | 651.20(528.01-794.08) | 1.00 (0.89 to 1.11) |
| **DEATH** |  |  |  |
| Absolute Numbers | 30616(27321-32391) | 70530(58055-77276) | 3.03 (2.94 to 3.12) |
| Age-Standardized Rates | 5.41(4.78- 5.75) | 5.40(4.50- 5.89) | 0.25 (0.16 to 0.34) |
| Age-standardized Rates Females | 5.40(4.66- 5.80) | 5.17(4.13- 5.74) | 0.09 (0.00 to 0.18) |
| Age-standardized Rates Males | 5.32(4.94- 5.54) | 5.70(5.04-6.05) | 0.52 (0.42 to 0.62) |
| Rate in the population 70+ | 75.79(67.07 -80.48) | 100.43(81.99-110.37) | 1.22 (1.10 to 1.34) |
| Rate in the population < 70 | 0.66(0.64- 0.68) | 0.78(0.75 -0.81) | 0.88 (0.69 to 1.06) |
| **DALYs** |  |  |  |
| Absolute Numbers | 788177(643142-962610) | 1455927(1225660-1727928) | 2.13 (2.08 to 2.19) |
| Age-Standardized Rates | 130.82(107.30-158.93) | 130.70(108.54-156.12) | 0.05(-0.02to 0.12) |
| Age-standardized Rates Females | 114.46(95.13- 138.15) | 107.10(89.23-127.91) | -0.15 (-0.21 to -0.08) |
| Age-standardized Rates Males | 150.82(121.01-185.78) | 158.47(129.61- 190.93) | 0.20 (0.12 to 0.28) |
| Rate in the population 70+ | 1553.42(1274.51- 1895.39) | 1747.60(1462.91-2075.48) | 0.56 (0.5 to 0.62) |
| Rate in the population < 70 | 55.37(41.08-72.69) | 73.87(54.47-95.56) | 0.94 (0.84 to 1.04) |

Given epidemiological measures include absolute numbers, age standardized rates per 100,000 population, age standardized rates for the female and

male population per 100,000 females and males, respectively, rates in the population 70 years or older per 100,000 and rates in the population under the age of 70, per 100,000. All of these have been given with their uncertainty intervals (in parenthesis).

DALYs: Disability adjusted life years; EAPC: Estimated annual percentage change. UI: Uncertainty intervals.

Table S3 Absolute and relative measures of the burden of atrial fibrillation/ flutter in the EU-53 in 1990 and 2021

|  | **EU-53** |  |  |
| --- | --- | --- | --- |
|  | **1990n(95%UI)** | **2021n (95% UI)** | **1990-2021EAPC**  **(95% CI)** |
| **INCIDENCE** |  |  |  |
| Absolute Numbers | 645410(495377- 842225) | 957812(773898-1178186) | 1.15 (1.11 to 1.19) |
| Age-Standardized Rates | 59.47(46.04-76.83) | 58.93(47.79-72.11) | -0.14 (-0.18 to -0.11) |
| Age-standardized Rates Females | 51.30(38.87-67.18) | 48.75(39.06-60.81) | -0.28 (-0.31 to -0.24) |
| Age-standardized Rates Males | 72.38(55.94-93.23) | 70.10(57.78-85.00) | -0.04 (-0.08 to 0.01) |
| Rate in the population 70+ | 482.68(316.65-701.73) | 433.47(322.29-592.30) | -0.41 (-0.48 to -0.34) |
| Rate in the population < 70 | 43.18(32.03-56.95) | 58.36(46.04-73.08) | 0.84 (0.78 to 0.9) |
| **PREVALENCE** |  |  |  |
| Absolute Numbers | 7731572(6080359-9864456) | 12922170(10770947-15682289) | 1.60 (1.56 to 1.63) |
| Age-Standardized Rates | 713.03(563.47-907.35) | 741.43 (619.28 -890.72) | 0.01 (-0.04 to 0.07) |
| Age-standardized Rates Females | 570.23(442.50-740.50) | 561.87(461.61-695.46) | -0.15 (-0.2 to -0.11) |
| Age-standardized Rates Males | 919.20(735.71-1162.48) | 968.19(822.12-1151.08) | 0.04 (-0.03 to 0.11) |
| Rate in the population 70+ | 7517.26(5462.85-9923.81) | 7940.08(6281.61-9788.35) | 0.04 (-0.03 to 0.11) |
| Rate in the population < 70 | 368.23(278.28-486.20 | 507.46(404.49-636.62) | 0.89 (0.82 to 0.97) |
| **DEATH** |  |  |  |
| Absolute Numbers | 46783(42474- 49297) | 103043(86887-111924) | 2.82 (2.73 to 2.92) |
| Age-Standardized Rates | 4.92 (4.40 -5.22) | 5.10(4.33-5.52) | 0.29 (0.23 to 0.35) |
| Age-standardized Rates Females | 4.87(4.28-5.20) | 4.95(4.06-5.44) | 0.20 (0.15 to 0.26) |
| Age-standardized Rates Males | 4.94(4.57-5.20) | 5.30(4.71-5.62) | 0.43 (0.37 to 0.5) |
| Rate in the population 70+ | 66.40(59.85-70.17) | 88.38(73.77-96.28) | 1.19 (1.03 to 1.36) |
| Rate in the population < 70 | 0.54(0.52-0.57) | 0.69(0.66- 0.72) | 0.83 (0.73 to 0.92) |
| **DALYs** |  |  |  |
| Absolute Numbers | 1223481(1007877-1499360) | 2196895(1847967-2596530) | 2.00 (1.97 to 2.03) |
| Age-Standardized Rates | 117.11(97.25-142.26) | 119.67(99.90-142.83) | 0.10 (0.05 to 0.15) |
| Age-standardized Rates Females | 103.42(86.49-123.49) | 101.65(85.74-120.45) | -0.03 (-0.07 to 0.02) |
| Age-standardized Rates Males | 135.98(110.18-167.50) | 142.22(117.25- 171.14) | 0.18 (0.12 to 0.24) |
| Rate in the population 70+ | 1354.35(1125.20- 1646.05) | 1546.08(1303.48-1828.97) | 0.59 (0.5 to 0.67) |
| Rate in the population < 70 | 45.01(33.28-59.07) | 60.03(44.81- 77.95) | 0.87 (0.81 to 0.92) |

Given epidemiological measures include absolute numbers, age standardized rates per 100,000 population, age standardized rates for the female and male population per 100,000 females and males, respectively, rates in the population 70 years or older per 100,000 and rates in the population under the age of 70, per 100,000. All of these have been given with their uncertainty intervals (in parenthesis).

DALYs: Disability adjusted life years; EAPC: Estimated annual percentage change. UI: Uncertainty intervals

Table S4 Burden of atrial fibrillation/flutter in the Western Europe in 1990 and 2021

|  | **Western Europe*** | | |
| --- | --- | --- | --- |
|  | **1990**  **n(95%UI)** | **2021**  **n (95% UI)** | **1990-2021 EAPC (95% CI)** |
| **INCIDENCE** |  |  |  |
| Absolute Numbers | 410475(310301- 539190) | 600735(491661-740671) | 1.12(1.07 to 1.17) |
| Age-Standardized Rates | 69.97(54.22-90.22) | 68.19(56.6-82.27) | -0.25 (-0.31 to -0.18) |
| Age-standardized Rates Females | 59.26(44.12-78.18) | 54.52(44.19-67.61) | -0.41 (-0.47 to -0.35) |
| Age-standardized Rates Males | 80.38(62.59-102.21) | 82.04(69.09-97.26) | -0.11 (-0.18 to -0.04) |
| Rate in the population 70+ | 591.31(392.48-868.78) | 511.08(386.74-707.54) | -0.59 (-0.68 to -0.51) |
| Rate in the population < 70 | 54.64(40.59-71.44) | 71.17(57.62-86.51) | 0.72 (0.62 to 0.82) |
| **PREVALENCE** |  |  |  |
| Absolute Numbers | 4930276(3885223-6301408) | 8410139(7063578-10013004) | 1.66(1.61 to 1.72) |
| Age-Standardized Rates | 817.69(649.57-1042.91) | 844.93(717.15-992.46) | -0.03 (-0.11 to 0.05) |
| Age-standardized Rates Females | 627.50(484.18-812.02) | 603.54(498.95-730.41) | -0.23 (-0.3 to -0.15) |
| Age-standardized Rates Males | 1064.26(853.19-1338.18) | 1118.59(968.90-1305.33) | 0.00 (-0.08 to 0.09) |
| Rate in the population 70+ | 8883.67(6440.55-11565.05) | 9258.24(7495.35 -11273.92) | 0.09 (0.04 to 0.13) |
| Rate in the population < 70 | 464.64(355.32-607.63) | 623.94(506.21-756.51) | 0.83 (0.7 to 0.96) |
| **DEATH** |  |  |  |
| Absolute Numbers | 31335(27682-33261) | 72184(58846-79292) | 3.01 (2.92 to 3.09) |
| Age-Standardized Rates | 5.43(4.75-5.77) | 5.52(4.56-6.04) | 0.31 (0.24 to 0.38) |
| Age-standardized Rates Females | 5.38(4.62-5.78) | 5.32(4.20-5.93) | 0.20 (0.13 to 0.26) |
| Age-standardized Rates Males | 5.36(4.91-5.58) | 5.75(5.05-6.10) | 0.51 (0.43 to 0.58) |
| Rate in the population 70+ | 78.05(68.41-83.08) | 105.68(85.47-116.37) | 1.27 (1.17 to 1.37) |
| Rate in the population < 70 | 0.63(0.61-0.65) | 0.71(0.68-0.74) | 0.75 (0.59 to 0.91) |
| **DALYs** |  |  |  |
| Absolute Numbers | 779576(634739-949783) | 1440284(1208224-1709735) | 2.13 (2.1 to 2.17) |
| Age-Standardized Rates | 130.74(106.98-159.03) | 131.15(108.91-156.67) | 0.08 (0.03 to 0.12) |
| Age-standardized Rates Females | 112.30(93.00-135.67) | 106.7(88.75-127.28) | -0.08 (-0.12 to -0.04) |
| Age-standardized Rates Males | 153.06(122.79-188.41) | 158.64(129.97-190.72) | 0.16 (0.11 to 0.22) |
| Rate in the population 70+ | 1574.01(1291.55 -1914.85) | 1793.24(1499.79-2129.74) | 0.59 (0.53 to 0.65) |

Given epidemiological measures include absolute numbers, age standardized rates per 100,000 population, age standardized rates for the female and male population per 100,000 females and males, respectively, rates in the population 70 years or older per100,000 and rates in the population under the age of 70, per 100,000. All of these have been given with their uncertainty intervals (in parenthesis).

*Western Europe = Andorra, Austria, Belgium, Cyprus, Denmark, Finland, France, Germany, Greece, Iceland, Ireland, Israel, Italy, Luxembourg,

DALYs: Disability adjusted life years; EAPC: Estimated annual percentage change. UI: Uncertainty intervals.

Table S5 Burden of atrial fibrillation/flutter in the Eastern a Europe in 1990 and 2021

|  | **Eastern Europe**** | | |
| --- | --- | --- | --- |
|  | **1990**  **n (95% UI)** | **2021**  **n (95% UI)** | **1990-2021 EAPC (95% CI)** |
| **INCIDENCE** |  |  |  |
| Absolute Numbers | 130200(98552-170809) | 176794(133417-231758) | 0.90 (0.86 to 0.94) |
| Age-Standardized Rates | 46.52(35.77-60.37) | 50.47(38.83-65.64 | 0.30 (0.24 to 0.37) |
| Age-standardized Rates Females | 41.44(31.24- 54.72) | 44.88(34.09-59.13) | 0.28 (0.23 to 0.33) |
| Age-standardized Rates Males | 53.05(41.24-69.19) | 57.48(44.34-74.49) | 0.32 (0.24 to 0.4) |
| Rate in the population 70+ | 289.50(176.17-428.78) | 303.15(188.17-437.61) | 0.1 (0.05 to 0.15) |
| Rate in the population < 70 | 40.85(30.03-55.29) | 60.43(44.12-80.4) | 1.18 (1.1 to 1.26) |
| **PREVALENCE** |  |  |  |
| Absolute Numbers | 1601348(1245865-2080667) | 2327313(1805305-3029889) | 1.22 (1.17 to 1.28) |
| Age-Standardized Rates | 584.41(454.97-753.25) | 648.92(507.00 -836.25 | 0.39(0.32 to 0.47) |
| Age-standardized Rates Females | 500.27(379.63-650.88) | 549.92(420.41-715.56) | 0.34 (0.28 to 0.4) |
| Age-standardized Rates Males | 730.24(572.97-953.20) | 799.39(627.10-1039.83) | 0.37 (0.28 to 0.47) |
| Rate in the population 70+ | 5460.07(3924.26-7381.12) | 6037.47(4441.35-8082.67) | 0.50 (0.43 to 0.57) |
| Rate in the population < 70 | 368.23(278.28-486.20) | 559.68(414.69-752.02) | 1.22(1.10 to 1.33) |
| **DEATH** |  |  |  |
| Absolute Numbers | 8133(7308-8953) | 15697(14042-17056) | 2.12 (1.94 to 2.29) |
| Age-Standardized Rates | 3.87(3.44-4.30) | 4.33(3.87-4.71) | 0.26 (0.15 to 0.37) |
| Age-standardized Rates Females | 3.86(3.44-4.23) | 4.30(3.76-4.75) | 0.21 (0.09 to 0.32) |
| Age-standardized Rates Males | 3.94(3.39-4.52) | 4.36(3.91-4.76) | 0.31 (0.19 to 0.43) |
| Rate in the population 70+ | 46.70(42.04-51.30) | 65.80(58.20-71.69) | 1.16 (0.79 to 1.53) |
| Rate in the population < 70 | 0.50(0.45-0.56) | 0.88(0.81-0.96) | 1.29 (1.02 to 1.57) |
| **DALYs** |  |  |  |
| Absolute Numbers | 242292(195940- 303226) | 388580(318353-470379) | 1.51 (1.46 to 1.56) |
| Age-Standardized Rates | 95.36(78.74-118.00) | 107.73(88.25-130.33) | 0.36 (0.27 to 0.44) |
| Age-standardized Rates Females | 88.17(74.08 -107.87) | 98.18(80.81-118.08) | 0.27 (0.18 to 0.35) |
| Age-standardized Rates Males | 107.58(84.96-136.50) | 121.52(98.51-148.59) | 0.43 (0.33 to 0.52) |
| Rate in the population 70+ | 993.40(833.67 -1211.12) | 1217.7(1020.01-1456.24) | 0.76 (0.59 to 0.93) |
| Rate in the population < 70 | 43.43(32.13- 58.27 | 69.29(51.37-91.92) | 1.28 (1.14 to 1.42) |

Given epidemiological measures include absolute numbers, age standardized rates per 100,000 population, age standardized rates for the female and male population per 100,000 females and males, respectively, rates in the population 70 years or older per100,000 and rates in the population under the age of 70, per 100,000. All of these have been given with their uncertainty intervals (in parenthesis).

**Eastern Europe = Belarus, Estonia, Latvia, Lithuania, Republic of Moldova, Russian Federation, Ukraine.

DALYs: Disability adjusted life years; EAPC: Estimated annual percentage change. UI: Uncertainty intervals.

Table S6 Burden of atrial fibrillation/flutter in the Central Europe in 1990 and 2021

|  | **Central Europe***** | | |
| --- | --- | --- | --- |
|  | **1990**  **n (95%UI)** | **2021**  **n (95% UI)** | **1990-2021 EAPC (95% CI)** |
| **INCIDENCE** |  |  |  |
| Absolute Numbers | 76656(57337- 101380) | 122625(94472-154039) | 1.26(1.18 to 1.35) |
| Age-Standardized Rates | 50.47(38.22-65.71) | 55.16(44.12-68.47) | 0.09 (0.03 to 0.16) |
| Age-standardized Rates Females | 43.71(32.37- 57.30) | 45.89(35.74-57.61) | -0.04 (-0.1 to 0.02) |
| Age-standardized Rates Males | 58.15(44.70-74.99) | 65.48(52.38-80.86) | 0.18 (0.1 to 0.26) |
| Rate in the population 70+ | 338.02(211.62 -495.36) | 355.90(255.67-478.81) | -0.16 (-0.28 to -0.05) |
| Rate in the population < 70 | 42.63(31.80- 56.56) | 69.50(53.41- 87.41) | 1.44 (1.31 to 1.57) |
| **PREVALENCE** |  |  |  |
| Absolute Numbers | 901372(699705- 1154305 | 1585128(1281857-1949451) | 1.65 (1.57 to 1.72) |
| Age-Standardized Rates | 616.69(478.07-790.30) | 679.00(554.31-827.23) | 0.07 (-0.01 to 0.15) |
| Age-standardized Rates Females | 502.46(384.93-655.21) | 531.11(426.95-657.11) | -0.08 (-0.16 to 0) |
| Age-standardized Rates Males | 769.42(608.45-983.66) | 868.00(715.84-1062.10) | 0.15 (0.06 to 0.24) |
| Rate in the population 70+ | 6360.46(4585.4-8539.31) | 7038.37(5409.80-8948.21) | 0.21 (0.17 to 0.26) |
| Rate in the population < 70 | 340.42(256.44-458.83) | 538.06(428.08-693.62) | 1.34 (1.19 to 1.49) |
| **DEATH** |  |  |  |
| Absolute Numbers | 5931(5508-6253) | 11104(9859-11914) | 2.41 (2.25 to 2.57) |
| Age-Standardized Rates | 5.10(4.66-5.40) | 4.55(4.03-4.88) | -0.21 (-0.41 to -0.02) |
| Age-standardized Rates Females | 4.92(4.48-5.28) | 4.29(3.76-4.66) | -0.31 (-0.51 to -0.11) |
| Age-standardized Rates Males | 5.46(4.99-5.91 | 4.97(4.49- 5.33) | -0.1 (-0.3 to 0.1) |
| Rate in the population 70+ | 64.33(59.37-67.90) | 68.55(60.53-73.55) | 0.68 (0.5 to 0.86) |
| Rate in the population < 70 | 0.72(0.69-0.77) | 0.92(0.84-1.00) | 1.05 (0.88 to 1.22) |
| **DALYs** |  |  |  |
| Absolute Numbers | 157482(132449-188984) | 197481(167999-231908) | 1.79 (1.68 to 1.91) |
| Age-Standardized Rates | 125.89(105.88-151.08) | 112.31(94.56-134.27) | -0.11 (-0.25 to 0.02) |
| Age-standardized Rates Females | 133.80(113.09-158.49) | 95.61(80.56-112.78) | -0.27 (-0.41 to -0.14) |
| Age-standardized Rates Males | 117.65(96.04-144.42) | 134.20(111.52 -162.12) | 0.02 (-0.11 to 0.16) |
| Rate in the population 70+ | 1287.92(1103.56-1532.80) | 1330.33(1131.73-1562.25) | 0.31 (0.21 to 0.42) |
| Rate in the population < 70 | 47.57(36.58-60.51) | 68.15(52.29-87.76) | 1.21 (1.1 to 1.32) |

Given epidemiological measures include absolute numbers, age standardized rates per 100,000 population, age standardized rates for the female and male population per 100,000 females and males, respectively, rates in the population 70 years or older per100,000 and rates in the population under the age of 70, per 100,000. All of these have been given with their uncertainty intervals (in parenthesis).

***Central Europe = Albania, Bosnia & Herzegovina, Bulgaria, Croatia, Czech Republic, Hungary, Montenegro, North Macedonia, Poland, Romania, Serbia,

Slovakia, Slovenia.

DALYs: Disability adjusted life years; EAPC: Estimated annual percentage change. UI: Uncertainty intervals.

Table S7 Atrial fibrillation/flutter in EU-53: Incidence by country, sex, and age

|  | **Incidence** |  |  |
| --- | --- | --- | --- |
|  | **1990**  **n (95% UI)** | **2021**  **n (95% UI)** | **1990-2021EAPC**  **(95% CI)** |
| **WESTERN EUROPE** | | | |
| **Andorra** |  |  |  |
| Absolute Numbers | 41(30-54) | 91.07(68.46-118.61) | 2.11 (1.87 to 2.34) |
| Age-Standardized Rates | 69.12(51.57-91.70) | 60.36(45.23-78.41) | -0.56 (-0.62 to -0.51) |
| Female | 55.80 (40.75-75.98) | 47.37(34.57-64.07) | -0.69 (-0.76 to -0.62) |
| Male | 80.47(60.44-105.21) | 71.85(53.67-93.37) | -0.48 (-0.53 to -0.43) |
| Population 70+ | 580.03(376.59-857.84) | 467.40(308.69-669.54) | -0.87 (-0.95 to -0.79) |
| Population < 70 | 43.27(31.63-57.61) | 62.23(44.90-83.16) | 0.99 (0.81 to 1.17) |
| **Austria** |  |  |  |
| Absolute Numbers | 7033(6199-8047) | 18808(17782-19775) | 3.54 (3.41 to 3.68) |
| Age-Standardized Rates | 57.77(51.28-65.36) | 105.58(100.49-110.74) | 2.18 (2.05 to 2.31) |
| Female | 45.97(40.68-53.24) | 80.88(76.39-85.22) | 2.07 (1.96 to 2.19) |
| Male | 72.45(63.51-82.81) | 131.93(124.12-139.04) | 2.20 (2.05 to 2.35) |
| Population 70+ | 541.96(444.58-673.11) | 920.54(831.04-1001.40) | 2.06 (1.92 to 2.20) |
| Population < 70 | 41.41(34.97-49.24) | 95.07(87.73 -103.88) | 3.09 (2.86 to 3.32) |
| **Belgium** |  |  |  |
| Absolute Numbers | 9867(7416-13339) | 11602(8964-15260) | 0.30 (0.17 to 0.44) |
| Age-Standardized Rates | 62.56(47.73-83.44) | 53.01(41.85-67.78) | -0.72 (-0.84 to -0.6) |
| Female | 52.72(38.92 -72.59) | 42.83(32.92 -56.29) | -0.91 (-1.06 to -0.76) |
| Male | 71.73(54.30-94.48) | 63.04(50.06-80.45) | -0.61 (-0.73 to -0.49) |
| Population 70+ | 589.51(384.79-873.83) | 413.21(284.47-598.46) | -1.42 (-1.59 to -1.25) |
| Population < 70 | 46.60(32.88-64.97) | 50.28(37.10-65.39) | 0.13(-0.03 to 0.30) |
| **Cyprus** |  |  |  |
| Absolute Numbers | 477(347-625) | 928(720-1217.88) | 2.23 (2.05 to 2.42) |
| Age-Standardized Rates | 55.82(42.00-72.70) | 42.95(34.02-55.26) | -0.82 (-0.9 to -0.73) |
| Female | 46.39(34.33-61.98) | 32.15(25.08-43.10) | -1.17 (-1.29 to -1.05) |
| Male | 65.39(49.54-84.50) | 54.28(42.65-68.81) | -0.58 (-0.66 to -0.51) |
| Population 70+ | 484.69(301.16-726.36) | 411.16(283.64-608.09) | -0.74 (-0.88 to -0.61) |
| Population < 70 | 31.20(23.17-41.04) | 30.42(22.78-39.26) | 0.90 (0.68 to 1.13) |
| **Denmark** |  |  |  |
| Absolute Numbers | 5649(4210-7572) | 7912(5907-10323) | 0.75 (0.64 to 0.86) |
| Age-Standardized Rates | 68.96(52.48-89.77) | 70.03(54.45-89.03) | -0.32 (-0.5 to -0.14) |
| Female | 55.52(41.41-73.62) | 55.82(42.65-73.46) | -0.32 (-0.46 to -0.18) |
| Male | 82.36(62.80-107.62) | 83.89(65.39-104.34) | -0.46 (-0.66 to -0.26) |
| Population 70+ | 577.91(384.94-832.52) | 502.20(331.30-730.89) | -0.97 (-1.13 to -0.80) |
| Population < 70 | 52.76(37.51-70.54) | 72.01(55.18-92.09) | 0.90 (0.68 to 1.13) |
| **Finland** |  |  |  |
| Absolute Numbers | 6773(4959-8593) | 8476(6391-11009) | 0.62 (0.52 to 0.72) |
| Age-Standardized Rates | 94.77(70.63-119.71) | 69.89(55.04-86.68) | -1.13 (-1.21 to -1.04) |
| Female | 76.41(54.03-101.22) | 55.57(42.25-71.59) | -1.22 (-1.31 to -1.13) |
| Male | 112.87(85.82-142.27) | 83.88(67.25-102.03) | -1.11 (-1.2 to -1.02) |
| Population 70+ | 668.21(425.97-988.33) | 517.12(346.29-766.17) | -1.04 (-1.13 to -0.95) |
| Population < 70 | 81.64(58.33-106.60) | 78.96(62.66-95.30) | -0.02 (-0.24 to 0.20) |
| **France** |  |  |  |
| Absolute Numbers | 57571(42070-77071) | 78372(57303-105675) | 0.89 (0.82 to 0.96) |
| Age-Standardized Rates | 68.85(51.40-90.39) | 59.15(44.34-78.68) | -0.62 (-0.69 to -0.56) |
| Female | 56.57(40.63-77.07) | 47.13(33.86-63.44) | -0.77 (-0.85 to -0.69) |
| Male | 81.09(61.45-105.11) | 71.65(54.44-93.76) | -0.92 (-0.97 to -0.86) |
| Population 70+ | 590.63(370.26-881.07) | 460.05(300.08-672.57) | 0.41 (0.25 to 0.58) |
| Population < 70 | 51.05(36.24-69.27) | 57.88(40.69-79.15) | 0.41 (0.25 to 0.58) |
| **Germany** |  |  |  |
| Absolute Numbers | 101466(75243-135577) | 140086(122558-158105) | 1.01 (0.87 to 1.16) |
| Age-Standardized Rates | 79.75(60.18-104.73) | 81.28(72.34-89.74) | -0.02 (-0.16 to 0.11) |
| Female | 62.48(45.16-84.36) | 55.60(47.89-62.99) | -0.32 (-0.42 to -0.22) |
| Male | 99.09(73.36-128.64) | 107.70(95.76- 118.94) | -0.53 (-0.6 to -0.47) |
| Population 70+ | 658.24(420.25-970.50) | 481.68(393.51-624.79) | -0.93 (-1.06 to -0.80) |
| Population < 70 | 67.15(48.26-89.57) | 104.14(85.22- 119.21) | 1.22 (0.83 to 1.61) |
| **Greece** |  |  |  |
| Absolute Numbers | 8833(7049-11378) | 12367(9012-16893) | 1.00 (0.73 to 1.27) |
| Age-Standardized Rates | 57.29 (45.98-72.87) | 54.67(41.00-71.66) | -0.30 (-0.50 to -0.10) |
| Female | 49.75(37.27-66.81) | 45.00(32.38-61.79) | -0.69 (-0.95 to -0.42) |
| Male | 65.04(54.34-82.01) | 64.72(49.34-84.17) | -0.05 (-0.23 to 0.12) |
| Population 70+ | 486.20(337.68-718.45) | 436.34(284.30-632.39) | -0.44 (-0.58 to -0.29) |
| Population < 70 | 44.77(35.25-57.40) | 57.00(40.17-76.85) | 0.38 (0.16 to 0.61) |
| **Iceland** |  |  |  |
| Absolute Numbers | 175(129- 230) | 375( 296-464) | 2.35 (2.29 to 2.42) |
| Age-Standardized Rates | 60.61(45.01-78.80) | 65.91(52.74-81.71) | 0.18 (0.10 to 0.25) |
| Female | 46.39(33.40-62.31) | 46.65(36.18-58.64) | -0.05 (-0.13 to 0.03) |
| Male | 75.21(55.66-97.47) | 84.95(67.93-104.16) | 0.25 (0.16 to 0.35) |
| Population 70+ | 522.51(339.87-770.80) | 543.97(386.05-757.13) | 0.10 (-0.06 to 0.26) |
| Population < 70 | 33.72(24.07-44.58) | 54.18(40.52-68.89) | 1.44 (1.32 to 1.57) |
| **Ireland** |  |  |  |
| Absolute Numbers | 2713(1936-3616) | 4292(3141-5685) | 1.08 (0.81 to 1.34) |
| Age-Standardized Rates | 64.43(47.57-84.46) | 54.42(40.84-71.55) | -0.99 (-1.15 to -0.83) |
| Female | 51.39(36.83-69.80) | 41.86(30.43-57.36) | -1.43 (-1.7 to -1.17) |
| Male | 77.30(58.29-100.76) | 67.16(50.74-86.86) | -0.83 (-0.92 to -0.73) |
| Population 70+ | 546.62(354.55-816.02) | 437.16(285.23-644.39) | -1.41 (-1.65 to -1.17) |
| Population < 70 | 37.33(26.58-49.70) | 45.68(32.95-61.51) | 0.3 (0.15 to 0.45) |
| **Israel** |  |  |  |
| Absolute Numbers | 3551(2614-4680) | 11110(8995-12780) | 4.06 (3.82 to 4.29) |
| Age-Standardized Rates | 70.89(53.65-92.18) | 91.98(75.23-105.16) | 1.20 (0.95 to 1.45) |
| Female | 58.87(42.68-79.56) | 79.68(58.89-96.90) | 1.47 (1.12 to 1.82) |
| Male | 84.47(64.29-108.83) | 104.34(90.28- 116.24) | 1.02 (0.80 to 1.23) |
| Population 70+ | 608.33(395.63-906.28) | 681.77(509.38-867.74) | 0.70 (0.46 to 0.95) |
| Population < 70 | 36.53(26.25-48.58) | 62.51(49.77-73.21) | 2.47 (2.13 to 2.81) |
| **Italy** |  |  |  |
| Absolute Numbers | 68691(50618-91262) | 99307(72177-134792) | 0.83 (0.66 to 1.00) |
| Age-Standardized Rates | 76.05(57.71-99.91) | 70.54(52.49-92.64) | -0.62 (-0.78 to -0.45) |
| Female | 73.33(53.83-96.91) | 65.15(47.65-86.83) | -0.69 (-0.78 to -0.60) |
| Male | 76.55(58.46-99.59) | 75.32(57.35-97.60) | -0.61 (-0.88 to -0.35) |
| Population 70+ | 637.28(410.81-941.58) | 592.67(392.35-875.85) | -0.74 (-0.98 to -0.51) |
| Population < 70 | 65.14(46.26-87.97) | 75.01(52.54-102.09) | 0.05 (-0.10 to 0.21) |
| **Luxembourg** |  |  |  |
| Absolute Numbers | 380( 310-475) | 681(580-770) | 1.91 (1.81 to 2.01) |
| Age-Standardized Rates | 67.74(56.09-83.07) | 65.48(56.11-73.74) | -0.08 (-0.11 to -0.05) |
| Female | 57.27(46.11-73.18) | 49.98(44.02-55.92) | -0.38 (-0.44 to -0.32) |
| Male | 77.86(65.23-91.57) | 80.58(66.61-93.92) | 0.07 (0.03 to 0.12) |
| Population 70+ | 620.29(454.71-870.91) | 576.40(465.91-703.36) | -0.16 (-0.23 to -0.09) |
| Population < 70 | 48.39(39.16-58.62) | 51.48(42.79-61.37) | 0.11 (-0.06 to 0.27) |
| **Malta** |  |  |  |
| Absolute Numbers | 251(181-332) | 185(149-221) | 2.69 (2.35 to 3.02) |
| Age-Standardized Rates | 57.92(42.47-76.67) | 51.15(44.57-58.00) | -0.09 (-0.38 to 0.2) |
| Female | 48.13(34.61-65.34) | 45.19(39.26-50.48) | 0.11 (-0.21 to 0.43) |
| Male | 68.38(51.40-88.85) | 56.76(48.45-66.29) | -0.31 (-0.58 to -0.05) |
| Population 70+ | 502.57(322.31-744.98) | 455.76(369.70-564.18) | -0.07 (-0.35 to 0.22) |
| Population < 70 | 36.45(26.95-48.09) | 49.96(40.33-59.61) | 1.76 (1.42 to 2.10) |
| **Monaco** |  |  |  |
| Absolute Numbers | 47(34-63) | 55(40-74) | 0.34 (0.23 to 0.44) |
| Age-Standardized Rates | 66.75(49.53-87.83) | 58.82(44.19-76.65) | -0.53 (-0.59 to -0.47) |
| Female | 54.81(39.68-73.63) | 46.64(33.62-62.53) | -0.66 (-0.73 to -0.58) |
| Male | 78.99(59.14-103.28) | 71.22(54.02-92.19) | -0.43 (-0.48 to -0.38) |
| Population 70+ | 567.32(362.17-835.60) | 466.43(303.07-693.96) | -0.78 (-0.86 to -0.69) |
| Population < 70 | 73.39(50.80- 100.02) | 71.94(49.57-98.90) | 0.03(-0.09 to 0.15) |
| **Netherlands** |  |  |  |
| Absolute Numbers | 14709(13196-16558) | 22455(18709-27075) | 1.02 (0.86 to 1.18) |
| Age-Standardized Rates | 72.21(64.26-80.62) | 64.02(54.48-75.58) | -0.75 (-0.89 to -0.60) |
| Female | 65.06 (57.81-73.36) | 56.86(45.78-69.37) | -0.83 (-0.98 to -0.67) |
| Male | 77.98(69.84-87.75) | 70.81（61.84-82.80） | -0.63 (-0.76 to -0.50) |
| Population 70+ | 637.75(525.81-784.16) | 535.51(411.45-713.42) | -0.95 (-1.11 to -0.79) |
| Population < 70 | 47.86(41.15-54.68) | 63.06(51.02-75.88) | 0.80 (0.72 to 0.89) |
| **Norway** |  |  |  |
| Absolute Numbers | 4758(3431-6405) | 6082(4530-8012) | 0.69 (0.54 to 0.85) |
| Age-Standardized Rates | 69.29(52.99-90.88) | 62.72(47.43-82.17) | -0.37 (-0.40 to -0.33) |
| Female | 55.44(40.57-74.72) | 47.15(34.62-62.74) | -0.62 (-0.67 to -0.56) |
| Male | 82.96(63.30-108.35) | 77.47(58.57-100.63) | -0.24 (-0.27 to -0.20) |
| Population 70+ | 575.35(376.18-834.93) | 466.29(302.93-672.35) | -0.85 (-0.93 to -0.77) |
| Population < 70 | 51.82(36.56-70.03) | 60.38(43.27-81.24) | 0.85 (0.69 to 1.01) |
| **Portugal** |  |  |  |
| Absolute Numbers | 9401(6872-12570) | 14593(11898-18076) | 1.53 (1.45 to 1.61) |
| Age-Standardized Rates | 65.52(49.07-86.59) | 59.99(49.76-72.76) | -0.24 (-0.32 to -0.16) |
| Female | 59.32(43.19-79.96) | 56.01(45.49-68.82) | -0.12 (-0.16 to -0.08) |
| Male | 70.99(53.88-92.55) | 63.62(53.37-76.85) | -0.34 (-0.47 to -0.20) |
| Population 70+ | 579.87(373.99-839.87) | 513.06(381.47-696.50) | -0.29 (-0.43 to -0.14) |
| Population < 70 | 47.60(33.66-64.94) | 62.39(49.37-77.70) | 0.88 (0.76 to 1.00) |
| **San Marino** |  |  |  |
| Absolute Numbers | 23.91(17.55-31.52) | 40.78(29.97-54.40) | 1.59 (1.53 to 1.65) |
| Age-Standardized Rates | 67.30(50.42-87.53) | 58.50(43.30-76.78) | -0.56 (-0.60 to -0.51) |
| Female | 54.93 (40.28-74.17) | 46.72(33.63-63.56) | -0.65 (-0.71 to -0.59) |
| Male | 79.36(59.57-103.08) | 71.29(53.14-93.12) | -0.44 (-0.48 to -0.40) |
| Population 70+ | 564.10(362.73-823.56) | 453.19(300.45-665.83) | -0.86 (-0.93 to -0.79) |
| Population < 70 | 50.11 (36.09-66.15) | 62.38(43.84-84.41) | 0.62 (0.52 to 0.72) |
| **Spain** |  |  |  |
| Absolute Numbers | 416745(31727-53592) | 64456(56846-73040) | 1.71 (1.53 to 1.89) |
| Age-Standardized Rates | 74.66(57.65-95.00) | 69.90(62.22-78.58) | -0.01 (-0.14 to 0.12) |
| Female | 66.68(50.16-86.70) | 56.89(49.51-65.70) | -0.44 (-0.58 to -0.30) |
| Male | 81.53(63.62-104.01) | 82.94(74.94-91.21) | 0.40 (0.24 to 0.56) |
| Population 70+ | 649.07(436.01-962.52) | 538.59(436.23-665.06) | -0.18 (-0.32 to -0.05) |
| Population < 70 | 54.63(40.85-70.99) | 72.23(61.31-83.87) | 0.82 (0.70 to 0.94) |
| **Sweden** |  |  |  |
| Absolute Numbers | 13182(9876-17727) | 25710(18631-33708) | 1.73 (1.53 to 1.92) |
| Age-Standardized Rates | 85.18(65.88-110.36) | 123.84(92.51-159.67) | 0.83 (0.7 to 0.96) |
| Female | 76.62(57.11-102.27) | 109.47(80.62- 142.62) | 0.67 (0.44 to 0.90) |
| Male | 92.26(71.52-116.79) | 136.71(102.20-174.39) | 0.98 (0.87 to 1.08) |
| Population 70+ | 773.40(518.06-1146.38) | 965.51(653.72-1386.71) | 0.09 (-0.09 to 0.28) |
| Population < 70 | 63.24(45.25-82.98) | 117.47(82.07- 159.97) | 2.11 (1.89 to 2.33) |
| **Switzerland** |  |  |  |
| Absolute Numbers | 4309(3281-5690) | 6346(5485-7579) | 1.74 (1.31 to 2.16) |
| Age-Standardized Rates | 40.72(31.61-52.93) | 36.45(31.83-42.53) | 0.09(-0.28 to 0.47) |
| Female | 32.58(24.80-43.33) | 27.55(23.25-33.63) | 0.02(-0.39 to 0.45) |
| Male | 32.58(24.80-43.33) | 45.35(40.02-51.96) | 0.05(-0.29 to 0.38) |
| Population 70+ | 368.16(257.67-538.95) | 293.94(237.11-385.63) | -0.28 (-0.62 to 0.05) |
| Population < 70 | 28.22(20.62-36.57) | 34.90(29.20-42.18) | 1.25 (0.84 to 1.66) |
| **United Kingdom** |  |  |  |
| Absolute Numbers | 48564(36703-63566) | 65549(51519-83607) | 0.78 (0.6 to 0.95) |
| Age-Standardized Rates | 53.06 (41.35-68.26) | 52.25(41.80-65.60) | -0.27 (-0.39 to -0.14) |
| Female | 41.58(31.18-54.71) | 40.68(31.83-52.42) | -0.32 (-0.47 to -0.18) |
| Male | 64.93(50.81-82.93) | 64.01(51.51-79.12) | -0.23 (-0.34 to -0.12) |
| Population 70+ | 438.61(297.10-639.87) | 408.86(290.24-581.85) | -0.48 (-0.61 to -0.35) |
| Population < 70 | 41.65(31.13-53.56) | 47.94(37.36-60.36) | 0.39 (0.29 to 0.50) |
| **CENTRAL EUROPE** | | | |
| **Albania** |  |  |  |
| Absolute Numbers | 995(742- 1295) | 2070(1507-2764) | 2.43 (2.38 to 2.49) |
| Age-Standardized Rates | 47.94(35.37-62.61) | 46.19(34.59-60.40) | 0.10 (-0.13 to -0.07) |
| Female | 39.97(28.74-53.02) | 38.25(27.91-50.95) | -0.13 (-0.16 to -0.1) |
| Male | 55.45(41.03-71.92) | 54.75(41.35-71.16) | -0.02 (-0.05 to 0.01) |
| Population 70+ | 328.55(206.78-465.37) | 300.91(183.92-432.90) | -0.27 (-0.3 to -0.23) |
| Population < 70 | 20.80(15.41-27.63) | 52.32(37.32-71.06) | 3.07 (3.05 to 3.10) |
| **Bosnia and Herzegovina** |  |  |  |
| Absolute Numbers | 1861(1414-2479) | 2895(2119-3855) | 1.28 (1.12 to 1.44) |
| Age-Standardized Rates | 45.79(34.25-60.36) | 87.66(64.15-116.72) | 0.03 (0.00 to 0.06) |
| Female | 38.74(28.21-51.60) | 81.29(56.55-109.69) | -0.05 (-0.07 to -0.02) |
| Male | 54.07(40.91-71.00) | 94.31(69.29-124.65) | 0.07 (0.03 to 0.10) |
| Population 70+ | 306.92(193.73-455.40) | 294.74(179.49-418.94) | -0.24 (-0.32 to -0.17) |
| Population < 70 | 31.09(22.92-42.38) | 60.04(42.83-80.27) | 1.68 (1.45 to 1.91) |
| **Bulgaria** |  |  |  |
| Absolute Numbers | 6135(4417-8233) | 3026(1901-4317) | 0.25 (0.22 to 0.27) |
| Age-Standardized Rates | 47.44(34.84-62.38) | 45.66(33.96-60.04) | -0.05 (-0.09 to -0.01) |
| Female | 39.96(29.12-53.13) | 37.94(27.75-50.62) | -0.10 (-0.13 to -0.06) |
| Male | 55.63(41.28-72.90) | 54.24(40.17-71.05) | -0.01 (-0.05 to 0.02) |
| Population 70+ | 325.09(196.43-480.35) | 293.97(184.65-419.34) | -0.35 (-0.39 to -0.32) |
| Population < 70 | 49.76(36.21-67.23) | 60.06(42.76-79.54) | 0.85 (0.72 to 0.97) |
| **Croatia** |  |  |  |
| Absolute Numbers | 1919(1584-2351) | 3045(2682-3466) | 3.11 (2.74 to 3.48) |
| Age-Standardized Rates | 30.99(25.33-38.17) | 34.78(30.93-39.18) | 0.86 (0.6 to 1.12) |
| Female | 25.93(19.81-33.67) | 22.92(20.89-25.24) | 0.21 (-0.13 to 0.55) |
| Male | 37.12(31.68-45.73) | 49.34(42.29-57.45) | 1.27 (1.09 to 1.44) |
| Population 70+ | 193.32(129.80-276.77) | 245.89(201.98-304.86) | 1.36 (1.07 to 1.65) |
| Population < 70 | 29.94(24.96-36.87) | 42.66(36.36-50.05) | 1.38 (1.11 to 1.64) |
| **Czech Republic** |  |  |  |
| Absolute Numbers | 7616(5591-10112) | 17905(14041-20271) | 3.11 (2.74 to 3.48) |
| Age-Standardized Rates | 54.70(40.90-71.71) | 81.97(65.98-91.63) | 1.50 (1.07 to 1.93) |
| Female | 46.51(33.84-62.14) | 65.73(51.50-75.16) | 1.29 (0.89 to 1.69) |
| Male | 64.30(48.62-83.62) | 98.99(79.96-110.67) | 1.59 (1.14 to 2.05) |
| Population 70+ | 349.89(218.22-510.73) | 591.18(422.77-750.62) | 1.75 (1.15 to 2.36) |
| Population < 70 | 50.82(36.47-67.87) | 96.56(78.90-111.74) | 2.71 (2.43 to 3.00) |
| **Hungary** |  |  |  |
| Absolute Numbers | 3055(1860-4446) | 8870(6408-11720) | 0.15 (0.06 to 0.23) |
| Age-Standardized Rates | 55.95(41.54-73.85) | 46.18(34.66-60.55) | -0.67 (-0.72 to -0.61) |
| Female | 47.57(34.34-62.90) | 38.54(28.36-51.59) | -0.73 (-0.79 to -0.68) |
| Male | 66.05(49.21-87.22) | 55.10(41.74-71.39) | -0.63 (-0.68 to -0.58) |
| Population 70+ | 360.32(219.45-524.45) | 291.39(181.36-419.71) | -0.85 (-0.91 to -0.79) |
| Population < 70 | 55.80(40.41-74.45) | 59.95(43.40-80.59) | 0.35 (0.19 to 0.5) |
| **Montenegro** |  |  |  |
| Absolute Numbers | 313( 235-408) | 484( 353-637) | 1.32 (1.21 to 1.43) |
| Age-Standardized Rates | 49.96(37.51-65.19) | 47.55(35.29-62.08) | -0.17 (-0.20 to -0.14) |
| Female | 47.08(34.13-62.29) | 39.53(28.73-52.44) | -0.24 (-0.28 to -0.21) |
| Male | 52.86(40.51-69.43) | 56.46(42.11-72.85) | -0.13 (-0.17 to -0.10) |
| Population 70+ | 327.36(201.01-472.86) | 309.11(188.92-441.73) | -0.26 (-0.33 to -0.19) |
| Population < 70 | 35.02(25.66-48.17) | 54.28(39.06-72.95) | 1.22 (1.12 to 1.32) |
| **North Macedonia** |  |  |  |
| Absolute Numbers | 914( 683-1207) | 1603(1158-2145) | 1.83 (1.79 to 1.86) |
| Age-Standardized Rates | 48.59(36.06-64.08) | 45.77(33.88-60.43) | -0.16 (-0.2 to -0.13) |
| Female | 40.92(29.75-54.62) | 37.74(27.30-50.44) | -0.24 (-0.27 to -0.2) |
| Male | 56.70(42.26-73.62) | 53.97(40.46-70.42) | -0.13 (-0.16 to -0.10) |
| Population 70+ | 328.80(205.83-474.41) | 307.76(183.90-443.68) | -0.31 (-0.34 to -0.27) |
| Population < 70 | 32.76(24.05-44.37) | 51.24(37.57-68.77) | 1.34 (1.24 to 1.44) |
| **Poland** |  |  |  |
| Absolute Numbers | 234167(17441-30849) | 30161(22089-40239) | 1.85 (1.59 to 2.11) |
| Age-Standardized Rates | 53.07(40.00-69.57) | 69.46(51.87-90.62) | 0.31 (0.04 to 0.58) |
| Female | 47.56(34.91-62.81) | 59.22(43.28-78.67) | 0.10(-0.13 to 0.33) |
| Male | 59.27(45.40-76.62) | 80.91(60.73-104.76) | 0.27(-0.02 to 0.56) |
| Population 70+ | 367.16(228.82-542.52) | 425.30(264.35-591.53) | -0.19 (-0.41 to 0.03) |
| Population < 70 | 40.77(29.95-54.23) | 89.85(65.80-119.87) | 1.86 (1.58 to 2.14) |
| **Romania** |  |  |  |
| Absolute Numbers | 13355(9778-17807) | 12515(10602-15109) | -0.41 (-0.62 to -0.2) |
| Age-Standardized Rates | 47.05(34.77-61.61) | 33.74(29.07-40.10) | -1.19 (-1.43 to -0.95) |
| Female | 39.91(28.62-52.76) | 26.83(22.65-32.95) | -1.44 (-1.74 to -1.14) |
| Male | 55.09(41.46-72.18) | 41.92(36.09-48.71) | -1.00 (-1.22 to -0.79) |
| Population 70+ | 316.90(191.52-476.02) | 223.32(173.39-287.73) | -1.38 (-1.56 to -1.21) |
| Population < 70 | 40.24(29.57-54.07) | 57.92(42.28-76.36) | -0.07 (-0.46 to 0.33) |
| **Serbia** |  |  |  |
| Absolute Numbers | 5307(3932-7011) | 5688(4597-7029) | 0.05(-0.16 to 0.26) |
| Age-Standardized Rates | 45.39(33.82-59.77) | 34.05(28.15-41.75) | -0.99 (-1.22 to -0.75) |
| Female | 38.48(27.92-51.60) | 28.13(23.08-35.07) | -1.07 (-1.33 to -0.81) |
| Male | 52.97(39.79-68.53) | 40.24(32.52-50.65) | -0.96 (-1.22 to -0.71) |
| Population 70+ | 315.24(195.78-453.47) | 246.82(182.12-333.48) | -0.94 (-1.11 to -0.77) |
| Population < 70 | 40.96(30.09-55.46) | 61.58(44.58-81.98) | -0.32 (-0.68 to 0.05) |
| **Slovakia** |  |  |  |
| Absolute Numbers | 3990(3313-4629) | 7231(5730-8496) | 1.80 (1.71 to 1.89) |
| Age-Standardized Rates | 65.82(55.05-75.92) | 73.15(58.90-85.48) | 0.27 (0.19 to 0.34) |
| Female | 57.71(48.14-66.64) | 67.17(52.39-78.89) | 0.38 (0.29 to 0.47) |
| Male | 57.71(48.14-66.64) | 78.51(61.96-95.92) | 0.08 (-0.04 to 0.21) |
| Population 70+ | 344.25(251.79-461.93) | 452.52(331.57-573.99) | 0.5 (0.32 to 0.68) |
| Population < 70 | 40.96(30.09-55.46) | 93.42(73.93-112.60) | 1.69 (1.6 to 1.79) |
| **Slovenia** |  |  |  |
| Absolute Numbers | 1229(919.98- 1632.17) | 2024(1731-2316) | 1.56 (1.5 to 1.62) |
| Age-Standardized Rates | 50.16(37.53-66.24) | 48.42(42.05-54.65) | -0.1 (-0.15 to -0.05) |
| Female | 42.31(30.84-56.85) | 36.96(31.85-42.14) | -0.35 (-0.43 to -0.27) |
| Male | 59.78(45.09-77.41) | 60.17(51.65-68.26) | -0.02 (-0.07 to 0.02) |
| Population 70+ | 321.71(199.22-479.08) | 278.70(218.15-354.60) | -0.54 (-0.64 to -0.44) |
| Population < 70 | 57.58(46.74-67.72) | 67.09(55.88-77.48) | 1.32 (1.22 to 1.41) |
| **EASTERN EUROPE** | | | |
| **Belarus** |  |  |  |
| Absolute Numbers | 5642(4239-7454) | 7544(5606-9888) | 0.76 (0.7 to 0.83) |
| Age-Standardized Rates | 43.56(32.82-56.35) | 265.92(163.61-382.07) | 0.25 (0.21 to 0.29) |
| Female Rates | 37.31(27.70-48.90) | 39.90(29.79-52.18) | 0.20 (0.16 to 0.24) |
| Male | 51.82(39.61-67.42) | 56.76(43.90-73.74) | 0.28 (0.24 to 0.33) |
| Population 70+ | 254.67(154.96-379.07) | 265.92(163.61-382.07) | 0.00 (-0.11 to 0.11) |
| Population < 70 | 39.70(29.35-53.92) | 59.82(44.12-80.37) | 1.13 (0.97 to 1.29) |
| **Estonia** |  |  |  |
| Absolute Numbers | 871(648- 1141) | 1149(859-1498) | 0.95 (0.91 to 0.98) |
| Age-Standardized Rates | 42.66(32.14-54.94) | 46.91(35.52-60.22) | 0.36 (0.32 to 0.40) |
| Female | 36.60(26.70-48.18) | 39.35(29.46-51.50) | 0.29 (0.25 to 0.33) |
| Male | 50.76(39.34-65.89) | 55.87(43.46-71.65) | 0.36 (0.32 to 0.40) |
| Population 70+ | 251.70(152.14-374.50) | 259.25(163.09-375.71) | 0.08 (-0.01 to 0.16) |
| Population < 70 | 39.50(28.92-53.15) | 58.34(42.61-78.40) | 1.19 (1.14 to 1.24) |
| **Latvia** |  |  |  |
| Absolute Numbers | 1446(1077-1904) | 1701(1429-1990) | 0.79 (0.61 to 0.97) |
| Age-Standardized Rates | 40.60(30.59-52.86) | 47.40(40.34-54.56) | 0.80 (0.61 to 1.00) |
| Female | 34.16(25.27-45.63) | 37.52(31.96-43.46) | 0.70 (0.48 to 0.93) |
| Male | 49.54(37.91-64.24) | 60.14(50.64-69.21) | 0.93 (0.78 to 1.08) |
| Population 70+ | 241.63(146.70-355.02) | 247.39(182.66-327.13) | 0.45 (0.25 to 0.66) |
| Population < 70 | 38.55(28.29-52.06) | 58.34(42.61-78.40) | 1.83 (1.64 to 2.01) |
| **Lithuania** |  |  |  |
| Absolute Numbers | 1975(1485-2581) | 2548(1918-3354) | 0.84 (0.74 to 0.94) |
| Age-Standardized Rates | 43.97(33.46-57.27) | 48.23(36.45-62.39) | 0.35 (0.31 to 0.39) |
| Female | 37.69(27.60-50.41) | 40.77(30.38-53.95) | 0.32 (0.28 to 0.36) |
| Male | 52.10(39.97-68.15) | 57.54(44.15-74.42) | 0.36 (0.32 to 0.41) |
| Population 70+ | 257.83(158.95-386.53) | 265.27(168.85-396.15) | 0.10 (0.01 to 0.19) |
| Population < 70 | 38.59 (27.86-52.34) | 63.74(46.67-85.11) | 1.63 (1.58 to 1.68) |
| **Republic of Moldova** |  |  |  |
| Absolute Numbers | 1925(1459-2517) | 2822(2114-3649) | 1.19 (1.16 to 1.23) |
| Age-Standardized Rates | 43.01(32.70-55.93) | 47.44(36.08-60.91) | 0.36 (0.34 to 0.37) |
| Female | 36.76(27.24-48.66) | 39.93(29.68-52.45) | 0.31 (0.30 to 0.32) |
| Male | 51.03(39.38-66.19) | 56.54(43.66-72.24) | 0.38 (0.36 to 0.40) |
| Population 70+ | 261.70(156.44-388.87) | 270.39(166.91-386.68) | 0.05 (0.00 to 0.10) |
| Population < 70 | 32.46(23.93-43.57) | 57.92(42.28-76.36) | 1.93 (1.79 to 2.07) |
| **Russian Federation** |  |  |  |
| Absolute Numbers | 86356(65246-113303) | 125499(94653- 165367) | 1.13 (1.08 to 1.17) |
| Age-Standardized Rates | 47.79(36.77-62.21) | 52.32(40.22-68.20) | 0.33 (0.25 to 0.40) |
| Female | 42.85(32.41-56.40) | 46.92(35.63-61.57) | 0.29 (0.24 to 0.35) |
| Male | 54.10(41.94-70.31) | 59.04(45.47-76.76) | 0.35 (0.25 to 0.45) |
| Population 70+ | 298.26(180.92-442.77) | 315.27(195.68-454.27) | 0.11 (0.06 to 0.16) |
| Population < 70 | 40.73(30.01-54.87) | 61.58(44.58-81.98) | 1.25 (1.18 to 1.32) |
| **Ukraine** |  |  |  |
| Absolute Numbers | 31985(24201-42017) | 35530(26656-46895) | 0.28 (0.25 to 0.30) |
| Age-Standardized Rates | 44.68(34.28-57.82) | 46.19 (35.34-60.16) | 0.15 (0.11 to 0.18) |
| Female | 39.63(29.62-51.91) | 40.84(30.71-53.83) | 0.17 (0.12 to 0.22) |
| Male | 51.39(39.55-67.22) | 52.96(40.56-68.69) | 0.10 (0.08 to 0.12) |
| Population 70+ | 281.51(171.89-415.54) | 285.31(176.44-426.43) | 0.07 (0.02 to 0.13) |
| Population < 70 | 42.50(30.83-57.83) | 56.59(41.02-75.44) | 0.79 (0.66 to 0.92) |
| **NO DEFINED SUBREGION** | | | |
| **Armenia** |  |  |  |
| Absolute Numbers | 1140(872-1502) | 2013(1479-2662) | 1.76 (1.67 to 1.86) |
| Age-Standardized Rates | 43.01(32.14-56.20) | 45.65(34.23-60.63) | 0.21 (0.19 to 0.23) |
| Female | 37.82(27.40-49.45) | 39.68(29.11-52.58) | 0.19 (0.17 to 0.21) |
| Male | 49.25(37.25-64.83) | 53.04(40.32-69.33) | 0.24 (0.21 to 0.26) |
| Population 70+ | 314.09(194.89-456.37) | 319.92(203.57-470.25) | 0.08 (0.04 to 0.11) |
| Population < 70 | 23.17(16.65-31.24) | 44.74(31.84-59.81) | 1.75 (1.53 to 1.97) |
| **Azerbaijan** |  |  |  |
| Absolute Numbers | 2079(1577-2727) | 4617(3523-6113) | 2.48 (2.35 to 2.62) |
| Age-Standardized Rates | 43.33(32.63-56.59) | 44.90(33.78-58.97) | 0.14 (0.12 to 0.16) |
| Female | 38.29(28.07-51.27) | 38.91(28.64-51.94) | 0.10 (0.07 to 0.12) |
| Male | 49.62(37.68-63.49) | 51.88(39.47-67.37) | 0.15 (0.14 to 0.17) |
| Population 70+ | 316.98(197.67-466.56) | 318.07(200.65-468.99) | 0.03 (0.01 to 0.05) |
| Population < 70 | 19.24(13.92-25.70) | 33.59(24.21-45.52) | 1.45 (1.10 to 1.80) |
| **Georgia** |  |  |  |
| Absolute Numbers | 2950(2187-3910) | 2830(2114-3690) | -0.37 (-0.44 to -0.3) |
| Age-Standardized Rates | 47.31(35.38-61.93) | 47.21(35.92-61.51) | -0.06 (-0.08 to -0.03) |
| Female | 41.68(30.70-55.32) | 40.98(30.21-53.86) | -0.08 (-0.11 to -0.05) |
| Male | 54.55(40.79-70.83) | 54.93(42.01-71.39) | -0.05 (-0.07 to -0.03) |
| Population 70+ | 338.46(213.87-495.39) | 328.27(216.72-474.48) | -0.15 (-0.16 to -0.13) |
| Population < 70 | 35.30(25.23-48.85) | 49.63(35.16-66.26) | 0.77 (0.58 to 0.96) |
| **Kazakhstan** |  |  |  |
| Absolute Numbers | 5935(4511-7796) | 8829(6626-11519) | 1.18 (1.04 to 1.33) |
| Age-Standardized Rates | 48.19(36.41-63.55) | 48.85(37.09-63.65) | 0.03 (0.00 to 0.06) |
| Female | 42.69(31.26-56.70) | 42.59(31.59-56.07) | -0.01 (-0.03 to 0.01) |
| Male | 55.37(42.13-72.39) | 56.79(43.24-73.83) | 0.05 (0.00 to 0.09) |
| Population 70+ | 346.11(214.99-517.23) | 341.05(214.59-498.31) | -0.08 (-0.13 to -0.03) |
| Population < 70 | 24.43(17.85-32.93) | 33.37(24.23-44.87) | 0.70 (0.54 to 0.85) |
| **Kyrgyzstan** |  |  |  |
| Absolute Numbers | 1203(909-1595) | 1957(1491-2572) | 1.29 (1.11 to 1.48) |
| Age-Standardized Rates | 41.46(31.00-54.33) | 41.13(31.43-53.83) | -0.06 (-0.07 to -0.05) |
| Female | 36.62(26.98-48.67) | 35.66(26.32-47.51) | -0.09 (-0.11 to -0.08) |
| Male | 47.59(35.88-61.72) | 47.85(37.03-61.36) | -0.05 (-0.06 to -0.03) |
| Population 70+ | 305.59(190.38-449.87) | 295.33(185.48-439.02) | -0.12 (-0.13 to -0.11) |
| Population < 70 | 17.61(12.83-23.70) | 20.84(15.26-27.94) | 0.25(-0.09 to 0.60) |
| **Tajikistan** |  |  |  |
| Absolute Numbers | 1084(831-1420) | 2333(1786-3072) | 2.42 (2.13 to 2.71) |
| Age-Standardized Rates | 41.09(31.09-53.75) | 40.85(30.53-53.30) | 0.01 (-0.01 to 0.03) |
| Female | 36.09(26.56-48.47) | 35.14(25.46-46.11) | -0.05 (-0.08 to -0.02) |
| Male | 46.64(35.43-60.44) | 46.80(35.25-61.31) | 0.03 (0.01 to 0.04) |
| Population 70+ | 304.74(191.19-448.18) | 297.04(184.21-438.44) | -0.04 (-0.06 to -0.02) |
| Population < 70 | 13.24(9.71-17.41) | 17.49(12.97-23.43) | 0.79 (0.39 to 1.19) |
| **Turkey** |  |  |  |
| Absolute Numbers | 10042(7802-12979) | 24774(21786-28278) | 2.73 (2.64 to 2.81) |
| Age-Standardized Rates | 32.02(24.36-41.70) | 27.35(24.06-31.31) | -0.78 (-0.89 to -0.67) |
| Female | 30.39(22.84-39.56) | 21.41(18.55-25.51) | -1.57 (-1.79 to -1.35) |
| Male | 34.30(26.15-44.80) | 34.59(30.48-38.96) | -0.11 (-0.18 to -0.04) |
| Population 70+ | 306.16(199.02-437.48) | 276.15(225.90-340.35) | -0.4 (-0.44 to -0.36) |
| Population < 70 | 9.74 (7.16-12.88) | 13.67(11.39-16.12) | 0.6 (0.27 to 0.94) |
| **Turkmenistan** |  |  |  |
| Absolute Numbers | 450(338-593) | 1872(1439-2433) | 2.76 (2.66 to 2.86) |
| Age-Standardized Rates | 43.24(32.26-56.89) | 47.04(35.47-60.99) | 0.28 (0.27 to 0.29) |
| Female | 38.02(27.74-50.75) | 40.77(29.56-53.36) | 0.25 (0.23 to 0.26) |
| Male | 49.51(37.38- 65.20) | 54.17(41.58-70.06) | 0.29 (0.26 to 0.32) |
| Population 70+ | 316.76(199.41-463.58) | 330.46(214.99-476.44) | 0.15 (0.13 to 0.18) |
| Population < 70 | 14.54(10.65-19.63) | 27.09(19.68-36.33) | 1.92 (1.69 to 2.16) |
| **Uzbekistan** |  |  |  |
| Absolute Numbers | 4414(3384-5822) | 10746(8148-14124) | 2.74 (2.61 to 2.87) |
| Age-Standardized Rates | 39.64 (29.94-52.67) | 41.51(30.94-54.26) | 0.1 (0.08 to 0.12) |
| Female Rates | 34.87(25.72-46.46) | 35.83(26.34-47.15) | 0.06 (0.05 to 0.07) |
| Male Rates | 45.37(34.94-59.61) | 48.07(36.23-62.32) | 0.11 (0.08 to 0.14) |
| Population 70+ Rates | 294.47(185.56-429.92) | 299.49(186.73-436.74) | 0.02 (0 to 0.04) |
| Population < 70 Rates | 13.41(9.82-17.89) | 23.71(17.26-31.87) | 1.62 (1.38 to 1.86) |

Table S8 Atrial fibrillation/flutter in EU-53: Prevalence by country, sex, and age

|  | **Prevalence** |  |  |
| --- | --- | --- | --- |
|  | **1990**  **n (95% UI)** | **2021**  **n (95% UI)** | **1990-2021EAPC**  **(95% CI)** |
| **WESTERN EUROPE** | | | |
| **Andorra** |  |  |  |
| Absolute Numbers | 462(354-595) | 1172(905.08- 1510.20) | 2.45 (2.16 to 2.73) |
| Age-Standardized Rates | 835.12(645.86-1082.98_ | 965.86(752.77-1219.43) | -0.53 (-0.58 to -0.47) |
| Female | 590.83(441.50-780.38) | 512.46(376.96-680.61) | -0.62 (-0.69 to -0.55) |
| Male | 1066.15(831.09-1364.47) | 743.99(574.87-956.34) | -0.43 (-0.48 to -0.39) |
| Population 70+ | 8548.11(6024.69-11487.90) | 8094.18(5811.10-10801.07) | -0.23 (-0.26 to -0.19) |
| Population < 70 | 368.41(277.49-495.14) | 546.09(408.92-726.03) | 1.09 (0.9 to 1.28) |
| **Austria** |  |  |  |
| Absolute Numbers | 80258(71857- 89871) | 241015(230229-251784) | 4.01 (3.86 to 4.16) |
| Age-Standardized Rates | 643.17(578.86-714.48) | 1217.22(1164.84-1272.17) | 2.33 (2.19 to 2.48) |
| Female | 468.81(420.94-535.13 | 845.47(802.57-889.96) | 2.16 (2.03 to 2.28) |
| Male | 913.86(814.35-1025.27) | 1663.69(1573.97-1746.73) | 2.19 (2.04 to 2.35) |
| Population 70+ | 7340.32(6389.78-8392.31) | 14722.72(13920.81-15516.02) | 2.65 (2.48 to 2.83) |
| Population < 70 | 346.79(305.35-400.79) | 747.89(701.04-800.35) | 2.87 (2.66 to 3.09) |
| **Belgium** |  |  |  |
| Absolute Numbers | 112962(86002-147149) | 163236(131345-207711) | 1.14 (1.05 to 1.22) |
| Age-Standardized Rates | 704.67(544.20-918.24) | 675.51(553.96-847.21) | -0.22 (-0.3 to -0.15) |
| Female | 537.93(393.11-716.96) | 486.44(386.14-627.67) | -0.44 (-0.53 to -0.36) |
| Male | 919.14(722.74-1184.92) | 885.66(724.83-1098.01) | -0.20 (-0.28 to -0.12) |
| Population 70+ | 8047.25(5788.51-10839.43) | 6958.40(5197.51-9091.27) | -0.54 (-0.71 to -0.38) |
| Population < 70 | 395.16(299.33 -518.63) | 520.75(399.06-679.17) | 0.28 (-0.02 to 0.6) |
| **Cyprus** |  |  |  |
| Absolute Numbers | 5114(3908-6514) | 10427(8434-13170) | 2.5 (2.25 to 2.76) |
| Age-Standardized Rates | 652.09(507.78-834.30) | 487.17(395.53-615.62) | -0.83 (-0.98 to -0.69) |
| Female | 481.41(360.40-634.90) | 328.87(259.99-428.50) | -1.12 (-1.26 to -0.98) |
| Male | 852.74(665.07-1073.81) | 669.35(544.58-819.44) | -0.64 (-0.80 to -0.48) |
| Population 70+ | 6175.47(4394.63-8323.49) | 5417.46(4194.31-7064.98) | -0.43 (-0.49 to -0.38) |
| Population < 70 | 265.60(197.33-347.90) | 252.98(195.85-325.38) | 0.28 (-0.02 to 0.6) |
| **Denmark** |  |  |  |
| Absolute Numbers | 69659(53710-89658) | 111718(88772-138463) | 1.09 (0.97 to 1.21) |
| Age-Standardized Rates | 817.12(637.85-1043.61) | 908.22(736.43-1119.51) | -0.06 (-0.24 to 0.13) |
| Female | 586.94(446.23-763.64) | 641.38(504.21-812.02) | 0.01 (-0.15 to 0.16) |
| Male | 1101.08(862.34-1400.13) | 1196.13(974.26-1467.98) | -0.23 (-0.43 to -0.02) |
| Population 70+ | 8617.70(6238.51-11525.79) | 8926.05(6639.50-11446.32) | -0.36 (-0.58 to -0.15) |
| Population < 70 | 468.65(353.94-614.78) | 700.76(557.75-883.21) | -0.02 (-0.25 to 0.21) |
| **Finland** |  |  |  |
| Absolute Numbers | 85278(64676-106114) | 117234(94965-141038) | 0.90 (0.78 to 1.02) |
| Age-Standardized Rates | 1168.45(891.81-1441.77) | 862.54(725.46-1013.76) | -1.12 (-1.23 to -1.01) |
| Female | 848.11(614.52-1079.84) | 607.90(485.41-743.75) | -1.24 (-1.35 to -1.13) |
| Male | 1606.77(1214.31-2003.51) | 1148.13(965.00-1325.33) | -0.48 (-0.53 to -0.42) |
| Population 70+ | 11608.60(7999.51-15198.63) | 9301.59(6947.33-11613.81) | -0.89 (-1.00 to -0.77) |
| Population < 70 | 706.97(523.91-869.02) | 654.27(586.44-719.61) | -0.02 (-0.25 to 0.21) |
| **France** |  |  |  |
| Absolute Numbers | 703229(534466-921039) | 1081926(820574-1416350) | 1.34 (1.27 to 1.42) |
| Age-Standardized Rates | 803.34(620.29-1041.29) | 717.98(554.42-925.96) | -0.50 (-0.57 to -0.44) |
| Female | 595.07(434.13-781.92) | 509.41(371.77-669.25) | -0.67 (-0.75 to -0.59) |
| Male | 1072.65(829.25-1369.19) | 962.24(752.03-1225.25) | -0.48 (-0.53 to -0.42) |
| Population 70+ | 9122.12(6353.69-12297.59) | 8036.38(5668.21-10775.29) | -0.35 (-0.44 to -0.26) |
| Population < 70 | 434.83(326.78-582.34) | 502.53(374.78-679.49) | 1.38 (0.95 to 1.81) |
| **Germany** |  |  |  |
| Absolute Numbers | 1225849(937078-1593976) | 2155152(1896612-2379617) | 1.87 (1.67 to 2.08) |
| Age-Standardized Rates | 927.92(719.81-1195.45) | 1072.86(943.99-1172.82) | 0.38 (0.16 to 0.6) |
| Female | 661.33(496.53-868.24) | 658.70(567.14-736.54) | 0.06 (-0.07 to 0.18) |
| Male | 1326.49(1020.36-1682.93) | 1545.60(1370.53-1707.52) | 0.28 (-0.02 to 0.57) |
| Population 70+ | 10072.79(7014.84-13359.55) | 10859.30(9315.19-12110.74) | 0.31 (0.17 to 0.44) |
| Population < 70 | 572.71(429.22-771.77) | 951.21(794.78-1083.76) | 1.38 (0.95 to 1.81) |
| **Greece** |  |  |  |
| Absolute Numbers | 105797(87036-133969) | 175318(132665-230098) | 1.57 (1.29 to 1.85) |
| Age-Standardized Rates | 686.94(566.89-865.16) | 662.08(510.11-855.56) | -0.32 (-0.58 to -0.06) |
| Female | 541.13(418.74-707.77) | 483.70(355.74-643.06) | -0.77 (-1.07 to -0.48) |
| Male | 855.52(732.83-1073.30) | 864.86(674.84-1119.26) | -0.01 (-0.25 to 0.23) |
| Population 70+ | 7183.94(5550.58-9267.97) | 7718.12(5470.81-10266.41) | 0.07 (-0.07 to 0.22) |
| Population < 70 | 399.72(328.30-510.80) | 493.82(366.61-664.63) | 0.29 (0.05 to 0.54) |
| **Iceland** |  |  |  |
| Absolute Numbers | 2087(1581-2704) | 4788(3892-5821) | 2.55 (2.50 to 2.60) |
| Age-Standardized Rates | 704.04(536.96-912.64) | 793.27(651.04-960.17) | 0.22 (0.14 to 0.30) |
| Female | 483.14(360.12-640.60) | 494.35(390.14-615.77) | -0.02 (-0.09 to 0.05) |
| Male | 962.19(740.22-1235.32) | 1110.34(924.22-1331.36) | 0.26 (0.16 to 0.35) |
| Population 70+ | 7868.14(5539.78-10502.66) | 8831.10(6843.45-10977.78) | 0.42 (0.33 to 0.51) |
| Population < 70 | 275.60(214.23-365.47) | 462.87(372.74-585.04) | 0.45 (0.32 to 0.58) |
| **Ireland** |  |  |  |
| Absolute Numbers | 31562(23747-40777) | 54616(42201-70424) | 1.34 (1.10 to 1.57) |
| Age-Standardized Rates | 757.71(578.57-979.99) | 666.48(516.95-858.36) | -0.86 (-0.98 to -0.73) |
| Female | 538.09(393.95-711.72) | 451.13(336.78-603.50) | -1.26 (-1.49 to -1.03) |
| Male | 1019.43(787.68-1298.62) | 900.37(705.11-1139.33) | -0.73 (-0.81 to -0.64) |
| Population 70+ | 7777.48(5397.48-10439.81) | 7122.40(5016.83-9562.00) | -0.82 (-0.98 to -0.67) |
| Population < 70 | 319.90(241.13-427.75) | 397.83(298.35-532.63) | 0.45 (0.32 to 0.58) |
| **Israel** |  |  |  |
| Absolute Numbers | 41616(31818-54188) | 147352(122034-167473) | 4.56 (4.26 to 4.86) |
| Age-Standardized Rates | 848.35(656.52-1096.61) | 1155.51(958.33-1312.39) | 1.46 (1.15 to 1.77) |
| Female | 619.03(460.14-817.93) | 908.92(672.73-1101.86) | 1.80 (1.40 to 2.21) |
| Male | 1117.64(868.65-1435.81) | 1432.22(1250.91-1584.28) | 1.17 (0.93 to 1.41) |
| Population 70+ | 9007.85(6314.31-12038.01) | 11933.69(9543.14-13800.93) | 1.45 (1.14 to 1.77) |
| Population < 70 | 305.58(230.41-405.81) | 556.83(445.98-648.74) | 0.25 (0.13 to 0.37) |
| **Italy** |  |  |  |
| Absolute Numbers | 824078(633970-1072624) | 1327156(1001901-1743600) | 1.22 (1.12 to 1.32) |
| Age-Standardized Rates | 897.86(697.07-1163.46) | 821.57(632.60-1065.60) | -0.60 (-0.69 to -0.51) |
| Female | 795.65(598.41-1048.76) | 692.90(518.77-919.53) | -0.57 (-0.64 to -0.49) |
| Male | 1010.78(790.35-1303.64) | 965.61(752.29-1233.40) | -0.63 (-0.82 to -0.43) |
| Population 70+ | 9835.05(6880.25-13203.40) | 9782.32(6918.22-12987.75) | -0.43 (-0.63 to -0.23) |
| Population < 70 | 544.88(402.45-729.65) | 605.32(451.34-815.67) | 0.25 (0.13 to 0.37) |
| **Luxembourg** |  |  |  |
| Absolute Numbers | 4288(3610-5095) | 8302(7313-9305) | 2.27 (2.13 to 2.40) |
| Age-Standardized Rates | 763.59(647.30-906.09) | 751.62(663.73-841.08) | 0.01 (-0.04 to 0.05) |
| Female | 585.59(477.85-724.14) | 518.55(466.53-571.89) | -0.32 (-0.37 to -0.27) |
| Male | 585.59(477.85-724.14) | 1011.66(867.70-1159.94) | 0.03 (0 to 0.07) |
| Population 70+ | 8596.68(6918.05-10456.93) | 8923.73(7533.63-10141.12) | 0.26 (0.20 to 0.32) |
| Population < 70 | 389.63(324.85-485.83) | 410.42(352.28-488.21) | 0.13 (-0.08 to 0.35) |
| **Malta** |  |  |  |
| Absolute Numbers | 2836(2179-3655) | 6323(5524-7076) | 2.97 (2.62 to 3.31) |
| Age-Standardized Rates | 676.78(521.13-871.70) | 598.49(529.84-673.49) | -0.06 (-0.35 to 0.23) |
| Female | 502.05(370.84-664.63) | 482.63(423.11-536.22) | 0.19 (-0.12 to 0.51) |
| Male | 894.14(700.66-1137.75) | 723.66(630.36-831.74) | -0.35 (-0.62 to -0.07) |
| Population 70+ | 7125.13(4942.08-9496.87) | 6712.53(5731.36-7626.36) | 0.19 (-0.10 to 0.47) |
| Population < 70 | 308.66(229.86-402.98) | 405.90(349.93-474.81) | 1.63 (1.32 to 1.94) |
| **Monaco** |  |  |  |
| Absolute Numbers | 602(451-792) | 754(573-971) | 0.52 (0.45 to 0.6) |
| Age-Standardized Rates | 787.04(609.64-1021.06) | 718.17(557.76-916.81) | -0.41 (-0.46 to -0.35) |
| Female | 580.54(429.23-772.32) | 503.80(372.78-667.12) | -0.59 (-0.66 to -0.52) |
| Male | 1045.61(814.05-1337.78) | 956.66(736.59-1218.16) | -0.38 (-0.43 to -0.33) |
| Population 70+ | 8879.54(6233.40-12046.04) | 7862.11(5603.23-10529.54) | -0.42 (-0.45 to -0.40) |
| Population < 70 | 636.42(472.87-840.24) | 632.14(462.15-844.52) | 0.07 (-0.05 to 0.2) |
| **Netherlands** |  |  |  |
| Absolute Numbers | 172361(156082-189738) | 287028(243038-332612) | 1.32 (1.17 to 1.47) |
| Age-Standardized Rates | 834.04(753.00-922.78) | 769.98(654.22-894.08) | -0.59 (-0.72 to -0.46) |
| Female | 687.86(617.97-766.74) | 618.94(496.94-736.62) | -0.71 (-0.86 to -0.57) |
| Male | 1009.36(907.36-1128.25) | 930.95(806.91-1069.62) | -0.57 (-0.69 to -0.44) |
| Population 70+ | 9233.35(8243.54-10372.18) | 8469.69(6960.69-10011.26) | -0.57 (-0.69 to -0.44) |
| Population < 70 | 395.29(328.98-472.58) | 535.53(423.86-663.92) | 0.96 (0.89 to 1.02) |
| **Norway** |  |  |  |
| Absolute Numbers | 57867(44003-75498) | 79412(61345-102809) | 0.90 (0.78 to 1.03) |
| Age-Standardized Rates | 798.36(620.77-1032.65) | 761.48(589.35-985.49) | -0.16 (-0.21 to -0.12) |
| Female | 566.96(423.63-752.71) | 498.96(376.30-660.13) | -0.48 (-0.53 to -0.43) |
| Male | 1085.63(849.82-1403.35) | 1041.98(812.70-1348.36) | -0.13 (-0.17 to -0.08) |
| Population 70+ | 8425.52(5960.94-11238.93) | 7864.63(5702.58-10492.26) | -0.22 (-0.29 to -0.15) |
| Population < 70 | 444.45(331.19-588.94) | 528.30(392.20-707.00) | 0.95 (0.78 to 1.12) |
| **Portugal** |  |  |  |
| Absolute Numbers | 105934(80401-138675) | 192005(161459-229225) | 2.04 (1.99 to 2.09) |
| Age-Standardized Rates | 758.21(585.05-989.75) | 695.71(591.04-820.14) | -0.27 (-0.31 to -0.23) |
| Female | 620.89(455.89-823.47) | 595.32(494.59-713.50) | -0.08 (-0.11 to -0.05) |
| Male | 927.58(726.63-1194.64) | 812.76(696.44-979.34) | -0.46 (-0.56 to -0.36) |
| Population 70+ | 7994.25(5607.54-10799.43) | 8490.42 (6941.18-10338.11) | 0.34 (0.22 to 0.47) |
| Population < 70 | 401.13(298.48-535.50) | 472.51(383.05-595.31) | 0.41 (0.26 to 0.56) |
| **San Marino** |  |  |  |
| Absolute Numbers | 292.98(225.43-381.67) | 583.14(441.90-760.84) | 2.10 (2.01 to 2.20) |
| Age-Standardized Rates | 800.69(623.05-1036.98) | 720.08(555.90-936.44) | -0.44 (-0.48 to -0.40) |
| Female | 581.79(430.98-764.36) | 504.68(377.58-665.64) | -0.59 (-0.64 to -0.53) |
| Male | 1052.24(812.50-1354.79) | 957.12(741.18-1229.34) | -0.39 (-0.42 to -0.35) |
| Population 70+ | 8588.31(6038.45-11478.67) | 8313.71(5907.86-10995.90) | -0.21 (-0.24 to -0.17) |
| Population < 70 | 431.36(324.33-571.46) | 544.85(403.63-731.19) | 0.68 (0.57 to 0.79) |
| **Spain** |  |  |  |
| Absolute Numbers | 489404(384802-625445) | 902888(806291-1004786) | 2.18 (1.98 to 2.39) |
| Age-Standardized Rates | 876.31(690.91-1108.62) | 863.92(780.25-960.23) | 0.05 (-0.09 to 0.19) |
| Female | 704.75(531.75-902.70) | 632.07(549.08-726.18) | -0.37 (-0.55 to -0.19) |
| Male | 1081.67(874.20-1353.71) | 1130.67(1028.68-1242.35) | 0.36 (0.20 to 0.52) |
| Population 70+ | 9391.37(6953.06-12180.39) | 9534.60(8317.43-10834.29) | 0.41 (0.26 to 0.55) |
| Population < 70 | 468.98(354.99-633.27) | 664.51(565.36-774.24) | 0.75 (0.64 to 0.86) |
| **Sweden** |  |  |  |
| Absolute Numbers | 163917(128606-209330) | 360875(270489- 465091) | 2.15 (2.00 to 2.31) |
| Age-Standardized Rates | 1010.87(810.08-1286.90) | 1529.82(1166.57-1943.26) | 1.05 (0.94 to 1.17) |
| Female | 831.28(637.15-1088.20) | 1211.57(898.26-1562.57) | 0.83 (0.62 to 1.05) |
| Male | 1210.78(975.95-1501.23) | 1852.00(1421.38-2340.23) | 1.15 (1.03 to 1.26) |
| Population 70+ | 11105.44(8083.20-14618.26) | 17451.32(12385.65-22812.76) | 0.99 (0.83 to 1.15) |
| Population < 70 | 569.57(439.52-754.64) | 940.43(698.32-1259.61) | 1.91 (1.65 to 2.17) |
| **Switzerland** |  |  |  |
| Absolute Numbers | 51170(39839-65184) | 84810(74812-97781) | 2.13 (1.68 to 2.58) |
| Age-Standardized Rates | 463.76(360.67-586.53) | 598.28(534.95-684.88) | 0.30 (-0.10 to 0.7) |
| Female | 335.89(257.60-434.65) | 295.83(252.29-357.20) | 0.17 (-0.25 to 0.60) |
| Male | 632.26(489.76-791.03) | 598.28(534.95-684.88) | 0.19 (-0.17 to 0.55) |
| Population 70+ | 5191.66(3896.94-6796.97) | 4943.79(4238.21-5857.10) | 0.30 (-0.07 to 0.66) |
| Population < 70 | 242.45(180.81-310.72) | 301.36(260.84-358.58) | 1.33 (0.91 to 1.76) |
| **United Kingdom** |  |  |  |
| Absolute Numbers | 589601(466035-750521) | 888645(725229-1093517) | 1.14 (0.98 to 1.30) |
| Age-Standardized Rates | 620.10(495.41-784.31) | 1309.75(1068.90-1611.71) | -0.07 (-0.2 to 0.05) |
| Female | 437.09(338.94-560.84) | 1015.51(800.44-1291.79) | -0.24 (-0.39 to -0.09) |
| Male | 859.65(695.06-1075.13) | 1614.93(1335.81-1959.20) | -0.13 (-0.24 to -0.02) |
| Population 70+ | 6598.80(4889.76-8577.79) | 6974.74(5415.18-8829.27) | 0.00 (-0.09 to 0.10) |
| Population < 70 | 350.43(270.08-460.41) | 426.80(342.16-543.5)9 | 0.56 (0.46 to 0.66) |
| **CENTRAL EUROPE** | | | |
| **Albania** |  |  |  |
| Absolute Numbers | 10877(8359-13976) | 26254(20002-34169) | 3.03 (2.97 to 3.1) |
| Age-Standardized Rates | 576.46(437.91-47.53) | 582.74(445.93-753.86) | 0.07 (0.04 to 0.10) |
| Female | 447.75(329.02-587.22) | 447.90(334.58-589.40) | 0.02 (0.00 to 0.05) |
| Male | 721.50(555.36-925.34) | 732.96(566.80-950.36) | 0.08 (0.05 to 0.11) |
| Population 70+ | 5658.42(3942.23-7722.28) | 5902.21(4219.98-8064.87) | 0.20 (0.17 to 0.23) |
| Population < 70 | 163.13(120.21-218.32) | 427.57(314.11-582.79) | 3.22 (3.19 to 3.25) |
| **Bosnia and Herzegovina** |  |  |  |
| Absolute Numbers | 20194(15626-26610) | 36920(28206-47883) | 2.07 (1.88 to 2.26) |
| Age-Standardized Rates | 551.75(424.34-722.20) | 574.34(442.67-741.06) | 0.17 (0.14 to 0.20) |
| Female | 436.38(326.80-580.48) | 446.89(332.53-588.65) | 0.10 (0.07 to 0.13) |
| Male | 706.35(545.16-922.20) | 731.85(565.88-938.40) | 0.16 (0.12 to 0.19) |
| Population 70+ | 5766.62(4094.89-7884.40) | 5835.48(4155.81-7935.46) | 0.27 (0.16 to 0.37) |
| Population < 70 | 243.05(178.53-325.17) | 488.76(359.54-672.90) | 1.79 (1.55 to 2.03) |
| **Bulgaria** |  |  |  |
| Absolute Numbers | 70573(53726-92308) | 86405(64938-113573) | 0.75 (0.71 to 0.78) |
| Age-Standardized Rates | 580.26(444.98-749.70) | 724.26(556.26-935.18) | -0.01 (-0.05 to 0.03) |
| Female | 453.28(338.64-596.57) | 441.76(326.39-583.02) | -0.01 (-0.04 to 0.03) |
| Male | 729.31(559.84-946.82) | 564.76(430.67-735.55) | 0.05 (0.01 to 0.09) |
| Population 70+ | 5843.83(4137.22-8002.44) | 5672.11(4023.94-7715.82) | 0.10 (0.02 to 0.18) |
| Population < 70 | 399.39(295.26-539.65) | 486.58(361.68-674.55) | 0.87 (0.73 to 1.00) |
| **Croatia** |  |  |  |
| Absolute Numbers | 22653(19259-27369) | 39620(35576-44450) | 2.29 (2.10 to 2.49) |
| Age-Standardized Rates | 388.27(328.33-464.05) | 420.02(381.35-470.13) | 0.69 (0.45 to 0.93) |
| Female | 305.79(242.69-392.13) | 263.07(243.37-288.99) | 0.05 (-0.24 to 0.35) |
| Male | 508.54(446.16-621.60) | 631.02(555.13-724.70) | 1.01 (0.84 to 1.19) |
| Population 70+ | 3763.51(2992.95-4745.85) | 4372.44(3833.47-4979.83) | 1.21 (0.88 to 1.54) |
| Population < 70 | 261.81(221.70-322.95 | 354.41(306.40-412.27) | 1.19 (0.96 to 1.43) |
| **Czech Republic** |  |  |  |
| Absolute Numbers | 93403(71391-120323) | 228258(188006-254737) | 3.21 (2.9 to 3.52) |
| Age-Standardized Rates | 665.74(509.98-854.56) | 981.52(815.26-1089.62) | 1.44 (1.06 to 1.81) |
| Female | 534.16(392.55-706.16) | 730.65(591.96-821.06) | 1.17 (0.83 to 1.50) |
| Male | 852.87(665.85-1106.41) | 1289.41(1062.80-1430.75) | 1.52 (1.11 to 1.94) |
| Population 70+ | 6971.12(4931.33-9597.97) | 10766.19(8537.76-12150.77) | 1.75 (1.29 to 2.21) |
| Population < 70 | 398.46(295.09-537.33) | 682.07(591.86-771.48) | 2.29 (2.08 to 2.5) |
| **Hungary** |  |  |  |
| Absolute Numbers | 101902(76999-132894) | 118850(90414-156204) | 0.5 (0.43 to 0.58) |
| Age-Standardized Rates | 681.32(519.83-880.64) | 574.65(443.45-748.72) | -0.60 (-0.66 to -0.55) |
| Female | 544.48(404.43-710.79) | 453.99(339.37-601.82) | -0.65 (-0.70 to -0.60) |
| Male | 875.80(675.29-1130.23) | 740.49(575.02-962.94) | -0.59 (-0.63 to -0.54) |
| Population 70+ | 7095.32(4994.62-9671.54) | 5848.07(4162.31-7913.89) | -0.56 (-0.63 to -0.49) |
| Population < 70 | 437.29(322.29-602.22) | 485.90(362.36-659.10) | 0.46 (0.3 to 0.61) |
| **Montenegro** |  |  |  |
| Absolute Numbers | 3723(2851-4875) | 5917(4506-7731) | 1.63 (1.50 to 1.76) |
| Age-Standardized Rates | 622.42(474.22-809.12) | 597.77(457.54-773.59) | -0.08 (-0.12 to -0.05) |
| Female | 492.22(363.99-651.82) | 466.44(347.54-611.36) | -0.16 (-0.19 to -0.12) |
| Male | 790.59(613.48-1028.28) | 760.12(585.66-984.08) | -0.08 (-0.12 to -0.05) |
| Population 70+ | 6511.56(4590.38-8803.70) | 5911.36(4220.75-7993.94) | -0.14 (-0.23 to -0.06) |
| Population < 70 | 275.90(200.26-374.45) | 442.53(328.09-599.74) | 1.31 (1.2 to 1.42) |
| **North Macedonia** |  |  |  |
| Absolute Numbers | 10256(7924-13365) | 18793(14283-24572) | 2.14 (2.08 to 2.20) |
| Age-Standardized Rates | 593.55(455.11-776.64) | 572.89(435.57-741.40) | -0.08 (-0.11 to -0.05) |
| Female | 461.14(342.30-612.20) | 441.64(326.18-583.30) | -0.11 (-0.14 to -0.08) |
| Male | 741.72(572.47-962.44) | 720.60(553.01-934.93) | -0.05 (-0.08 to -0.03) |
| Population 70+ | 6054.28(4292.49-8206.27) | 5535.64(3914.67-7625.05) | -0.1 (-0.21 to 0.00) |
| Population < 70 | 258.40(190.97-349.79) | 416.58(311.27-562.95) | 1.44 (1.34 to 1.54) |
| **Poland** |  |  |  |
| Absolute Numbers | 276686(211770-358761) | 648195(491254-846914) | 2.11 (1.85 to 2.37) |
| Age-Standardized Rates | 640.38(488.94-828.18) | 866.31(664.47-1117.94) | 0.25 (-0.03 to 0.53) |
| Female | 543.58(406.79-715.35) | 696.63(520.72-916.01) | 0.11 (-0.16 to 0.38) |
| Male | 776.23(606.58-1004.39) | 1086.53(845.81-1403.36) | 0.31 (0.00 to 0.62) |
| Population 70+ | 6624.98(4673.73-8955.58) | 8865.16(6358.07-12035.81) | 0.40 (0.16 to 0.65) |
| Population < 70 | 327.87(243.42-444.25) | 697.47(524.20-952.83) | 1.77 (1.50 to 2.05) |
| **Romania** |  |  |  |
| Absolute Numbers | 153776(118560-200226) | 162826(141283-191969) | 0.13 (-0.12 to 0.39) |
| Age-Standardized Rates | 568.65(435.33-734.23) | 412.59(359.76-488.00) | -1.17 (-1.44 to -0.90) |
| Female | 448.92(333.31-593.71) | 312.61(266.76-376.02) | -1.32 (-1.62 to -1.02) |
| Male | 718.94(555.21-932.00) | 546.00(476.58-639.52) | -1.01 (-1.25 to -0.77) |
| Population 70+ | 5854.31(4113.87-8017.14) | 4302.25(3611.68- 5162.08) | -0.96 (-1.26 to -0.67) |
| Population < 70 | 319.95(235.60-434.41) | 328.62(278.55-408.03) | -0.11 (-0.54 to 0.32) |
| **Serbia** |  |  |  |
| Absolute Numbers | 56918(44033-74679) | 70792(59259-87626) | 0.64 (0.4 to 0.88) |
| Age-Standardized Rates | 549.73(422.16-715.09) | 409.90(345.15-506.86) | -1.01 (-1.31 to -0.72) |
| Female | 434.87(324.26-572.84) | 310.16(257.65-390.76) | -1.16 (-1.48 to -0.84) |
| Male | 691.61(534.67-891.87) | 529.12(440.10-661.45) | -0.94 (-1.21 to -0.66) |
| Population 70+ | 5430.98(3890.07-7369.58) | 4323.84(3508.36-5419.84) | -0.64 (-0.94 to -0.34) |
| Population < 70 | 327.87(243.13-443.10) | 306.74(245.18-395.64) | -0.33 (-0.7 to 0.05) |
| **Slovakia** |  |  |  |
| Absolute Numbers | 50960(42632-58858) | 90634(74283-106522) | 1.75 (1.69 to 1.80) |
| Age-Standardized Rates | 848.64(713.21-976.42) | 917.33(758.01-1072.43) | 0.16 (0.08 to 0.24) |
| Female | 694.08(581.98-799.34) | 783.87(617.88-916.57) | 0.29 (0.22 to 0.37) |
| Male | 1050.93(865.23-1238.25) | 1073.00(865.61-1301.97) | -0.01 (-0.15 to 0.13) |
| Population 70+ | 8447.07(6906.24-9958.12) | 9641.23(7579.65-11484.02) | 0.42 (0.34 to 0.51) |
| Population < 70 | 465.12(370.92-562.84) | 676.64(547.85-832.87) | 1.41 (1.28 to 1.54) |
| **Slovenia** |  |  |  |
| Absolute Numbers | 15036(11602-19562) | 18968(16026- 21889) | 2.22 (2.17 to 2.27) |
| Age-Standardized Rates | 610.78(474.02-792.53) | 617.35(543.74-691.90) | 0.05 (0.01 to 0.09) |
| Female | 486.96(364.35-642.73) | 438.54(384.68-497.03) | -0.25 (-0.32 to -0.18) |
| Male | 793.83(612.04-1026.71) | 827.92(712.21-933.02) | 0.08 (0.03 to 0.12) |
| Population 70+ | 6480.70(4565.97-8862.77) | 6312.69(5333.53-7284.91) | 0.08 (-0.01 to 0.18) |
| Population < 70 | 335.45(248.63-457.60) | 544.12(461.09-625.16) | 1.46 (1.35 to 1.57) |
| **Belarus** |  |  |  |
| Absolute Numbers | 71527(55047-92982) | 101036(78621-132534) | 1.09 (1.04 to 1.14) |
| Age-Standardized Rates | 725.95(565.06-946.74) | 5689.44(4116.73-7581.30) | 0.34 (0.31 to 0.38) |
| Female Rates | 454.08(341.61-589.02) | 809.94(632.38-1059.69) | 0.30 (0.27 to 0.33) |
| Male | 556.58(429.87-719.16) | 499.46(376.65-658.17) | 0.36 (0.32 to 0.40) |
| Population 70+ | 5241.64(3782.69-7095.62) | 54286.57(39280.36-72338.34) | 0.44 (0.37 to 0.51) |
| Population < 70 | 359.64(265.33-477.09) | 558.50(414.14-750.20) | 1.17 (0.97 to 1.37) |
| **Estonia** |  |  |  |
| Absolute Numbers | 11240(8653-14638) | 17269(13159-22327) | 1.58 (1.51 to 1.65) |
| Age-Standardized Rates | 544.37(421.20-706.07) | 619.55(480.95-800.04) | 0.48 (0.44 to 0.53) |
| Female | 445.30(331.15-584.36) | 493.82(372.01-645.67) | 0.40 (0.36 to 0.45) |
| Male | 710.99(558.91-915.96) | 797.95(624.92-1033.39) | 0.44 (0.39 to 0.48) |
| Population 70+ | 5115.11(3625.49-6869.05) | 5800.07(4213.36-7720.22) | 0.56 (0.49 to 0.64) |
| Population < 70 | 356.63(265.73-477.38) | 551.26(412.80-733.89) | 1.30 (1.23 to 1.36) |
| **Latvia** |  |  |  |
| Absolute Numbers | 18682(14270- 24444) | 25774(22299-29649) | 1.46 (1.27 to 1.64) |
| Age-Standardized Rates | 516.84(396.14-673.25) | 629.57(547.41-715.02) | 0.97 (0.80 to 1.14) |
| Female | 414.19(309.92-547.80) | 470.49(410.04-539.15) | 0.80 (0.59 to 1.01) |
| Male | 691.39(539.83-906.00) | 871.95(741.26-988.10) | 1.02 (0.89 to 1.16) |
| Population 70+ | 4871.09(3482.53-6619.96) | 5771.48(4781.13-6791.30) | 1.01 (0.77 to 1.25) |
| Population < 70 | 349.95(257.38-473.24) | 599.41(501.54-700.77) | 1.87 (1.71 to 2.03) |
| **Lithuania** |  |  |  |
| Absolute Numbers | 25740(19772-33473) | 37996(28984-48993) | 1.43 (1.33 to 1.52) |
| Age-Standardized Rates | 567.12(437.53-735.84) | 634.20(495.02-808.33) | 0.42 (0.38 to 0.46) |
| Female | 459.65(341.10-608.49) | 508.45(385.83-658.65) | 0.39 (0.35 to 0.44) |
| Male | 730.92(569.70-953.13) | 820.95(643.04-1067.09) | 0.42 (0.37 to 0.46) |
| Population 70+ | 5429.28(3852.55-7399.65) | 5987.26(4380.22-8084.08) | 0.48 (0.40 to 0.55) |
| Population < 70 | 349.65(258.21-469.77) | 599.51(440.41-804.42) | 1.70 (1.65 to 1.76) |
| **Republic of Moldova** |  |  |  |
| Absolute Numbers | 23013(17873-30099) | 37430(28839-48105) | 1.62 (1.60 to 1.65) |
| Age-Standardized Rates | 551.37(427.29-709.11) | 622.47(480.68-796.46) | 0.45 (0.43 to 0.46) |
| Female | 443.56(333.98-574.76) | 495.11(372.63-646.39) | 0.40 (0.39 to 0.41) |
| Male | 710.04(551.59-924.75) | 801.88(626.44-1032) | 0.45 (0.43 to 0.46) |
| Population 70+ | 5044.30(3643.76-6907.35) | 5743.96(4168.67-7636.29) | 0.62 (0.56 to 0.68) |
| Population < 70 | 293.06(215.18-391.94) | 536.63(396.17-728.27) | 1.99 (1.82 to 2.16) |
| **Russian Federation** |  |  |  |
| Absolute Numbers | 1053310(820702-1363643) | 1639057(1272602-2138452) | 1.43 (1.35 to 1.51) |
| Age-Standardized Rates | 599.94(467.24-774.28) | 673.56(526.09-872.26) | 0.43 (0.33 to 0.52) |
| Female | 518.03(393.51-671.44) | 576.13(439.46-750.94) | 0.36 (0.29 to 0.43) |
| Male | 745.99(584.46-973.16) | 821.54(644.67-1070.98) | 0.41 (0.29 to 0.54) |
| Population 70+ | 5591.89(4024.75-7533.24) | 6259.27(4591.70-8387.14) | 0.53 (0.45 to 0.60) |
| Population < 70 | 363.24(268.93-487.77) | 569.55(420.52-769.09) | 1.31 (1.20 to 1.42) |
| **Ukraine** |  |  |  |
| Absolute Numbers | 397837(308635-521139) | 468750(362555-614414) | 0.55 (0.52 to 0.58) |
| Age-Standardized Rates | 559.63(438.88-727.55) | 586.74(457.73-760.23) | 0.19 (0.16 to 0.23) |
| Female | 475.79(361.95-621.61) | 494.78(379.31-651.95) | 0.20 (0.15 to 0.25) |
| Male | 701.83(551.39-920.64) | 727.08(568.76-943.79) | 0.12 (0.10 to 0.14) |
| Population 70+ | 5244.87(3774.08-7115.96) | 5504.64(3995.46-7410.78) | 0.35 (0.27 to 0.43) |
| Population < 70 | 385.18(284.04-523.23) | 524.35(386.36-706.09) | 0.77 (0.6 to 0.94) |
| **EASTERN EUROPE** | | | |
| **Armenia** |  |  |  |
| Absolute Numbers | 12536(9693-16435) | 24452(18789-32258) | 2.35 (2.22 to 2.48) |
| Age-Standardized Rates | 507.82(391.11-664.63) | 556.65(424.75-729.78) | 0.33 (0.31 to 0.35) |
| Female | 421.71(312.98-555.07) | 456.75(342.50-603.12) | 0.31 (0.28 to 0.33) |
| Male | 628.41(488.46-825.07) | 691.93(536.05-898.79) | 0.32 (0.29 to 0.34) |
| Population 70+ | 5266.39(3700.54-7112.14) | 5730.03(4123.49-7684.53) | 0.54 (0.37 to 0.71) |
| Population < 70 | 189.17(137.16-261.31) | 379.67 (279.81-514.25) | 1.78 (1.52 to 2.04) |
| **Azerbaijan** |  |  |  |
| Absolute Numbers | 22683(17563-29382) | 48767(38083-64570) | 2.50 (2.39 to 2.61) |
| Age-Standardized Rates | 501.70(385.38-655.28） | 540.88(415.38-701.51) | 0.29 (0.27 to 0.31) |
| Female | 421.77(311.31-558.17) | 442.37(331.72-591.08) | 0.22 (0.18 to 0.26) |
| Male | 627.91(491.29-812.85) | 670.38(523.22-868.76) | 0.23 (0.21 to 0.25) |
| Population 70+ | 5193.54(3620.01-7061.42) | 5406.85(3865.03-7357.80) | 0.27 (0.15 to 0.40) |
| Population < 70 | 155.12(114.42-210.91) | 277.25(201.82-377.44) | 1.41 (1.01 to 1.81) |
| **Georgia** |  |  |  |
| Absolute Numbers | 33800(25849-44479) | 35412(26991-46236) | 0.04 (-0.03 to 0.12) |
| Age-Standardized Rates | 702.97(541.52-925.85) | 568.73(436.23-738.62) | 0.00 (-0.02 to 0.03) |
| Female | 469.11(353.18-624.40) | 469.46(349.40-614.55) | -0.03 (-0.06 to 0.01) |
| Male | 558.89(425.91-728.40) | 713.70(549.44-933.61) | -0.03 (-0.06 to 0.00) |
| Population 70+ | 5683.54(4028.18-701.49) | 5863.93(4260.55-7855.54) | 0.15 (0.08 to 0.22) |
| Population < 70 | 289.59(209.33-398.31) | 418.60(307.02-569.94) | 0.78 (0.56 to 1.00) |
| **Kazakhstan** |  |  |  |
| Absolute Numbers | 65048(50303-84806) | 96397(74221-126067) | 1.29 (1.15 to 1.43) |
| Age-Standardized Rates | 562.81(428.43-731.60) | 589.25(453.42-768.59) | 0.16 (0.13 to 0.18) |
| Female | 476.53(354.10-624.85) | 489.68(365.53-646.04) | 0.11 (0.09 to 0.13) |
| Male | 710.08(552.39-915.04) | 742.16(580.07-961.28) | 0.13 (0.09 to 0.16) |
| Population 70+ | 5719.13(4025.92-7849.06) | 5743.92(4069.96-7793.00) | 0.15 (0.07 to 0.24) |
| Population < 70 | 194.61(141.26-260.22) | 170.59(125.61-227.67) | 0.72 (0.54 to 0.91) |
| **Kyrgyzstan** |  |  |  |
| Absolute Numbers | 13329(10299-17484) | 20779(16255-27052) | 1.24 (1.11 to 1.37) |
| Age-Standardized Rates | 478.91(366.47-626.57) | 491.16(377.25-639.22) | 0.05 (0.04 to 0.06) |
| Female | 403.00(301.41-533.89) | 403.57(302.97-532.68) | 0.01 (-0.02 to 0.04) |
| Male | 601.12(469.21-788.80) | 615.14(485.40-792.41) | 0.01 (0.00 to 0.03) |
| Population 70+ | 4953.60(3512.76-6681.58) | 4896.98(3505.56-6626.92) | 0.15 (0.05 to 0.26) |
| Population < 70 | 142.75(105.00-193.77) | 170.59(125.61-227.67) | 0.19 (-0.20 to 0.59) |
| **Tajikistan** |  |  |  |
| Absolute Numbers | 11955(9331-15431) | 23850(18509-31221) | 2.23 (1.98 to 2.49) |
| Age-Standardized Rates | 476.78(369.22-619.07) | 489.48(373.73-641.48) | 0.13 (0.10 to 0.15) |
| Female | 393.67(294.31-527.05) | 391.42(292.98-515.50) | 0.02 (-0.02 to 0.07) |
| Male | 585.38(459.13-752.40) | 594.20(462.63-772.47) | 0.07 (0.05 to 0.08) |
| Population 70+ | 4939.40(3495.39-6670.83) | 4848.29(3450.07 -6512.48) | 0.18 (0.07 to 0.29) |
| Population < 70 | 107.41(78.70- 143.99) | 142.60(104.84-191.96) | 0.71 (0.27 to 1.14) |
| **Turkey** |  |  |  |
| Absolute Numbers | 99437(77991-127594) | 247837(221168-275262) | 2.81 (2.69 to 2.92) |
| Age-Standardized Rates | 332.71(262.14-425.52) | 282.89(252.21-314.68) | -0.84 (-1.00 to -0.69) |
| Female | 315.49(244.78-406.23) | 215.44(189.27-250.85) | -1.73 (-2.01 to -1.45) |
| Male | 357.99(280.72-458.25) | 368.05(328.85-407.19) | -0.07 (-0.13 to -0.01) |
| Population 70+ | 3569.53(2581.42-4749.67) | 3139.81(2719.19-3634.59) | -0.49 (-0.64 to -0.35) |
| Population < 70 | 81.99(59.56-111.10) | 112.33(99.02- 132.91) | 0.53 (0.17 to 0.90) |
| **Turkmenistan** |  |  |  |
| Absolute Numbers | 8444(6460-11054) | 19896(15619-25583) | 2.87 (2.77 to 2.96) |
| Age-Standardized Rates | 501.72(380.44-654.49) | 563.75(432.79-729.21) | 0.40 (0.39 to 0.41) |
| Female | 417.37(307.95-550.58) | 462.20(344.12-605.84) | 0.36 (0.34 to 0.38) |
| Male | 625.73(482.69-823.69) | 699.67(545.11-902.34) | 0.37 (0.34 to 0.39) |
| Population 70+ | 4989.65(3511.18-6752.07) | 5665.13(4067.73-7594.91) | 0.55 (0.48 to 0.63) |
| Population < 70 | 116.72(85.93-158.19) | 220.37(162.53-301.12) | 1.89 (1.61 to 2.16) |
| **Uzbekistan** |  |  |  |
| Absolute Numbers | 49809(38683-65011) | 112675(87824-148398) | 2.59 (2.45 to 2.72) |
| Age-Standardized Rates | 460.42(354.54-596.53) | 496.56(380.60-651.61) | 0.21 (0.20 to 0.23) |
| Female Rates | 383.28(286.21-507.59) | 403.75(301.51-537.75) | 0.16 (0.15 to 0.17) |
| Male Rates | 571.26(448.90-738.18) | 615.13(476.82-803.69) | 0.17 (0.15 to 0.2) |
| Population 70+ Rates | 4855.74(3448.31-591.12) | 5013.09(3567.10-6749.36) | 0.14 (0.04 to 0.24) |
| Population < 70 Rates | 108.47(79.68-146.99) | 194.94(142.51-263.68) | 1.59 (1.32 to 1.86) |

Table S9 Atrial fibrillation/flutter in EU-53: Death by Country, sex, and age

|  | **Deaths** |  |  |
| --- | --- | --- | --- |
|  | **1990**  **n (95% UI)** | **2021**  **n (95% UI)** | **1990-2021EAPC**  **(95% CI)** |
| **WESTERN EUROPE** | | | |
| **Andorra** |  |  |  |
| Absolute Numbers | 2 (1-3) | 7 (5-9) | 4.14 (3.66 to 4.63) |
| Age-Standardized Rates | 4.80 (3.41-6.92) | 3.66 (2.64-4.82) | -0.59 (-0.77 to -0.40) |
| Female | 4.48 (2.91-7.13) | 3.63(2.50-4.91) | -0.36 (-0.53 to -0.19) |
| Male | 5.13 (3.61-7.48) | 3.63 (2.51-5.08) | -0.88 (-1.12 to -0.63) |
| Population 70+ | 52.55(36.91-77.01) | 70.18(50.71-91.96) | 1.45 (1.09 to 1.81) |
| Population < 70 | 0.45(0.31-0.63) | 0.50(0.32-0.69) | 0.61 (0.31 to 0.9) |
| **Austria** |  |  |  |
| Absolute Numbers | 659(592-698) | 1651(1349-1818) | 3.2 (2.95 to 3.45) |
| Age-Standardized Rates | 5.55(4.94-5.90) | 6.75 (5.58-7.41) | 0.78 (0.47 to 1.09) |
| Female | 5.55 (4.94-5.90) | 6.44(5.12-7.19) | 0.67 (0.37 to 0.97) |
| Male | 5.43 (4.77-5.83) | 7.11 (6.24-7.64) | 0.91 (0.57 to 1.26) |
| Population 70+ | 79.92(71.23-84.91) | 127.18(102.96-140.35) | 1.66 (1.36 to 1.95) |
| Population < 70 | 0.71 (0.67-0.74) | 0.89 (0.83-0.95) | 1.36 (1.10 to 1.62) |
| **Belgium** |  |  |  |
| Absolute Numbers | 694(605-747) | 1396(1088-1557) | 2.82 (2.59 to 3.05) |
| Age-Standardized Rates | 4.57(3.93-4.93) | 4.21(3.35-4.67) | 0.16 (-0.09 to 0.4) |
| Female | 4.46(3.72-4.87) | 3.98(3.00-4.52) | 0.09 (-0.17 to 0.35) |
| Male | 4.65 (4.23-4.95) | 4.54(3.89-4.93) | 0.25 (0.02 to 0.47) |
| Population 70+ | 66.83(57.71-72.27) | 83.15(64.14-93.10) | 1.36 (1.13 to 1.59) |
| Population < 70 | 0.57(0.55-0.61) | 0.60(0.56-0.64) | 0.32 (0.12 to 0.52) |
| **Cyprus** |  |  |  |
| Absolute Numbers | 58.81 (40.39-77.94) | 123.39(102.38-143.27) | 2.44 (2.26 to 2.61) |
| Age-Standardized Rates | 14.11(9.57-19.11) | 8.36 (6.79-9.85) | 0.16 (-0.09 to 0.40) |
| Female | 14.20(8.77-20.39) | 8.91(7.09-10.77) | -1.84 (-2.16 to -1.52) |
| Male | 14.22(9.98-19.22) | 6.55(5.00-8.06) | -2.07 (-2.37 to -1.78) |
| Population 70+ | 105.34(70.34-40.72) | 86.36(71.48-100.64) | -0.64 (-0.82 to -0.45) |
| Population < 70 | 0.62 (0.49 -0.78) | 0.53 (0.42-0.68) | -0.40 (-0.51 to -0.29) |
| **Denmark** |  |  |  |
| Absolute Numbers | 416(373-453) | 874(741-955) | 2.4 (1.98 to 2.82) |
| Age-Standardized Rates | 4.73(4.22-5.13) | 6.06(5.17-6.60) | 0.89 (0.44 to 1.34) |
| Female | 4.63(4.08-5.02) | 5.44(4.49-6.07) | 0.54 (0.04 to 1.03) |
| Male | 4.83(4.33-5.29) | 6.85(5.97-7.41) | 1.35 (0.94 to 1.76) |
| Population 70+ | 68.50(60.84-74.77) | 96.55(81.19-105.90) | 1.24 (0.57 to 1.92) |
| Population < 70 | 0.73(0.69-0.78) | 0.88(0.94-0.81) | 1.06 (0.72 to 1.40) |
| **Finland** |  |  |  |
| Absolute Numbers | 545(466-595) | 794(645-877) | 0.96 (0.71 to 1.21) |
| Age-Standardized Rates | 7.77(6.59-8.51) | 4.67(3.87-5.13) | -1.85 (-2.14 to -1.57) |
| Female | 7.79(6.47-8.62) | 4.21(3.36-4.72) | -2.23 (-2.56 to -1.9) |
| Male | 7.31(6.46-7.88) | 5.33(4.58-5.75) | -1.13 (-1.36 to -0.89) |
| Population 70+ | 110.50(93.96-120.78) | 79.98(64.26-88.62) | -1.21 (-1.54 to -0.89) |
| Population < 70 | 0.87(0.78-0.96) | 0.97(0.91-1.03) | 0.83 (0.63 to 1.04) |
| **France** |  |  |  |
| Absolute Numbers | 4923(4382-5224) | 9758(8012-10779) | 2.33 (2.16 to 2.5) |
| Age-Standardized Rates | 5.51(4.88-5.85) | 4.53(3.78-4.99) | -0.55 (-0.61 to -0.48) |
| Female | 5.39(4.67-5.79) | 4.35(3.51-4.89) | -0.59 (-0.66 to -0.52) |
| Male | 5.57(5.16-5.87) | 4.73(4.18-5.12) | -0.53 (-0.61 to -0.44) |
| Population 70+ | 88.82(78.53-94.40) | 94.60(77.16-104.80) | 0.47 (0.24 to 0.70) |
| Population < 70 | 0.57(0.54-0.61) | 0.64(0.59-0.70) | 0.66 (0.48 to 0.84) |
| **Germany** |  |  |  |
| Absolute Numbers | 8302(7144-9077) | 20539(16509- 22874) | 3.48 (3.29 to 3.67) |
| Age-Standardized Rates | 6.39(5.46-6.99) | 7.81(6.35-8.66) | 1.17 (0.96 to 1.37) |
| Female | 6.23 (5.17-6.90) | 7.52 (5.84-8.50) | 1.11 (0.91 to 1.30) |
| Male | 6.63 (6.01-7.06) | 8.13 (7.03-8.80) | 1.20 (0.97 to 1.43) |
| Population 70+ | 95.61(81.66-104.97) | 146.03(116.32-163.02) | 1.66 (1.48 to 1.85) |
| Population < 70 | 0.80(0.74-0.85) | 1.04(0.97-1.10) | 1.64 (1.05 to 2.23) |
| **Greece** |  |  |  |
| Absolute Numbers | 590(529-621) | 1527(1272-1673) | 3.02 (2.77 to 3.26) |
| Age-Standardized Rates | 4.51(4.00-4.77) | 4.25(3.60-4.62) | -0.30 (-0.36 to -0.24) |
| Female | 4.45(3.88-4.75) | 4.07(3.35-4.48) | -0.33 (-0.45 to -0.22) |
| Male | 4.56(4.15-4.84) | 4.58(4.02-4.95) | -0.08 (-0.17 to 0.00) |
| Population 70+ | 57.35(51.03-60.58) | 84.68(69.98-93.06) | 0.99 (0.63 to 1.36) |
| Population < 70 | 0.49(0.47-0.52) | 0.72(0.68-0.77) | 0.19 (-0.37 to 0.76) |
| **Iceland** |  |  |  |
| Absolute Numbers | 18(15-19) | 51(40-58) | 3.93 (3.74 to 4.13) |
| Age-Standardized Rates | 5.62(4.88-6.04) | 6.84(5.49-7.68) | 1.05 (0.86 to 1.23) |
| Female | 5.65(4.78-6.15) | 7.01(5.50-7.97) | 1.02 (0.85 to 1.19) |
| Male | 5.49(4.91-5.88) | 6.59(5.51-7.35) | 1.13 (0.87 to 1.39) |
| Population 70+ | 90.26(77.68-97.45) | 128.78(100.63-146.08) | 1.84 (1.58 to 2.11) |
| Population < 70 | 0.53 (0.50-0.57) | 0.71(0.64-0.80) | 1.23 (0.97 to 1.48) |
| **Ireland** |  |  |  |
| Absolute Numbers | 220(200-231) | 456(371-510) | 2.59 (2.33 to 2.85) |
| Age-Standardized Rates | 6.07(5.45-6.42) | 5.20(4.23-5.80) | -0.25 (-0.49 to 0.00) |
| Female | 5.75(5.08-6.14) | 5.06(3.97-5.78) | -0.09 (-0.35 to 0.17) |
| Male | 6.54(6.04-6.89) | 5.31(4.49-5.84) | -0.52 (-0.78 to -0.26) |
| Population 70+ | 73.82(66.91-77.87) | 83.57(67.28-93.73) | 0.70 (0.40 to 1.01) |
| Population < 70 | 0.64 (0.60-0.66) | 0.50(0.45-0.54) | -0.59 (-0.77 to -0.41) |
| **Israel** |  |  |  |
| Absolute Numbers | 256(228-273) | 714(580-794) | 3.46 (3.25 to 3.68) |
| Age-Standardized Rates | 6.31(5.50-6.76) | 4.84(3.98-5.35) | -0.66 (-0.85 to -0.48) |
| Female | 6.33(5.42-6.91) | 4.81(3.80-5.42) | -0.7 (-0.89 to -0.51) |
| Male | 6.28(5.63-6.71) | 4.80(4.08-5.24) | -0.68 (-0.87 to -0.49) |
| Population 70+ | 77.52(68.43-82.73) | 81.99(65.92-91.42) | 0.61 (0.32 to 0.90) |
| Population < 70 | 0.44(0.41-0.47) | 0.42(0.39-0.46) | 0.26 (0.01 to 0.51) |
| **Italy** |  |  |  |
| Absolute Numbers | 3323(2852-3560) | 9825(7692-11040) | 4.01 (3.8 to 4.21) |
| Age-Standardized Rates | 4.09(3.47-4.41) | 4.52(3.60-5.05) | 0.76 (0.54 to 0.97) |
| Female | 4.37(3.61-4.75) | 4.46(3.39-5.09) | 0.45 (0.22 to 0.68) |
| Male | 3.45(3.11-3.62) | 4.64(3.98-5.03) | 1.53 (1.32 to 1.75) |
| Population 70+ | 54.89(46.57-59.09) | 90.68(70.52-102.17) | 2.10 (1.85 to 2.35) |
| Population < 70 | 0.55(0.53 -0.57) | 0.58 (0.55-0.61) | 0.47 (0.28 to 0.65) |
| **Luxembourg** |  |  |  |
| Absolute Numbers | 33(31-35) | 83(70-92) | 3.38 (3.23 to 3.54) |
| Age-Standardized Rates | 6.64(6.11-7.02) | 6.32(5.38-7.03) | 0.24 (0.07 to 0.41) |
| Female | 6.49(5.88-6.93) | 6.10(5.00-6.87) | 0.17 (-0.02 to 0.35) |
| Male | 6.75 (6.30-7.10) | 6.68(5.91-7.34) | 0.49 (0.31 to 0.67) |
| Population 70+ | 88.01(81.50-93.06) | 118.58(99.44-131.85) | 1.31 (1.14 to 1.48) |
| Population < 70 | 0.81 (0.77-0.86) | 0.65(0.58-0.72) | -0.6 (-0.76 to -0.44) |
| **Malta** |  |  |  |
| Absolute Numbers | 20(17-20) | 58(47-66) | 3.86 (3.74 to 3.99) |
| Age-Standardized Rates | 5.28(4.67 -5.65) | 4.88(3.97-5.49) | -0.02 (-0.18 to 0.14) |
| Female | 5.72(4.94-6.18) | 5.49(4.33-6.29) | -0.03 (-0.2 to 0.14) |
| Male | 4.47(4.13-4.74) | 3.86(3.30-4.28) | -0.02 (-0.24 to 0.20) |
| Population 70+ | 68.49(60.81-72.81) | 77.36(62.06-87.37) | 0.71 (0.48 to 0.95) |
| Population < 70 | 0.55 (0.51-0.58) | 0.73(0.65-0.80) | 1.68 (1.40 to 1.96) |
| **Monaco** |  |  |  |
| Absolute Numbers | 4(3- 5) | 6(4-7) | 1.50 (1.20 to 1.80) |
| Age-Standardized Rates | 4.34(3.20-5.36) | 4.35(3.29-5.49) | 0.11 (-0.10 to 0.31) |
| Female | 4.02(2.81-5.06) | 4.15(2.88-5.58) | 0.24 (-0.02 to 0.5) |
| Male | 4.91(3.48-6.31) | 4.64(3.40-6.01) | -0.13 (-0.25 to -0.01) |
| Population 70+ | 71.34(52.96-87.78) | 79.84(59.49-101.23) | 0.67 (0.34 to 1.01) |
| Population < 70 | 0.83(0.60-1.05) | 0.72(0.53-0.99) | -0.12(-0.29 to 0.05) |
| **Netherlands** |  |  |  |
| Absolute Numbers | 1399(1213-1507) | 2501(2084-2738) | 1.67 (1.48 to 1.86) |
| Age-Standardized Rates | 6.99(6.03-7.56) | 6.01(5.03-6.57) | -0.72 (-0.86 to -0.59) |
| Female | 6.93(5.85-7.61) | 5.72(4.58-6.36) | -0.82 (-0.97 to -0.68) |
| Male | 6.92(6.23-7.36) | 6.48(5.75-6.99) | -0.51 (-0.64 to -0.39) |
| Population 70+ | 102.88(88.83-111.28) | 97.35 (80.42-106.81) | -0.25 (-0.35 to -0.14) |
| Population < 70 | 0.58(0.55-0.60) | 0.75(0.70-0.80) | 1.12 (0.97 to 1.26) |
| **Norway** |  |  |  |
| Absolute Numbers | 491(431-522) | 778(629-857) | 1.41 (1.14 to 1.68) |
| Age-Standardized Rates | 6.27(5.51-6.68) | 5.94(4.86-6.51) | -0.25 (-0.51 to 0.01) |
| Female | 5.77(4.92-6.22) | 5.61(4.45-6.24) | -0.08 (-0.33 to 0.17) |
| Male | 7.01(6.42-7.34) | 6.35(5.49-6.86) | -0.51 (-0.82 to -0.21) |
| Population 70+ | 93.67(81.76-99.99) | 108.12(86.69-119.40) | 0.73 (0.28 to 1.18) |
| Population < 70 | 0.88(0.84-0.90) | 0.62(0.58-0.65) | -0.69 (-1.08 to -0.30) |
| **Portugal** |  |  |  |
| Absolute Numbers | 507(462-531) | 1173(969-1288) | 2.77 (2.63 to 2.91) |
| Age-Standardized Rates | 4.69(4.21-4.93) | 3.46(2.90-3.78) | -1.17 (-1.36 to -0.99) |
| Female | 4.62(4.09-4.91) | 3.18(2.55-3.55) | -1.38 (-1.59 to -1.17) |
| Male | 4.70(4.36-4.94) | 3.89(3.41-4.21) | -0.83 (-1.00 to -0.66) |
| Population 70+ | 53.68(48.58-56.44) | 63.43(51.88-69.92) | 0.53 (0.37 to 0.68) |
| Population < 70 | 0.49(0.47-0.52) | 0.57(0.53-0.61) | 0.48 (0.30 to 0.67) |
| **San Marino** |  |  |  |
| Absolute Numbers | 2(2-3) | 4(2-5) | 2.49 (2.01 to 2.97) |
| Age-Standardized Rates | 5.93(4.79-7.00) | 3.03(2.03-4.31) | -1.30 (-1.69 to -0.91) |
| Female | 5.99(4.51-7.39) | 3.40(2.09-5.02) | -1.46 (-1.86 to -1.06) |
| Male | 5.76(4.64-6.98) | 2.56(1.74-3.77) | -1.08 (-1.64 to -0.52) |
| Population 70+ | 91.48(73.73-108.40) | 69.22(45.88-99.49) | 0.02 (-0.42 to 0.47) |
| Population < 70 | 0.54(0.43-0.66) | 0.42(0.24-0.65) | 0.08 (-0.24 to 0.40) |
| **Spain** |  |  |  |
| Absolute Numbers | 2611(2311-2779) | 6767(5396-7550) | 3.25 (3.13 to 3.37) |
| Age-Standardized Rates | 5.21(4.56-5.56) | 4.54(3.69-5.04) | -0.35 (-0.44 to -0.26) |
| Female | 5.28(4.52-5.69) | 4.43(3.42-5.05) | -0.57 (-0.67 to -0.47) |
| Male | 4.94(4.49-5.22) | 4.67(4.11-5.04) | 0.12 (-0.01 to 0.24) |
| Population 70+ | 70.12(61.51-74.80) | 96.96(76.77-108.60) | 1.21 (1.03 to 1.40) |
| Population < 70 | 0.55(0.52-0.58) | 0.53(0.49-0.57) | -0.49 (-0.67 to -0.31) |
| **Sweden** |  |  |  |
| Absolute Numbers | 839(737-898) | 2851(2296-3193) | 4.44 (4.09 to 4.78) |
| Age-Standardized Rates | 4.90(4.29-5.25) | 9.47(7.71-10.59) | 2.57 (2.28 to 2.87) |
| Female | 4.77(4.07-5.17) | 8.83(6.76-10.09) | 2.43 (2.16 to 2.70) |
| Male | 4.97(4.54-5.22) | 10.29 (8.70-11.70) | 2.79 (2.42 to 3.15) |
| Population 70+ | 73.31(64.03-78.55) | 174.79(140.14-196.04) | 3.42 (2.90 to 3.95) |
| Population < 70 | 0.52(0.50-0.55) | 0.72(0.63-0.81) | 1.98 (1.59 to 2.38) |
| **Switzerland** |  |  |  |
| Absolute Numbers | 317(271-346) | 773 (609-867) | 3.15 (3.00 to 3.31) |
| Age-Standardized Rates | 2.74 (2.35-3.01) | 3.00 (2.39-3.34) | 0.62 (0.50 to 0.75) |
| Female | 2.59 (2.13-2.89) | 2.81(2.11-3.21) | 0.61 (0.47 to 0.75) |
| Male | 3.00 (2.69-3.26) | 3.25 (2.78-3.54) | 0.61 (0.5 to 0.71) |
| Population 70+ | 42.72(36.29-46.85) | 59.96 (46.88-67.38) | 1.36 (1.24 to 1.49) |
| Population < 70 | 0.31 (0.28-0.33) | 0.33 (0.30-0.35) | 0.61 (0.41 to 0.81) |
| **United Kingdom** |  |  |  |
| Absolute Numbers | 5083(4544-5338) | 9412(7868-10176) | 2.26 (2.14 to 2.38) |
| Age-Standardized Rates | 5.70(5.30-5.90) | 5.71(4.80-6.15) | 0.30 (0.17 to 0.43) |
| Female | 5.27(4.56-5.61) | 5.60(4.56-6.13) | 0.37 (0.24 to 0.51) |
| Male | 5.47(4.84-5.77) | 5.75(5.11-6.08) | 0.17 (0.04 to 0.31) |
| Population 70+ | 76.15(67.60-80.22) | 98.31(81.65-106.58) | 1.16 (1.06 to 1.25) |
| Population < 70 | 0.67(0.66-0.69) | 0.71(0.68-0.73) | 0.48 (0.34 to 0.63) |
| **CENTRAL EUROPE** | | | |
| **Albania** |  |  |  |
| Absolute Numbers | 59(51-69) | 181(132-229) | 4.29 (4.08 to 4.51) |
| Age-Standardized Rates | 4.02(3.41-4.65) | 4.72(3.41-6.04) | 0.92 (0.73 to 1.10) |
| Female | 3.76(3.06-4.44) | 4.94(3.39-6.18) | 1.44 (1.20 to 1.67) |
| Male | 5.04(3.67-6.38) | 4.47(2.92-6.18) | -0.46 (-0.61 to -0.31) |
| Population 70+ | 52.31(44.49-61.02) | 61.60(44.47-78.73) | 0.95 (0.72 to 1.18) |
| Population < 70 | 0.22(0.19-0.26) | 0.58 (0.34-0.96) | 3.29 (3.06 to 3.52) |
| **Bosnia and Herzegovina** |  |  |  |
| Absolute Numbers | 91.17(72-110) | 258(207-309) | 3.66 (3.48 to 3.83) |
| Age-Standardized Rates | 3.21(2.53-3.90) | 4.07(3.26-4.87) | 0.91 (0.80 to 1.01) |
| Female | 2.91(2.31-3.79) | 3.85(2.93-4.68) | 1.13 (0.92 to 1.34) |
| Male | 3.74 (2.67-4.70) | 4.40(3.28-5.62) | 0.61 (0.40 to 0.83) |
| Population 70+ | 42.48(33.30-51.74) | 59.82(47.69-72.22) | 1.25 (0.97 to 1.53) |
| Population < 70 | 0.46(0.37-0.58) | 0.89(0.65-1.16) | 1.52 (1.25 to 1.79) |
| **Bulgaria** |  |  |  |
| Absolute Numbers | 393(346-449) | 902(786-1026) | 2.72 (2.55 to 2.88) |
| Age-Standardized Rates | 5.51(4.87-6.23) | 6.17(5.40-6.98) | 0.50 (0.34 to 0.67) |
| Female | 5.27(4.57-5.93) | 5.09(4.44-5.78) | -0.06 (-0.24 to 0.12) |
| Male | 5.76(4.65-7.13) | 7.97(6.95-9.04) | 1.28 (1.12 to 1.44) |
| Population 70+ | 46.11(40.81-52.18) | 77.41(67.66-87.57) | 1.91 (1.73 to 2.09) |
| Population < 70 | 1.11(0.95-1.31) | 1.83(1.52-2.19) | 1.63 (1.39 to 1.87) |
| **Croatia** |  |  |  |
| Absolute Numbers | 142(132-150) | 363(319-401) | 3.49 (2.97 to 4.02) |
| Age-Standardized Rates | 3.02(2.79-3.19) | 3.51(3.09-3.87) | 0.62 (0.2 to 1.04) |
| Female | 2.65(2.42-2.81) | 3.35(2.90-3.71) | 0.77 (0.25 to 1.29) |
| Male | 3.88(3.62-4.13) | 3.77(3.34-4.17) | 0.25 (0.07 to 0.43) |
| Population 70+ | 44.33(41.13-46.99) | 55.81(48.76-61.60) | 1.32 (0.47 to 2.18) |
| Population < 70 | 0.35(0.33-0.37) | 0.55(0.48-0.63) | 1.53 (1.32 to 1.75) |
| **Czech Republic** |  |  |  |
| Absolute Numbers | 505(473-532) | 1123(973-1237) | 3.02 (2.86 to 3.17) |
| Age-Standardized Rates | 4.08(3.79-4.30) | 4.68(4.06-5.15) | 0.68 (0.57 to 0.79) |
| Female | 3.77(3.45-3.98) | 4.25(3.66-4.70) | 0.56 (0.45 to 0.66) |
| Male | 4.77 (4.47-5.06) | 5.37(4.71-5.90) | 0.76 (0.53 to 0.98) |
| Population 70+ | 55.63(51.88-58.65) | 67.95 (58.47-74.89) | 1.20 (1.06 to 1.33) |
| Population < 70 | 0.65(0.60-0.70) | 0.80(0.70-0.93) | 1.56 (1.24 to 1.87) |
| **Hungary** |  |  |  |
| Absolute Numbers | 528(494-563) | 796(678-894) | 1.74 (1.62 to 1.86) |
| Age-Standardized Rates | 4.34(4.02-4.63) | 3.55(3.03-3.99) | -0.38 (-0.52 to -0.25) |
| Female | 4.14(3.81-4.43) | 3.40 (2.86-3.85) | -0.4 (-0.51 to -0.28) |
| Male | 4.76(4.47-5.06) | 3.80(3.30-4.23) | -0.37 (-0.55 to -0.19) |
| Population 70+ | 54.30(50.57-57.88) | 53.82(45.51-60.44) | 0.51 (0.36 to 0.65) |
| Population < 70 | 0.71(0.67-0.76) | 0.87(0.73-0.99) | 1.00 (0.83 to 1.17) |
| **Montenegro** |  |  |  |
| Absolute Numbers | 61(52-78) | 133.18(107-163) | 3.02 (2.73 to 3.31) |
| Age-Standardized Rates | 11.32(9.48-14.40) | 17.26(13.44-21.44) | 1.70 (1.23 to 2.18) |
| Female | 8.14(6.63-10.19) | 14.07(9.97-17.58) | 2.29 (1.86 to 2.71) |
| Male | 16.62(13.38-23.08) | 22.57(16.83-30.99) | 0.98 (0.43 to 1.53) |
| Population 70+ | 163.42(136.02-209.03) | 203.46(160.50-253.40) | 1.10 (0.53 to 1.67) |
| Population < 70 | 1.54(1.30-1.85) | 2.65(2.02-3.29) | 1.60 (1.38 to 1.83) |
| **North Macedonia** |  |  |  |
| Absolute Numbers | 64(54-77) | 149(110-214) | 2.88 (2.76 to 2.99) |
| Age-Standardized Rates | 4.41(3.68-5.34) | 7.45(5.80-9.50) | 1.47 (0.87 to 2.08) |
| Female | 3.91(3.17-4.95) | 7.96 (5.87-11.21) | 1.90 (1.32 to 2.49) |
| Male | 5.27(4.14-6.62) | 6.48(4.46-8.37) | 0.89 (0.24 to 1.55) |
| Population 70+ | 63.61(52.86-77.05) | 69.58(52.52-97.67) | 0.29 (0.05 to 0.53) |
| Population < 70 | 0.43(0.36-0.52) | 0.86(0.53-1.38) | 1.94 (1.75 to 2.13) |
| **Poland** |  |  |  |
| Absolute Numbers | 2503(2303-2671) | 4021(3480-4421) | 2.12 (1.69 to 2.55) |
| Age-Standardized Rates | 6.80(6.18-7.24) | 4.96(4.31-5.45) | -0.67 (-1.18 to -0.16) |
| Female | 6.68(5.89-7.36) | 4.83(4.07-5.36) | -0.70 (-1.21 to -0.19) |
| Male | 7.07(6.48-7.67) | 5.20(4.63-5.67) | -0.63 (-1.15 to -0.12) |
| Population 70+ | 89.46(81.69-95.42) | 79.22(67.94-87.16) | 0.28 (-0.13 to 0.69) |
| Population < 70 | 0.98(0.93-1.04) | 0.96 (0.87-1.05) | 0.52 (0.10 to 0.95) |
| **Romania** |  |  |  |
| Absolute Numbers | 674(625-726) | 1429(1263-1586) | 2.24 (2.01 to 2.47) |
| Age-Standardized Rates | 3.58(3.33-3.83) | 3.35(2.97-3.72) | -0.56 (-0.74 to -0.39) |
| Female | 3.56(3.22-3.88) | 3.18(2.78-3.53) | -0.67 (-0.84 to -0.51) |
| Male | 3.60(3.19-4.08) | 3.63(3.24-4.05) | -0.38 (-0.57 to -0.19) |
| Population 70+ | 40.14(37.34-43.03) | 51.69(45.31-57.39) | 0.63 (0.23 to 1.03) |
| Population < 70 | 0.46(0.41-0.52) | 0.73(0.64-0.84) | 1.00 (0.72 to 1.28) |
| **Serbia** |  |  |  |
| Absolute Numbers | 375(318-435) | 810(665-962) | 2.59 (2.3 to 2.88) |
| Age-Standardized Rates | 6.22(5.28-7.24) | 4.63(3.83-5.50) | -1.49 (-1.84 to -1.13) |
| Female | 5.50(4.58-6.68) | 4.22(3.34-5.05) | -1.67 (-2.06 to -1.27) |
| Male | 7.49 (6.12-9.00) | 5.37(4.23-6.80) | -1.33 (-1.66 to -1.00) |
| Population 70+ | 75.44(63.97-87.57) | 69.10(56.63-82.13) | 0.42 (-0.12 to 0.97) |
| Population < 70 | 0.57(0.48-0.68) | 0.81(0.60-0.99) | 0.81 (0.62 to 1.00) |
| **Slovakia** |  |  |  |
| Absolute Numbers | 305(263-366) | 562(463-658) | 2.28 (2.18 to 2.37) |
| Age-Standardized Rates | 5.75(4.94-6.89) | 5.99(4.90-7.02) | 0.39 (0.28 to 0.50) |
| Female | 5.70(4.68-7.04) | 5.86(4.62-7.19) | 0.25 (0.16 to 0.35) |
| Male | 5.79(4.88-7.20) | 6.10(4.86-7.26) | 0.63 (0.48 to 0.78) |
| Population 70+ | 79.14(68.18-95.09) | 83.80(68.51-98.40) | 0.79 (0.61 to 0.96) |
| Population < 70 | 0.87(0.74-1.06) | 1.21(0.90-1.55) | 1.45 (1.30 to 1.61) |
| **Slovenia** |  |  |  |
| Absolute Numbers | 83(76-89) | 214(175-242) | 3.55 (3.26 to 3.83) |
| Age-Standardized Rates | 3.54(3.22-3.83) | 3.67(3.01-4.15) | 0.46 (0.03 to 0.88) |
| Female | 3.54(3.16-3.91) | 3.30(2.65-3.81) | 0.13 (-0.26 to 0.53) |
| Male | 3.51(3.22-3.76) | 4.48(3.90-5.01) | 1.14 (0.66 to 1.63) |
| Population 70+ | 53.36(48.54-57.61) | 68.00(55.70-77.16) | 1.18 (0.98 to 1.38) |
| Population < 70 | 0.53(0.50-0.57) | 0.54(0.45-0.63) | -0.06(-0.46 to 0.34) |
| **EASTERN EUROPE** | | | |
| **Belarus** |  |  |  |
| Absolute Numbers | 415(354-477) | 677(566-781) | 1.56 (1.43 to 1.69) |
| Age-Standardized Rates | 3.64(3.08-4.18) | 4.06(3.41-4.69) | 0.17 (0.09 to 0.25) |
| Female | 3.70(3.24-4.48) | 3.90(3.24-4.54) | -0.07(-0.17 to 0.02) |
| Male | 3.47(2.61-4.59) | 4.55(3.92 -5.17( | 0.89 (0.80 to 0.97) |
| Population 70+ | 52.54 (44.52-60.71) | 63.62(53.34-73.20) | 0.66 (0.27 to 1.05) |
| Population < 70 | 0.51(0.45-0.58) | 0.83(0.67-1.00) | 0.89 (0.63 to 1.16) |
| **Estonia** |  |  |  |
| Absolute Numbers | 70(64-77) | 190(162-212) | 3.21 (2.87 to 3.55) |
| Age-Standardized Rates | 3.95(3.57-4.34) | 5.33(4.54-5.96) | 0.82 (0.71 to 0.93) |
| Female | 3.78(3.42-4.14) | 4.70(3.95-5.30) | 0.52 (0.44 to 0.61) |
| Male | 4.56(4.04-5.11) | 7.07(6.17-7.89) | 1.35 (1.16 to 1.53) |
| Population 70+ | 52.41(47.67-57.19) | 94.91(80.49-106.19) | 1.72 (1.32 to 2.13) |
| Population < 70 | 0.56(0.49-0.66) | 0.77(0.66-0.87) | 0.54 (0.31 to 0.78) |
| **Latvia** |  |  |  |
| Absolute Numbers | 122(112-129) | 226(194-250) | 2.33 (1.99 to 2.67) |
| Age-Standardized Rates | 3.74(3.44-3.97) | 4.49(3.88-4.98) | 0.80 (0.59 to 1.02) |
| Female | 3.67(3.30-3.94) | 4.18(3.54-4.67) | 0.62 (0.42 to 0.82) |
| Male | 3.97(3.62-4.35) | 5.33(4.71-5.94) | 1.26 (0.99 to 1.53) |
| Population 70+ | 52.33(47.98-55.50) | 75.04(64.66-83.24) | 1.30 (0.87 to 1.74) |
| Population < 70 | 0.53(0.49-0.59) | 0.90(0.79-1.01) | 1.45 (1.24 to 1.66) |
| **Lithuania** |  |  |  |
| Absolute Numbers | 154(138-170) | 328(288-363) | 2.55 (2.36 to 2.75) |
| Age-Standardized Rates | 3.67(3.30-4.04) | 4.52(3.98-5.01) | 0.73 (0.62 to 0.84) |
| Female | 3.64(3.29-3.97) | 4.19(3.64-4.68) | 0.51 (0.41 to 0.61) |
| Male | 3.71(3.26-4.24) | 5.36(4.81-5.88) | 1.34 (1.17 to 1.50) |
| Population 70+ | 54.26(49.00-59.60) | 76.30(66.58-84.43) | 0.98 (0.62 to 1.35) |
| Population < 70 | 0.47(0.41-0.55) | 0.93(0.82-1.06) | 1.96 (1.76 to 2.16) |
| **Republic of Moldova** |  |  |  |
| Absolute Numbers | 108(99-116) | 215(189-238) | 2.26 (2.09 to 2.44) |
| Age-Standardized Rates | 3.96(3.65-4.24) | 3.50(3.09-3.88) | -0.63 (-0.80 to -0.46) |
| Female | 3.88(3.56-4.14) | 3.32(2.88-3.73) | -0.68 (-0.82 to -0.54) |
| Male | 4.17(3.74-4.62) | 3.88(3.43-4.28) | -0.61 (-0.84 to -0.38) |
| Population 70+ | 42.18(38.80-45.45) | 53.17(46.50-58.94) | 0.90 (0.64 to 1.16) |
| Population < 70 | 0.45 (0.41-0.50) | 0.92(0.82 -1.02) | 2.17 (1.93 to 2.41) |
| **Russian Federation** |  |  |  |
| Absolute Numbers | 5109(4515-5816) | 10825(9597-11704) | 2.47 (2.3 to 2.65) |
| Age-Standardized Rates | 3.92(3.42-4.52) | 4.44(3.92-4.80) | 0.38 (0.25 to 0.51) |
| Female | 3.85(3.36-4.43) | 4.43(3.87-4.85) | 0.40 (0.27 to 0.53) |
| Male | 4.26(3.68-4.86) | 4.35(3.86-4.77) | 0.10 (-0.03 to 0.24) |
| Population 70+ | 45.70(40.54-51.84) | 67.28(59.04-72.85) | 1.39 (1.01 to 1.77) |
| Population < 70 | 0.49(0.43-0.57) | 0.92(0.84-1.00) | 1.46 (1.18 to 1.75) |
| **Ukraine** |  |  |  |
| Absolute Numbers | 2156(1971- 2362) | 3236(2489-4051) | 1.11 (0.92 to 1.31) |
| Age-Standardized Rates | 3.85(3.47-4.24) | 4.10(3.16-5.13) | -0.21 (-0.44 to 0.03) |
| Female | 3.96(3.38-4.54) | 4.05(2.91-5.40) | -0.34 (-0.57 to -0.11) |
| Male | 3.54(3.06-4.11) | 4.27(3.29-5.37) | 0.20(-0.06 to 0.45) |
| Population 70+ | 47.38(42.99-52.11) | 60.24(46.74-75.44) | 0.59 (0.23 to 0.94) |
| Population < 70 | 0.53(0.49-0.58) | 0.78(0.58-1.01) | 0.73 (0.43 to 1.02) |
| **NO DEFINED SUBREGION** | | | |
| **Armenia** |  |  |  |
| Absolute Numbers | 31(27-35) | 108(93-122) | 4.92 (4.39 to 5.46) |
| Age-Standardized Rates | 1.52(1.31-1.77) | 2.53 (2.18-2.86) | 2.04 (1.67 to 2.40) |
| Female | 1.52(1.31-1.79) | 2.42 (2.07 -2.71) | 1.88 (1.54 to 2.21) |
| Male | 1.51(1.25-1.81) | 2.70 (2.34-3.06) | 2.31 (1.84 to 2.78) |
| Population 70+ | 21.32 (18.34-25.00) | 39.12(33.63-44.10) | 0.95 (0.72 to 1.18) |
| Population < 70 | 0.16 (0.15-0.18) | 0.45 (0.38 -0.51) | 3.10 (2.50 to 3.70) |
| **Azerbaijan** |  |  |  |
| Absolute Numbers | 59(48-78) | 167(121-214) | 2.77 (2.56 to 2.97) |
| Age-Standardized Rates | 1.65(1.34-2.14) | 1.79(1.39-2.21) | 0.76 (0.51 to 1.00) |
| Female | 1.46(1.13-2.05) | 1.70(1.30-2.13) | 1.09 (0.84 to 1.33) |
| Male | 2.38(1.90-2.94) | 2.02(1.54-2.61) | -0.32 (-0.62 to -0.02) |
| Population 70+ | 20.69(16.32-28.39) | 23.33(17.83-29.71) | 0.88 (0.54 to 1.22) |
| Population < 70 | 0.17(0.14-0.21) | 0.58(0.34-0.96) | 0.67 (0.26 to 1.07) |
| **Georgia** |  |  |  |
| Absolute Numbers | 168(126-202) | 341(293-380) | 2.23 (1.43 to 3.04) |
| Age-Standardized Rates | 3.26(2.37-3.99) | 5.05(4.37-5.62) | 1.43 (0.52 to 2.34) |
| Female | 3.17(2.31-3.83) | 4.08(3.48-4.56) | 0.68 (-0.27 to 1.64) |
| Male | 3.72(2.57-4.80) | 7.22(6.34 -8.07) | 2.62 (1.84 to 3.41) |
| Population 70+ | 42.38(31.05-51.54) | 78.34(66.75-87.84) | 1.90 (0.88 to 2.94) |
| Population < 70 | 0.54(0.43-0.63) | 1.50(1.30-1.70) | 3.01 (2.27 to 3.75) |
| **Kazakhstan** |  |  |  |
| Absolute Numbers | 175(139-224) | 350(305-395) | 1.16 (0.81 to 1.52) |
| Age-Standardized Rates | 2.20(1.75-2.79) | 2.97(2.57-3.34) | 0.49 (0.24 to 0.75) |
| Female | 2.18(1.74-2.79) | 2.91(2.51-3.31) | 0.51 (0.25 to 0.76) |
| Male | 2.24(1.72 -2.88) | 3.08(2.69-3.45) | 0.43 (0.15 to 0.71) |
| Population 70+ | 29.12(23.09-37.28) | 35.06(30.46-39.34) | 0.08 (-0.36 to 0.53) |
| Population < 70 | 0.24(0.21-0.27) | 0.36 (0.31-0.41) | 0.30 (-0.09 to 0.69) |
| **Kyrgyzstan** |  |  |  |
| Absolute Numbers | 46(38-56) | 73(61-84) | 1.49 (1.18 to 1.79) |
| Age-Standardized Rates | 2.01(1.65-2.47) | 2.17(1.82-2.49) | 0.37 (0.12 to 0.63) |
| Female | 2.03(1.63-2.53) | 2.02(1.68-2.33) | 0.05 (-0.14 to 0.25) |
| Male | 2.05(1.76-2.39) | 2.65(2.25-3.03) | 1.00 (0.48 to 1.52) |
| Population 70+ | 26.09(21.28-32.47) | 29.11(24.46-33.35) | 0.68 (0.15 to 1.22) |
| Population < 70 | 0.19(0.16-0.22) | 0.25(0.20-0.30) | 0.31 (-0.11 to 0.74) |
| **Tajikistan** |  |  |  |
| Absolute Numbers | 28(20-41) | 39(31.80-48.42) | 0.80 (0.53 to 1.07) |
| Age-Standardized Rates | 1.26(0.90-1.90) | 1.15(0.92-1.44) | 0.61(-0.96 to -0.26) |
| Female | 1.20 (0.88-1.90) | 1.20 (0.93-1.53) | -0.26(-0.62 to 0.10) |
| Male | 1.36(0.85-2.37) | 1.11 (0.79-1.51) | -1.08(-1.46 to -0.71) |
| Population 70+ | 17.82 (12.40-28.12) | 14.89(11.94-18.68) | -0.83(-1.11 to -0.54) |
| Population < 70 | 0.09(0.07 -0.11) | 0.10(0.08-0.12) | -0.41(-0.77 to -0.04) |
| **Turkey** |  |  |  |
| Absolute Numbers | 836(659-1100) | 2923(2332-3533) | 4.96 (4.46 to 5.47) |
| Age-Standardized Rates | 3.59(2.79-4.75) | 3.88(3.05-4.69) | 0.75 (0.17 to 1.33) |
| Female | 3.92 (2.95-5.36) | 4.61(3.38-5.72) | 1.34 (0.51 to 2.17) |
| Male | 2.93 (2.10-3.93) | 2.80(2.20-3.50) | -0.37 (-0.51 to -0.22) |
| Population 70+ | 47.17(36.80-62.52) | 52.71(41.25-63.85) | 1.25 (0.58 to 1.93) |
| Population < 70 | 0.23(0.18-0.30) | 0.31(0.23-0.39) | 0.48 (0.03 to 0.93) |
| **Turkmenistan** |  |  |  |
| Absolute Numbers | 29(24-34) | 86(68-112) | 3.48 (3.25 to 3.72) |
| Age-Standardized Rates | 2.19(1.83-2.65) | 2.89(2.27-3.76) | 0.50 (0.29 to 0.72) |
| Female | 2.10(1.75-2.56) | 2.81 (2.12-3.75) | 0.58 (0.35 to 0.81) |
| Male | 2.34 (1.92-2.87) | 3.17(2.63-3.85) | 0.55 (0.37 to 0.73) |
| Population 70+ | 26.47(21.74-32.67) | 42.21(32.45-56.82) | 1.23 (0.89 to 1.58) |
| Population < 70 | 0.17(0.16-0.20) | 0.40(0.29-0.53) | 2.28 (2.01 to 2.54) |
| **Uzbekistan** |  |  |  |
| Absolute Numbers | 97(57-158) | 240(204-278) | 3.03 (2.66 to 3.41) |
| Age-Standardized Rates | 1.03 (0.60-1.69) | 1.39 (1.18-1.60) | 1.26 (1.00 to 1.52) |
| Female | 0.96(0.58 -1.60) | 1.26 (1.05-1.47) | 1.04 (0.82 to 1.25) |
| Male | 1.15 (0.63-1.87) | 1.72(1.48-1.97) | 1.86 (1.5 to 2.22) |
| Population 70+ | 14.25 (7.81-24.16) | 18.63(15.73- 21.36) | 0.79 (0.31 to 1.28) |
| Population < 70 | 0.08 (0.06-0.10) | 0.19 (0.16-0.22) | 2.74 (2.4 to 3.07) |

Table S10: Atrial fibrillation/flutter in EU-53: DALYs by Country, sex, and age

|  | **1990**  **n (95% UI)** | **2021**  **n (95% UI)** | **1990-2021EAPC**  **(95% CI)** |
| --- | --- | --- | --- |
| **WESTERN EUROPE** | | | |
| **Andorra** |  |  |  |
| Absolute Numbers | 64(49-85) | 170 (130-217) | 2.87 (2.57 to 3.16) |
| Age-Standardized Rates | 125.54 (95.34-164.86) | 102.38(77.85-131.72) | 0.60 (-0.69 to -0.52) |
| Female | 99.57(72.05-137.65) | 81.7(61.19-108.190 | -0.53 (-0.62 to -0.44) |
| Male | 150.30(113.62 -197.29) | 121.57(91.84-163.33) | -0.65 (-0.76 to -0.54) |
| Population 70+ | 1313.72(986.02-1729.34) | 1339.36(1028.44-1738.45) | 0.34 (0.16 to 0.52) |
| Population < 70 | 42.87(30.50-57.48) | 58.36(40.59-80.54) | 0.95 (0.76 to 1.14) |
| **Austria** |  |  |  |
| Absolute Numbers | 14654(12661-16940) | 37096(30927-43902) | 3.35 (3.17 to 3.54) |
| Age-Standardized Rates | 119.39(103.20-137.72) | 175.92(146.38-208.58) | 1.46 (1.25 to 1.68) |
| Female | 101.10 (88.71-115.34) | 139.14 (117.69-163.47) | 1.21 (0.99 to 1.42) |
| Male | 145.81 (122.57-172.25) | 219.41(181.48-263.20) | 1.54 (1.32 to 1.77) |
| Population 70+ | 1480.50(1288.98-1701.85) | 2450.59(2076.95-2882.30) | 1.89 (1.66 to 2.13) |
| Population < 70 | 48.06(38.38-59.07) | 85.44(66.28-106.23) | 2.31(2.1 to 2.53) |
| **Belgium** |  |  |  |
| Absolute Numbers | 17560(14240-21830) | 27975(22943-33735) | 1.56 (1.47 to 1.66) |
| Age-Standardized Rates | 111.27(90.86-138.05) | 103.49(84.22-126.61) | -0.11 (-0.24 to 0.03) |
| Female | 94.29(78.08-116.50) | 83.77(69.59-101.52) | -0.23 (-0.39 to -0.08) |
| Male | 132.31(105.97-166.88) | 125.87(100.03-156.11) | -0.07 (-0.18 to 0.05) |
| Population 70+ | 1376.47(1134.33-1705.26) | 1379.22(1129.45-1667.34) | 0.35 (0.19 to 0.51) |
| Population < 70 | 48.05(35.99-62.74) | 58.81(43.33-77.03) | 1.27 (1.11 to 1.43) |
| **Cyprus** |  |  |  |
| Absolute Numbers | 1241(932-1550) | 2333(1967-2795) | 2.07 (1.91 to 2.23) |
| Age-Standardized Rates | 217.88(160.27-274.78) | 129.79(108.76-154.10) | -1.71 (-1.91 to -1.5) |
| Female | 204.69(137.01-280.91) | 120.14(99.02-143.26) | -1.95 (-2.18 to -1.71) |
| Male | 234.69(179.98-305.83) | 130.57(104.83-156.60) | -1.64 (-1.83 to -1.44) |
| Population 70+ | 1858.44(1347.39-2364.60) | 1404.36(1164.81-1672.60) | -0.98 (-1.10 to -0.86) |
| Population < 70 | 38.97(30.08-50.23) | 35.29(27.09-46.08) | -0.05 (-0.20 to 0.10) |
| **Denmark** |  |  |  |
| Absolute Numbers | 10814(8775-13295) | 18911(15746-22897) | 1.61 (1.38 to 1.84) |
| Age-Standardized Rates | 125.94(101.96-153.71) | 145.11(120.42-176.86) | 0.31 (0.00 to 0.61) |
| Female | 103.71(87.32-124.10) | 112.67(94.11-135.87) | 0.15 (-0.19 to 0.49) |
| Male | 152.68(120.87-192.14) | 181.17(147.63-221.75) | 0.37 (0.09 to 0.65) |
| Population 70+ | 1454.67(1203.14-1803.84) | 1729(1448-2055.51) | 0.44 (-0.02 to 0.89) |
| Population < 70 | 58.51(44.05-75.82) | 80.98(60.21-106.31) | 1.16 (0.89 to 1.43) |
| **Finland** |  |  |  |
| Absolute Numbers | 13810(11123-16890) | 18394(14929-21967) | 0.75 (0.65 to 0.86) |
| Age-Standardized Rates | 190.50(153.58-231.88) | 127.35(103.37-152.38) | -1.47 (-1.6 to -1.33) |
| Female | 161.10(132.19-194.05) | 98.22(79.65-116.32) | -1.82 (-2 to -1.64) |
| Male | 225.63(177.78-281.09) | 160.90(130.53-195.69) | -1.19 (-1.28 to -1.09) |
| Population 70+ | 2204.64(1811.53-2653.09) | 1573.00(1270.17-1875.95) | -1.28 (-1.47 to -1.09) |
| Population < 70 | 81.87(58.76-106.22) | 79.51(62.59-98.76) | 0.21 (0.00 to 0.42) |
| **France** |  |  |  |
| Absolute Numbers | 113184(92060-139793) | 182910(150493-221951) | 1.62 (1.52 to 1.72) |
| Age-Standardized Rates | 128.27(104.29-157.92) | 107.36(86.51-132.29) | -0.60 (-0.65 to -0.55) |
| Female | 107.35 (88.56-131.02) | 85.89(69.75-105.61) | -0.75 (-0.8 to -0.69) |
| Male | 154.07(123.15-192.96) | 132.12(104.51-164.60) | -0.53 (-0.59 to -0.48) |
| Population 70+ | 1661.67(1375.38-2031.25) | 1515.84(1244.13-1852.41) | -0.09 (-0.23 to 0.06) |
| Population < 70 | 50.84(37.33-68.75) | 57.30(40.83-76.91) | 0.48 (0.34 to 0.62) |
| **Germany** |  |  |  |
| Absolute Numbers | 201327(163031-246392) | 397403(335970-463533) | 2.56 (2.41 to 2.71) |
| Age-Standardized Rates | 152.56(123.99-185.96) | 176.87(148.23-208.48) | 0.72 (0.56 to 0.89) |
| Female | 125.89(103.17-152.30) | 136.34(113.36-158.40) | 0.60(0.47 to 0.74) |
| Male | 191.36(151.73-237.73) | 222.54 (182.96-267.58) | 0.64 (0.44 to 0.84) |
| Population 70+ | 1873.53(1529.36-2303.50) | 2373(1994-2737) | 1.03 (0.91 to 1.16) |
| Population < 70 | 69.39(50.66-92.00) | 105.50(79.06-134.90) | 1.41 (1.00 to 1.81) |
| **Greece** |  |  |  |
| Absolute Numbers | 16059(13186-19814)1 | 30788(25091-37704) | 2.02 (1.92 to 2.11) |
| Age-Standardized Rates | 109.08(90.27-133.03) | 105.10(84.64-130.31) | -0.32 (-0.46 to -0.19) |
| Female | 94.54(78.33-115.03) | 85.14(69.25-105.45) | -0.60 (-0.75 to -0.44) |
| Male | 125.53(103.02-154.47) | 128.77(102.73-160.29) | -0.04 (-0.16 to 0.08) |
| Population 70+ | 1233.82(1029.68-1513.50) | 1483.34(1217.19-1806.76) | 0.41 (0.29 to 0.54) |
| Population < 70 | 46.30(34.49-59.87) | 60.49(44.32-80.82) | 0.28 (0.07 to 0.49) |
| **Iceland** |  |  |  |
| Absolute Numbers | 388(324-467) | 945(793-1109) | 3.1 (3.01 to 3.19) |
| Age-Standardized Rates | 128.16 (106.36-154.70) | 144.46 (120.93-170.09) | 0.53 (0.43 to 0.63) |
| Female | 108(91.83-128) | 120(100-140) | 0.49 (0.39 to 0.6) |
| Male | 150.02(120.29-186.12) | 169.68(139.05-206.36) | 0.53 (0.4 to 0.66) |
| Population 70+ | 1639.00(1366.78-1969.46) | 2019.73(1685.49-2373.61) | 1.12 (0.94 to 1.29) |
| Population < 70 | 37.57(29.14-48.33) | 57.85(43.74-73.48) | 1.39 (1.25 to 1.52) |
| **Ireland** |  |  |  |
| Absolute Numbers | 5569(4632-6722) | 9551(7848-11621) | 1.65 (1.46 to 1.83) |
| Age-Standardized Rates | 138.95(116.12-166.07) | 113.62(93.02-138.37) | -0.75 (-0.89 to -0.6) |
| Female | 114.25(97.18-135.01) | 92.08(75.88-111.33) | -0.78 (-0.95 to -0.61) |
| Male | 168.72(139.39-205.44) | 136.43(108.70-167.83) | -0.82 (-0.98 to -0.66) |
| Population 70+ | 1531.39(1285.56-1859.70) | 1443.34(1178.88-1744.94) | -0.25 (-0.44 to -0.06) |
| Population < 70 | 43.63(33.84-56.45) | 46.31(33.17-61.87) | 0.08 (-0.03 to 0.2) |
| **Israel** |  |  |  |
| Absolute Numbers | 6749(5501-8272) | 19691(15654-24214) | 3.73 (3.55 to 3.92) |
| Age-Standardized Rates | 145.13(119.45-175.94) | 148.81(117.35-184.36) | 0.36 (0.17 to 0.55) |
| Female | 124.79(104.36-148.44) | 125.28(98.07-157.89) | 0.31 (0.09 to 0.52) |
| Male | 168.96(136.01-208.88) | 174.47(136.85-216.42) | 0.36 (0.20 to 0.52) |
| Population 70+ | 1651.99(1363.29-2016.60) | 1779.95(1430.67-2139.70) | 0.67 (0.43 to 0.91) |
| Population < 70 | 37.07 (27.20-49.26) | 56.97(40.98-75.47) | 2.03 (1.75 to 2.31) |
| **Italy** |  |  |  |
| Absolute Numbers | 106933(81969-136680) | 206967(163080-257741) | 2.22 (2.09 to 2.35) |
| Age-Standardized Rates | 120.11(93.10-152.35) | 116.35(91.14-146.40) | -0.08 (-0.19 to 0.03) |
| Female | 113.89(89.79-142.90) | 103.99(81.31-130.42) | -0.18 (-0.26 to -0.1) |
| Male | 123.73(94.64-159.28) | 130.58(101.51-165.21) | 0.13 (-0.03 to 0.29) |
| Population 70+ | 1382.12(1065.36-1766.70) | 1663.43(1324.31-2072.34) | 0.69 (0.47 to 0.90) |
| Population < 70 | 59.26(42.00-81.46) | 64.98(45.39-89.26) | 0.30 (0.22 to 0.38) |
| **Luxembourg** |  |  |  |
| Absolute Numbers | 775(667-905) | 1569(1347-1837) | 2.58 (2.45 to 2.7) |
| Age-Standardized Rates | 143.61(124.34-166.52) | 133.18(114.42-156.51) | -0.02 (-0.1 to 0.07) |
| Female | 124.15(107.81-143.77) | 109.58(94.23-127.23) | -0.16 (-0.27 to -0.04) |
| Male | 168.42(143.10-198.31) | 160.12(133.70-191.73) | 0.07 (-0.01 to 0.15) |
| Population 70+ | 1713.91(1483.96-1981.35) | 1914.53(1649.42-2230.63) | 0.65 (0.54 to 0.77) |
| Population < 70 | 54.60(43.84-68.27) | 51.26(39.44-64.24) | -0.17 (-0.35 to 0.01) |
| **Malta** |  |  |  |
| Absolute Numbers | 487(399-589) | 1180(1005-1388) | 3.14 (2.98 to 3.3) |
| Age-Standardized Rates | 121.54(100.54-145.77) | 107.08 (90.69 -126.34) | 0.16 (-0.3 to -0.03) |
| Female | 111.80(95.06-131.70) | 103.69(87.27-120.51) | -0.12 (-0.25 to 0.02) |
| Male | 131.12(104.57-162.65) | 107.21(88.51-129.53) | -0.28 (-0.41 to -0.14) |
| Population 70+ | 1400.45(1156.21-1701.06) | 1369.89(1170.36-1605.89) | 0.23 (0.08 to 0.38) |
| Population < 70 | 40.33(30.38-52.65) | 52.94 (41.85 -66.03) | 1.61 (1.36 to 1.86) |
| **Monaco** |  |  |  |
| Absolute Numbers | 93 (2.66-117.07) | 125(97-156) | 0.87 (0.74 to 1.01) |
| Age-Standardized Rates | 117.26(90.98-147.88) | 109.17(84.56-138.67) | -0.24 (-0.33 to -0.15) |
| Female | 94.29(72.42-118.46) | 87.23(65.73-110.41) | -0.22 (-0.36 to -0.09) |
| Male | 147.22(109.19-186.54) | 133.82(101.31-171.53) | -0.34 (-0.38 to -0.30) |
| Population 70+ | 1492.80(1169.99-1891.40) | 1443.24(1115.98-1797.89) | 0.05 (-0.14 to 0.24) |
| Population < 70 | 74.60(54.24-101.23) | 71.48(51.64-97.07) | 0.01 (-0.08 to 0.10) |
| **Netherlands** |  |  |  |
| Absolute Numbers | 30426(26007-35157) | 50673(42989-59870) | 1.39 (1.24 to 1.53) |
| Age-Standardized Rates | 148.71(126.96-172.08) | 130.44(110.01-154.87) | -0.69 (-0.81 to -0.58) |
| Female | 133.22(115.85-152.62) | 112.74(92.66-133.81) | -0.82 (-0.94 to -0.69) |
| Male | 166.11(140.02-194.55) | 150.69(125.62-178.65) | -0.60 (-0.70 to -0.49) |
| Population 70+ | 1857.02(1608.42-2121.32) | 1679.89(1410.76-1972.96) | -0.5 (-0.6 to -0.41) |
| Population < 70 | 48.42(37.29-60.85) | 63.81(48.17-81.60) | 0.96 (0.89 to 1.04) |
| **Norway** |  |  |  |
| Absolute Numbers | 10706(8932-12818) | 14671(12145-17639) | 0.87 (0.73 to 1.01) |
| Age-Standardized Rates | 143.49(119.37-171.91) | 128.56(105.70-156.78) | -0.44 (-0.59 to -0.30) |
| Female | 114.61(96.11-136.12) | 100.99(84.00-121.78) | -0.45 (-0.60 to -0.30) |
| Male | 179.82 (148.16-217.86) | 158.29(127.75-196.59) | -0.56 (-0.73 to -0.40) |
| Population 70+ | 1733.69(1451.95-2073.43) | 1712.33(1430.67-2050.47) | 0.05 (-0.23 to 0.34) |
| Population < 70 | 59.49(45.60-75.94) | 59.60(42.71-80.90) | 0.35 (0.13 to 0.57) |
| **Portugal** |  |  |  |
| Absolute Numbers | 15111(11965-19103) | 28100(23004-34125) | 2.09 (2.02 to 2.15) |
| Age-Standardized Rates | 116.12(93.14-144.20) | 112.97(91.19-139.04) | -0.67 (-0.75 to -0.59) |
| Female | 102.45(82.51-126.64) | 83.25(67.42-101.80) | -0.71 (-0.81 to -0.61) |
| Male | 132.19(104.87-165.22) | 96.53(78.19-118.23) | -0.61 (-0.68 to -0.53) |
| Population 70+ | 1261.46(1003.05-1596.62) | 1318.24(1075.17-1602.72) | 0.20 (0.11 to 0.30) |
| Population < 70 | 45.99(33.03-61.74) | 54.00(40.49-70.71) | 0.44 (0.31 to 0.57) |
| **San Marino** |  |  |  |
| Absolute Numbers | 50 (40-62) | 84 (64-111) | 2.08 (1.83 to 2.33) |
| Age-Standardized Rates | 133.98(106.29-165.98) | 92.22(69.55-122.85) | -0.86 (-1.03 to -0.69) |
| Female | 115.00(90.27-141.66) | 77.55(57.47-103.52) | -1.07 (-1.27 to -0.86) |
| Male | 154.47(120.79-194.23) | 107.70(78.44-145.18) | 0.69 (-0.87 to -0.50) |
| Population 70+ | 1673.93(1338.44-2065.70) | 1312.90(991.18-1754.34) | -0.28 (-0.54 to -0.03) |
| Population < 70 | 49.96(35.15-66.18) | 55.49(38.17-78.95) | 0.51 (0.39 to 0.64) |
| **Spain** |  |  |  |
| Absolute Numbers | 71588(57640-88764) | 140604(117419-165622) | 2.35 (2.22 to 2.48) |
| Age-Standardized Rates | 132.00(107.21-161.79) | 119.53(98.17-143.01) | -0.26 (-0.35 to -0.18) |
| Female | 116.67(95.51-143.12) | 97.45(80.80-116.14) | -0.63 (-0.75 to -0.51) |
| Male | 148.49(118.46-185.56) | 144.70(116.95-174.41) | 0.12 (0.03 to 0.21) |
| Population 70+ | 1530.44(1235.34-1894.81) | 1683.61(1415.90-1976.63) | 0.55 (0.47 to 0.63) |
| Population < 70 | 53.31(38.46-70.32) | 68.79(51.12-88.77) | 0.46 (0.35 to 0.57) |
| **Sweden** |  |  |  |
| Absolute Numbers | 22758(17956-28457) | 57180(45730-70747) | 3.05 (2.90 to 3.20) |
| Age-Standardized Rates | 137.78(108.56-173.28) | 222.00(175.69-277.17) | 1.61 (1.46 to 1.77) |
| Female | 119.06(94.77-148.57) | 185.71(146.70-232.88) | 1.49 (1.36 to 1.62) |
| Male | 158.16(124.77-199.63) | 260.43(204.24-324.01) | 1.69 (1.49 to 1.89) |
| Population 70+ | 1671.39(1328.49-2104.14) | 3060.51(2464.73-3772.89) | 2.11 (1.82 to 2.40) |
| Population < 70 | 60.21(43.61-80.61) | 95.35(65.14-131.86) | 1.90 (1.63 to 2.17) |
| **Switzerland** |  |  |  |
| Absolute Numbers | 7852(6377-9611) | 14919(12437-17545) | 2.49 (2.25 to 2.72) |
| Age-Standardized Rates | 70.15(56.77-85.88) | 69.34(57.59-82.94) | 0.37 (0.17 to 0.57) |
| Female | 56.69 (46.33-69.39) | 54.40 (44.74-65.13) | 0.31 (0.11 to 0.51) |
| Male | 88.14 (70.41-109.03) | 86.24 (71.37-104.63) | 0.29 (0.10 to 0.49) |
| Population 70+ | 875.80(714.22-1077.34) | 990.86(827.10-1155.97) | 0.78 (0.63 to 0.93) |
| Population < 70 | 28.26(20.61-37.00) | 33.31(24.80-43.58) | 1.06 (0.80 to 1.33) |
| **United Kingdom** |  |  |  |
| Absolute Numbers | 110739(93505-131324) | 176779(150586-207501) | 1.58 (1.45 to 1.72) |
| Age-Standardized Rates | 117.00(99.11-138.75) | 119.73(101.41-141.22) | 0.08 (-0.03 to 0.19) |
| Female | 97.10(83.82-113.16) | 98.81(84.23-115.58) | 0.07 (-0.05 to 0.19) |
| Male | 142.34(117.74-172.38) | 142.03(117.79-169.83) | -0.02 (-0.13 to 0.08) |
| Population 70+ | 1393.67(1189.39-1642.46) | 1582.93(1343.02-1852.52) | 0.54 (0.48 to 0.61) |
| Population < 70 | 47.02(36.60-60.08) | 54.44(41.75-69.02) | 0.54 (0.44 to 0.64) |
| **CENTRAL EUROPE** | | | |
| **Albania** |  |  |  |
| Absolute Numbers | 1700(1354-2122) | 4399(3431-5468) | 3.46 (3.35 to 3.56 |
| Age-Standardized Rates | 97.60(78.23-120.32) | 103.15(81.18-126.41) | 0.39 (0.29 to 0.48) |
| Female | 84.70(68.07-103.67) | 94.51(71.74-114.28) | 0.66 (0.52 to 0.8) |
| Male | 118.45(89.10-148.22) | 112.57(84.51-144.19) | -0.16 (-0.23 to -0.09) |
| Population 70+ | 1078.44(871.32-1333.57) | 1175.81(906.61-1446.18) | 0.54 (0.41 to 0.67) |
| Population < 70 | 19.44 (14.22-26.25) | 50.51(35.38-71.09) | 3.23 (3.16 to 3.31) |
| **Bosnia and Herzegovina** |  |  |  |
| Absolute Numbers | 3085(2409-3907) | 6410(5131-7828) | 2.57 (2.38 to 2.76) |
| Age-Standardized Rates | 89.03(70.69-112.39) | 99.83(80.18-121.35) | 0.46 (0.40 to 0.52) |
| Female | 74.98(59.76-95.52) | 85.32(67.27-104.08) | 0.54 (0.46 to 0.62) |
| Male | 108.68(83.24-137.51) | 117.90(92.64-145.31) | 0.32 (0.22 to 0.42) |
| Population 70+ | 992.46(783.59-1269.03) | 1173.69(945.34-1425.13) | 0.79 (0.64 to 0.94) |
| Population < 70 | 32.82(24.16-43.67) | 63.46(47.08-83.17) | 1.64 (1.42 to 1.86) |
| **Bulgaria** |  |  |  |
| Absolute Numbers | 12305(10093-15114) | 19898(16801-23531) | 1.56 (1.47 to 1.66) |
| Age-Standardized Rates | 118.85(99.82-142.79) | 132.45(112.78-156.09) | 0.39 (0.29 to 0.48) |
| Female | 103.99(86.46-124.04) | 102.60(86.50-121.80) | -0.06 (-0.18 to 0.05) |
| Male | 135.79(104.90-171.95) | 174.18(147.90-205.31) | 0.89 (0.80 to 0.98) |
| Population 70+ | 1099.21(910.85-1353.31) | 1427.88(1209.08-1678.08) | 1.00 (0.89 to 1.11) |
| Population < 70 | 63.03(49.22-80.27) | 90.30(71.95-111.76) | 1.27 (1.11 to 1.43) |
| **Croatia** |  |  |  |
| Absolute Numbers | 3763(3147-4496) | 7519(6521-8661) | 2.72 (2.44 to 2.99) |
| Age-Standardized Rates | 69.49(58.62-82.02) | 76.37(65.94-88.41) | 0.63 (0.34 to 0.91) |
| Female | 57.94 (49.04-69.16) | 60.72(53.13-68.22) | 0.43 (0.05 to 0.8) |
| Male | 89.06(75.05-105.91) | 97.31(81.70-116.16) | 0.65 (0.48 to 0.81) |
| Population 70+ | 829.06(720.61-962.83) | 968.72(842.16-1098.61) | 1.20 (0.64 to 1.76) |
| Population < 70 | 30.86 (23.67-40.35) | 43.49(33.68-54.81) | 1.29 (1.14 to 1.45) |
| **Czech Republic** |  |  |  |
| Absolute Numbers | 14372(11698-17635) | 31633(25775-37952) | 2.91 (2.75 to 3.07) |
| Age-Standardized Rates | 105.91(86.52-128.90) | 135.11(109.98-161.89) | 0.99 (0.76 to 1.22) |
| Female | 89.70(74.15-109.53) | 108.01(88.20-129.26) | 0.74 (0.56 to 0.92) |
| Male | 130.77(106.23-160.02) | 169.62(136.19-205.44) | 1.10 (0.81 to 1.40) |
| Population 70+ | 1208.73(1002.30-1488.82) | 1602.61(1304.19-1901.84) | 1.31 (1.09 to 1.53) |
| Population < 70 | 49.89(37.18-65.01) | 75.73(57.69-95.85) | 1.99 (1.75 to 2.23) |
| **Hungary** |  |  |  |
| Absolute Numbers | 15494(12523-19139) | 19640(15966-23931) | 0.97 (0.91 to 1.03) |
| Age-Standardized Rates | 109.79(90.18-134.09) | 92.84(75.06-114.33) | -0.44 (-0.5 to -0.37) |
| Female | 95.15(79.15-114.91) | 79.99(64.90-97.82) | -0.46 (-0.52 to -0.41) |
| Male | 131.37(105.81-161.81) | 110.61(88.47-137.62) | -0.43 (-0.51 to -0.35) |
| Population 70+ | 1211.18(1005.63-1486.75) | 1074.92(884.70-1318.33) | -0.08 (-0.16 to 0.01) |
| Population < 70 | 54.74(41.15-71.79) | 62.58(47.19-81.40) | 0.66 (0.57 to 0.75) |
| **Montenegro** |  |  |  |
| Absolute Numbers | 1184(1023-1423) | 2331(1944-2768) | 2.53 (2.42 to 2.64) |
| Age-Standardized Rates | 204.40(176.69-246.84) | 266.09(221.18-320.05) | 1.04 (0.81 to 1.28) |
| Female | 148.47(124.93-179.02) | 208.02(164.07-254.39) | 1.36 (1.15 to 1.57) |
| Male | 286.45(236.66-357.96) | 350.34(284.06-441.23) | 0.66 (0.37 to 0.95) |
| Population 70+ | 2442.17(2079.14-2993.04) | 2952.90(2421.39-3600.35) | 0.93 (0.57 to 1.29) |
| Population < 70 | 67.64(56.25-80.70) | 109.50(86.12-132.48) | 1.46 (1.30 to 1.62) |
| **North Macedonia** |  |  |  |
| Absolute Numbers | 1732(1418-2143) | 3664(2801-4933) | 2.56 (2.47 to 2.66) |
| Age-Standardized Rates | 106.33(88.49-129.52) | 136.26(107.56-174.16) | 0.72 (0.41 to 1.03) |
| Female | 89.88(72.46-111.47) | 128.78(96.75-181.01) | 0.98 (0.67 to 1.29) |
| Male | 127.32(101.77-158.55) | 140.74(111.05-173.70) | 0.24 (0.13 to 0.35) |
| Population 70+ | 1257.48(1042.93-1533.12) | 1330.74(1029.65-1791.58) | 0.24 (0.13 to 0.35) |
| Population < 70 | 32.75(24.10-42.92) | 57.15(40.62-78.74) | 1.63 (1.56 to 1.71) |
| **Poland** |  |  |  |
| Absolute Numbers | 57914(49713-67500) | 99702(80721-122377) | 1.82 (1.5 to 2.14) |
| Age-Standardized Rates | 141.14(122.41-163.83) | 130.17(104.84-160.59) | -0.34 (-0.7 to 0.02) |
| Female | 130.39(111.58-152.14) | 113.90(92.39-139.64) | 0.48 (-0.84 to -0.11) |
| Male | 156.35(133.46-185.92) | 151.48(119.74-189.74) | 0.44 (0.12 to 0.77) |
| Population 70+ | 1610(1400-1868) | 1551(1268-1884) | 0.10 (-0.23 to 0.42) |
| Population < 70 | 53.61(42.81-66.57) | 81.18(59.73-108.11) | 1.26 (1.02 to 1.50) |
| **Romania** |  |  |  |
| Absolute Numbers | 22242(17716-28195) | 31380(26640-36872) | 0.95 (0.76 to 1.14) |
| Age-Standardized Rates | 90.76(73.88-112.51) | 77.45(65.23-91.39) | -0.79 (-0.95 to -0.62) |
| Female | 80.45(66.18-97.06) | 66.16(56.51-76.95) | -0.9 (-1.07 to -0.73) |
| Male | 103.39(81.52-131.50) | 92.54(76.93-110.96) | -0.64 (-0.81 to -0.48) |
| Population 70+ | 964.09(783.48-1200.28) | 936.70(807.21-1093.92) | -0.21 (-0.51 to 0.08) |
| Population < 70 | 38.65(28.54-51.41) | 46.74(37.59-58.76) | 0.34 (0.02 to 0.66) |
| **Serbia** |  |  |  |
| Absolute Numbers | 10432(8568-12759) | 16205(13503-19228) | 1.56 (1.41 to 1.72) |
| Age-Standardized Rates | 120.07(99.86- 144.10) | 92.99(77.52-110.36) | -1.12 (-1.35 to -0.89) |
| Female | 102.23(84.45-122.37) | 78.99(64.05-94.63) | -1.34 (-1.6 to -1.07) |
| Male | 145.69(116.88-176.50) | 111.64(90.63-134.69) | -0.95 (-1.16 to -0.73) |
| Population 70+ | 1319.98(1098.06-1565.22) | 1160.93(970.26-1372.24) | -0.03 (-0.35 to 0.29) |
| Population < 70 | 42.44(32.21-55.69) | 46.61(35.73-58.49) | 0.17 (-0.05 to 0.40) |
| **Slovakia** |  |  |  |
| Absolute Numbers | 8402(6989-10006) | 14612(11797-17705) | 1.85 (1.79 to 1.90) |
| Age-Standardized Rates | 144.92(121.43-171.50) | 150.56(121.36-182.31) | 0.18 (0.08 to 0.28) |
| Female | 130.03(108.60-153.93) | 135.88(109.49-164.70) | 0.15 (0.06 to 0.23) |
| Male | 163.66(132.76-199.30) | 167.28(131.80-206.24) | 0.20 (0.07 to 0.34) |
| Population 70+ | 1620.30(1373.51-1927.94) | 1727.43(1418.13-2055.91) | 0.48 (0.36 to 0.59) |
| Population < 70 | 61.50(47.50-77.74) | 87.55(66.34-111.13) | 1.43 (1.31 to 1.55) |
| **Slovenia** |  |  |  |
| Absolute Numbers | 2338(1905-2882) | 4654(3899-5468) | 2.53 (2.35 to 2.70) |
| Age-Standardized Rates | 95.87(78.17-117.88) | 92.57(76.97-109.58) | 0.02 (-0.2 to 0.25) |
| Female | 84.24(69.66-103.89) | 118.98(97.98-143.06) | -0.28 (-0.5 to -0.07) |
| Male | 112.40(89.49-139.84) | 72.69(60.36-85.81) | 0.29 (0.04 to 0.54) |
| Population 70+ | 1145.41(950.50-1409.79) | 1207.89(1016.99-1397.01) | 0.49 (0.36 to 0.63) |
| Population < 70 | 41.88(31.63-55.48) | 57.90(43.64-74.15) | 0.92 (0.8 to 1.04) |
| **EASTERN EUROPE** | | | |
| **Belarus** |  |  |  |
| Absolute Numbers | 11328(9217-14087) | 16740(13493-20892) | 1.20 (1.14 to 1.25) |
| Age-Standardized Rates | 91.19(74.49-113.12) | 101.79(82.13-126.55) | 0.24 (0.17 to 0.31) |
| Female | 83.63(69.72-100.61) | 89.37(72.43-110.79) | 0.05(-0.02to 0.12) |
| Male | 102.41(74.90-135.38) | 122.92(98.66-152.70) | 0.58 (0.52 to 0.63) |
| Population 70+ | 1023.47(843.37-1250.46 | 1158.21(960.92-1425.17) | 0.48 (0.31 to 0.66) |
| Population < 70 | 43.16(31.27-57.74) | 67.96(49.68-89.59) | 1.12 (0.94 to 1.30) |
| **Estonia** |  |  |  |
| Absolute Numbers | 1863(1545-2308) | 3559(3013-4202) | 2.15 (1.99 to 2.31) |
| Age-Standardized Rates | 94.23(78.75-115.45) | 114.15(94.72-136.36) | 0.55 (0.49 to 0.62) |
| Female | 83.48(70.29-102.47) | 93.95(78.43-113.75) | 0.30 (0.25 to 0.34) |
| Male | 114.75(93.26- 141.43) | 150.25(125.59-179.09) | 0.85 (0.75 to 0.95) |
| Population 70+ | 1026.38(873.90-1239.55) | 1479.64(1269.29-1726.70) | 1.14 (0.92 to 1.37) |
| Population < 70 | 44.49(33.00-59.59) | 64.97(47.14-87.18) | 0.99 (0.87 to 1.11) |
| **Latvia** |  |  |  |
| Absolute Numbers | 3115(2573-3792) | 4759(4088-5510) | 1.72 (1.54 to 1.9) |
| Age-Standardized Rates | 88.84(73.85-107.54) | 107.11(90.35-125.03) | 0.84 (0.72 to 0.96) |
| Female | 79.39(66.86-94.53) | 89.14(76.01-104.04) | 0.62 (0.49 to 0.75) |
| Male | 105.22(4.67-133.12) | 137.34(114.53-162.18) | 1.11 (0.99 to 1.23) |
| Population 70+ | 993.63(848.01-1199.63) | 1277.99(1109.31-1468.63) | 1.10 (0.82 to 1.38) |
| Population < 70 | 42.99(31.36-57.87) | 73.02(56.42-91.09) | 1.70 (1.61 to 1.80) |
| **Lithuania** |  |  |  |
| Absolute Numbers | 4073(3334-5057) | 6940(5789-8373) | 1.86 (1.77 to 1.94) |
| Age-Standardized Rates | 91.65(75.55-113.69) | 107.61(88.10-131.18) | 0.55 (0.48 to 0.62) |
| Female | 82.18 (68.72-99.63) | 91.65(75.78-112.39) | 0.39 (0.32 to 0.47) |
| Male | 105.69(84.17-133.01) | 133.94(108.79-163.34) | 0.82 (0.74 to 0.91) |
| Population 70+ | 1045.98(886.77-1274.58) | 1298.51(1103.68-1571.03) | 0.73 (0.54 to 0.91) |
| Population < 70 | 41.48(30.41-55.59) | 74.11(54.74-98.43) | 1.79 (1.70 to 1.89) |
| **Republic of Moldova** |  |  |  |
| Absolute Numbers | 3481(2822-4378) | 6007(4884-7306) | 1.77 (1.69 to 1.85) |
| Age-Standardized Rates | 94.50(78.13-116.90) | 99.18(80.48-120.79) | 0.08 (-0.01 to 0.16) |
| Female | 85.19(72.02-103.26) | 85.65(70.56-105.08) | -0.07 (-0.15 to 0.01) |
| Male | 108.82(86.99- 136.89) | 119.05(95.26-144.85) | 0.18 (0.08 to 0.29) |
| Population 70+ | 923.87(767.56-1127.30) | 1086.51(904.04-1308.28) | 0.64 (0.51 to 0.77) |
| Population < 70 | 36.36 (27.18- 47.74) | 68.43(51.29-90.70) | 2.07 (1.91 to 2.23) |
| **Russian Federation** |  |  |  |
| Absolute Numbers | 156916(126821-199504) | 271806(222263- 328016) | 1.77 (1.7 to 1.83) |
| Age-Standardized Rates | 97.18(79.68-121.88) | 111.42(91.12-134.81) | 0.43 (0.33 to 0.54) |
| Female | 89.25(73.71-111.59) | 102.14(84.94-122.27) | 0.38 (0.28 to 0.48) |
| Male | 112.62(89.24-142.36) | 124.17(99.80-152.17) | 0.38 (0.26 to 0.49) |
| Population 70+ | 995.26(823.69-1231.92) | 1252.07(1051.88-1488.18) | 0.88 (0.70 to 1.06) |
| Population < 70 | 43.03(31.76-58.18) | 70.99(53.02-94.00) | 1.39 (1.25 to 1.54) |
| **Ukraine** |  |  |  |
| Absolute Numbers | 61515(49362-75818) | 78770(61400-97874) | 2.90 (2.73 to 3.06) |
| Age-Standardized Rates | 92.65(75.62-113.39) | 98.56 (76.69-121.96) | 0.04 (-0.07 to 0.14) |
| Female | 99.57(79.00-125.80) | 90.18(67.75-113.92) | -0.08 (-0.19 to 0.03) |
| Male | 87.58(71.13-106.09) | 111.70(87.01-141.51) | 0.24 (0.13 to 0.34) |
| Population 70+ | 983.02(817.21-1193.59) | 1117.47(874.17-1389.42) | 0.41 (0.24 to 0.58) |
| Population < 70 | 45.40(32.53-60.86) | 63.55(45.25-82.41) | 0.79 (0.61 to 0.98) |
| **Armenia** |  |  |  |
| Absolute Numbers | 1451(1074-1905) | 3355(2607-4231) | 3.12 (2.93 to 3.3 |
| Age-Standardized Rates | 60.37(45.72-79.07) | 76.87(59.76-96.05) | 0.98 (0.81 to 1.15) |
| Female | 53.30 (40.67-68.27) | 65.86(51.53 -82.64) | 0.91 (0.74 to 1.08) |
| Male | 69.99 (51.04-93.50) | 91.77 (70.09-117.82) | 1.05 (0.87 to 1.23) |
| Population 70+ | 659.00 (492.88-864.13) | 887.57(698.76-1103.45) | 1.33 (0.93 to 1.72) |
| Population < 70 | 20.10(13.81-28.35) | 43.08 (30.60-58.93) | 2.17 (1.85 to 2.49) |
| **Azerbaijan** |  |  |  |
| Absolute Numbers | 2729(1994-3648) | 5902(4361-7951) | 2.59 (2.5 to 2.68) |
| Age-Standardized Rates | 62.51(46.28-83.71) | 67.92(51.38-90.14) | 0.43 (0.35 to 0.51) |
| Female | 53.02(38.46-72.77) | 58.39(43.82-77.81) | 0.91 (0.74 to 1.08) |
| Male | 80.96(60.63-106.21) | 81.28(60.06-108.96) | 0.09 (0.00to 0.17) |
| Population 70+ | 653.84(469.09-898.92) | 722.73(536.21-960.55) | 0.60 (0.43 to 0.76) |
| Population < 70 | 17.75(12.13-24.37) | 30.96(21.26-43.26) | 1.24 (0.86 to 1.61) |
| **Georgia** |  |  |  |
| Absolute Numbers | 5209(3807-6550) | 7760(6545-9213) | 1.20 (0.80 to 1.60) |
| Age-Standardized Rates | 89.40(64.79-113.19) | 122.39(102.90-145.33) | 1.00 (0.55 to 1.45) |
| Female | 80.31(58.24-100.94) | 97.31(81.78-116.97) | 0.52 (0.05 to 1.00) |
| Male | 105.98(75.50- 136.76) | 164.90(138.79-194.06) | 1.57 (1.16 to 1.99) |
| Population 70+ | 960.04(687.76-1227.00) | 1421.66(1203.83-1710.88) | 1.26 (0.68 to 1.85) |
| Population < 70 | 39.28(27.99-51.01) | 75.98(61.09-94.50) | 1.85 (1.42 to 2.28) |
| **Kazakhstan** |  |  |  |
| Absolute Numbers | 8359(6442-11000) | 12990(10082-16723) | 1.23 (1.05 to 1.41) |
| Age-Standardized Rates | 74.49(57.90-97.30) | 85.35(68.14-108.69) | 0.22 (0.11 to 0.33) |
| Female | 66.63(51.59-87.06) | 75.83(60.98-95.80) | 0.20 (0.09 to 0.31) |
| Male | 87.35(66.36-116.21) | 99.93(77.38-129.49) | 0.19 (0.09 to 0.30) |
| Population 70+ | 791.68(598.21-1048.22) | 874.92(699.59-1114.06) | 0.15 (-0.04 to 0.35) |
| Population < 70 | 22.85(16.38-31.35) | 32.38(23.99-44.69) | 0.59 (0.37 to 0.81) |
| **Kyrgyzstan** |  |  |  |
| Absolute Numbers | 1740(1344-2258) | 2816(2187-3622) | 1.42 (1.26 to 1.57) |
| Age-Standardized Rates | 64.88(50.53-83.45) | 69.06(54.42-88.30) | 0.22 (0.13 to 0.31) |
| Female | 59.10(46.39-75.66) | 59.55(47.32-75.92) | 0.02(-0.06 to 0.10) |
| Male | 74.49 (57.63-98.18) | 84.51(66.16-107.40) | 0.45 (0.30 to 0.60) |
| Population 70+ | 691.33(531.58-916.46) | 725.67(572.78-923.67) | 0.41 (0.18 to 0.64) |
| Population < 70 | 17.13(12.57-23.02) | 21.34(15.68-28.50) | 0.33 (-0.04 to 0.70) |
| **Tajikistan** |  |  |  |
| Absolute Numbers | 1358(1009-1881) | 2547(1826-3444) | 1.94 (1.72 to 2.16) |
| Age-Standardized Rates | 55.03(41.21-75.82) | 54.26(39.71-72.32) | -0.11 (-0.22 to 0) |
| Female | 47.80(35.40-65.29) | 47.14(34.48-62.95) | -0.12 (-0.24 to 0.01) |
| Male | 64.63(47.03-87.48) | 61.98(44.22-83.96) | -0.22 (-0.31 to -0.12) |
| Population 70+ | 592.40 (423.68-822.48) | 557.53(404.38-757.52) | -0.13 (-0.26 to -0.01) |
| Population < 70 | 11.43(7.79 -15.84) | 14.44(9.73-20.39) | 0.51 (0.10 to 0.92) |
| **Turkey** |  |  |  |
| Absolute Numbers | 19964(15940-24779) | 55942(46418-65880) | 3.62 (3.32 to 3.92) |
| Age-Standardized Rates | 72.06(57.90-89.75) | 67.92(56.42-80.43) | -0.11 (-0.46 to 0.25) |
| Female | 74.24(58.63-96.16) | 69.86(56.05-84.74) | 0.03 (-0.52 to 0.58) |
| Male | 67.56 (51.16- 87.70) | 63.92(52.02-76.97) | -0.39 (-0.5 to -0.28) |
| Population 70+ | 826.20(659.69-1053.46) | 823.26 (674.12-979.01) | 0.45 (0.02 to 0.88) |
| Population < 70 | 13.53(10.23-17.69) | 17.94 (14.34-22.22) | 0.47 (0.08 to 0.86) |
| **Turkmenistan** |  |  |  |
| Absolute Numbers | 1138(887-1460) | 2975(2299-3738) | 3.12 (3.01 to 3.23) |
| Age-Standardized Rates | 70.46(55.65-90.88) | 86.50(67.67-106.69) | 0.53 (0.44 to 0.61) |
| Female | 62.25(49.27-79.18) | 77.02(60.05-95.10) | 0.56 (0.47 to 0.65) |
| Male | 82.56 (64.14-107.71) | 100.48 (77.76-126.30) | 0.49 (0.41 to 0.57) |
| Population 70+ | 717.96 (552.37-946.60) | 943.04(737.78-1190.09) | 0.82 (0.69 to 0.96) |
| Population < 70 | 14.65 (10.71-19.08) | 29.94(21.65-39.36) | 2.11 (1.87 to 2.35) |
| **Uzbekistan** |  |  |  |
| Absolute Numbers | 5343(3749-7549) | 12966(9657-17427) | 2.90 (2.73 to 3.06) |
| Age-Standardized Rates | 50.19(35.20-71.03) | 58.96(44.73-77.90) | 0.59 (0.52 to 0.65) |
| Female | 42.92(30.18-61.07) | 49.66(38.09-65.67) | 0.53 (0.46 to 0.60) |
| Male | 60.75 (41.38-85.27) | 72.09 (54.31-95.86) | 0.65 (0.58 to 0.73) |
| Population 70+ | 540.40(352.32- 772.31) | 617.96(464.12-824.98) | 0.49 (0.31 to 0.67) |
| Population < 70 | 11.09 (7.57-15.43) | 21.26(14.82-29.41) | 1.90 (1.64 to 2.17) |

Table S11 Percentage contributions of major risk factors to age-standardized death/DALYs rates of atrial fibrillation/flutter in 1990(EU-28 countries). DALYs: Disability-Adjusted Life Years.

| **Country** | **High systolic blood pressure (%)** | **High body-mass index (%)** | **Alcohol use (%)** | **Diet high in sodium (%)** | **Lead exposure (%)** | **Tobacoo (%)** |
| --- | --- | --- | --- | --- | --- | --- |
| **Austria** | | | | | | |
| Deaths | 31.6 | 9.1 | 6.5 | 3.0 | 1.5 | 2.5 |
| DALYs | 31.8 | 9.3 | 8.1 | 3.1 | 1.4 | 4.8 |
| **Belgium** | | | | | | |
| Deaths | 33.0 | 9.5 | 6.6 | 2.5 | 3.2 | 2.5 |
| DALYs | 33.0 | 9.7 | 8.1 | 2.6 | 2.9 | 4.9 |
| **Bulgaria** | | | | | | |
| Deaths | 33.6 | 13.2 | 6.7 | 6.8 | 1.7 | 2.6 |
| DALYs | 34.0 | 13.8 | 8.1 | 6.9 | 1.7 | 4.9 |
| **Croatia** | | | | | | |
| Deaths | 34.2 | 13.6 | 4.8 | 6.3 | 1.7 | 2.6 |
| DALYs | 34.6 | 14.4 | 6.6 | 6.6 | 1.7 | 4.9 |
| **Cyprus** | | | | | | |
| Deaths | 30.9 | 8.6 | 3.7 | 1.4 | 2.6 | 2.1 |
| DALYs | 31.3 | 9.0 | 5.2 | 1.6 | 2.5 | 4.2 |
| **Czech Republic** | | | | | | |
| Deaths | 33.2 | 13.4 | 7.2 | 6.9 | 1.3 | 3 |
| DALYs | 32.9 | 14.6 | 9.0 | 7.1 | 1.3 | 5.4 |
| **Denmark** | | | | | | |
| Deaths | 32.9 | 7.8 | 6.9 | 1.6 | 1.6 | 4.3 |
| DALYs | 32.9 | 8.4 | 8.2 | 1.9 | 1.5 | 6.4 |
| **Estonia** | | | | | | |
| Deaths | 34.8 | 13.2 | 3.8 | 0.7 | 1.1 | 2.1 |
| DALYs | 35.1 | 14.3 | 5.8 | 0.8 | 1.1 | 4.7 |
| **Finland** | | | | | | |
| Deaths | 32.1 | 10.5 | 4.9 | 1.6 | 0.8 | 2.0 |
| DALYs | 32.9 | 10.8 | 6.6 | 1.9 | 0.8 | 4.0 |
| **France** | | | | | | |
| Deaths | 31.4 | 9.4 | 7.1 | 1.5 | 2.0 | 2.4 |
| DALYs | 31.2 | 9.9 | 8.9 | 1.6 | 1.9 | 5.0 |
| **Germany** | | | | | | |
| Deaths | 35.1 | 10.2 | 8.1 | 1.5 | 1.1 | 2.5 |
| DALYs | 33.6 | 10.7 | 10 | 1.9 | 1.1 | 5.0 |
| **Greece** | | | | | | |
| Deaths | 26.7 | 9.9 | 5 | 1.6 | 2.6 | 4.1 |
| DALYs | 27.0 | 11 | 6.4 | 1.8 | 2.4 | 6.9 |
| **Hungry** | | | | | | |
| Deaths | 38.5 | 14.8 | 6.1 | 5.7 | 1.4 | 2.4 |
| DALYs | 38.4 | 16.2 | 6.8 | 6.6 | 1.5 | 4.7 |
| **Ireland** | | | | | | |
| Deaths | 32.6 | 10.3 | 5.4 | 1.1 | 1.9 | 3.0 |
| DALYs | 31.8 | 10.9 | 6.9 | 1.3 | 1.8 | 4.5 |
| **Italy** | | | | | | |
| Deaths | 26.4 | 9.1 | 6.3 | 1.8 | 2.2 | 2.2 |
| DALYs | 27.0 | 9.2 | 7.7 | 2.3 | 2.0 | 4.2 |
| **Lativa** | | | | | | |
| Deaths | 34.9 | 13.8 | 4.7 | 1.4 | 1.1 | 2.3 |
| DALYs | 35.4 | 14.6 | 7.1 | 1.7 | 1.1 | 5.5 |
| **Lithuania** | | | | | | |
| Deaths | 35.1 | 14.5 | 4.7 | 1.7 | 1.1 | 2.4 |
| DALYs | 35.8 | 15.0 | 7.1 | 1.7 | 1.1 | 4.9 |
| **Luxembourg** | | | | | | |
| Deaths | 31.9 | 11.5 | 6.9 | 1.5 | 1.6 | 2.4 |
| DALYs | 32.6 | 11.6 | 8.5 | 1.7 | 1.5 | 4.2 |
| **Malta** | | | | | | |
| Deaths | 31.1 | 9.3 | 3.6 | 1.7 | 3.8 | 1.7 |
| DALYs | 31.1 | 9.8 | 5.0 | 2.2 | 3.8 | 3.4 |
| **Netherlands** | | | | | | |
| Deaths | 31.3 | 8.2 | 6.5 | 1.4 | 1.6 | 3.5 |
| DALYs | 31.5 | 8.5 | 7.7 | 1.6 | 1.5 | 5.2 |
| **Poland** | | | | | | |
| Deaths | 28.2 | 12.4 | 4.4 | 5.1 | 1.9 | 2.6 |
| DALYs | 29.3 | 12.5 | 6.4 | 4.8 | 1.9 | 5.2 |
| **Portugal** | | | | | | |
| Deaths | 27.5 | 9.6 | 6.3 | 1.2 | 3.4 | 1.4 |
| DALYs | 28.4 | 9.8 | 7.7 | 1.6 | 3.2 | 3.2 |
| **Romania** | | | | | | |
| Deaths | 36.3 | 13.2 | 5.4 | 6.1 | 1.7 | 2.4 |
| DALYs | 36.2 | 13.9 | 6.9 | 6.3 | 1.7 | 4.6 |
| **Slovakia** | | | | | | |
| Deaths | 34.2 | 14.8 | 5.4 | 6.3 | 1.4 | 2.4 |
| DALYs | 34.5 | 15.8 | 6.9 | 6.4 | 1.3 | 4.5 |
| **Slovenia** | | | | | | |
| Deaths | 33.8 | 14.8 | 5.4 | 6.4 | 1.3 | 1.6 |
| DALYs | 34.4 | 15.8 | 6.9 | 6.7 | 1.2 | 3.9 |
| **Spain** | | | | | | |
| Deaths | 28,9 | 12.4 | 4.8 | 0.6 | 2.8 | 1.9 |
| DALYs | 29.7 | 13.3 | 6.8 | 1.0 | 2.6 | 4.5 |
| **Sweden** | | | | | | |
| Deaths | 32.1 | 8.0 | 5.3 | 1.7 | 1.0 | 2.5 |
| DALYs | 31.0 | 8.8 | 6.6 | 1.8 | 1.0 | 4.2 |
| **United Kingdom** | | | | | | |
| Deaths | 27.4 | 11.3 | 6.0 | 1.5 | 1.5 | 3.1 |
| DALYs | 27.5 | 12.3 | 7.5 | 1.5 | 1.4 | 3.7 |

Table S12 Percentage contributions of major risk factors to age-standardized death/DALYs rates of atrial fibrillation/flutter in 1990 (other countries in EU-53 except EU-28)

| **Country** | **High systolic blood pressure (%)** | **High body-mass index (%)** | **Alcohol use (%)** | **Diet high in sodium (%)** | **Lead exposure (%)** | **Tobacoo (%)** |
| --- | --- | --- | --- | --- | --- | --- |
| **Albania** | | | | | | |
| Deaths | 34.7 | 8.1 | 0.9 | 7.2 | 1.8 | 4.7 |
| DALYs | 34.8 | 8.3 | 1.4 | 7.8 | 1.9 | 6.7 |
| **Andorra** | | | | | | |
| Deaths | 33.8 | 5.2 | 7.6 | 1.2 | 1.1 | 3.7 |
| DALYs | 33.8 | 5.7 | 9.2 | 1.3 | 1.1 | 6.5 |
| **Armenia** | | | | | | |
| Deaths | 29.6 | 9.2 | 0.8 | 4.6 | 1.7 | 3.9 |
| DALYs | 30.7 | 10.1 | 1.7 | 4.2 | 1.7 | 6.7 |
| **Azerbaijan** | | | | | | |
| Deaths | 29.3 | 7.0 | 1.9 | 4.5 | 1.6 | 3.0 |
| DALYs | 30.0 | 8.3 | 3.1 | 4.1 | 1.6 | 5.2 |
| **Belarus** | | | | | | |
| Deaths | 33.7 | 7.9 | 3.4 | 1.1 | 1.1 | 2.6 |
| DALYs | 34.3 | 8.6 | 5.4 | 1.2 | 1.1 | 5.5 |
| **Bosnia and Herzegovina** | | | | | | |
| Deaths | 36.6 | 6.3 | 2.2 | 7.5 | 2.4 | 4.6 |
| DALYs | 35.9 | 7.5 | 3.0 | 7.9 | 2.5 | 6.5 |
| **Georgia** | | | | | | |
| Deaths | 33.5 | 8.5 | 1.3 | 4.2 | 1.5 | 2.6 |
| DALYs | 35.9 | 9.1 | 2.2 | 3.9 | 1.7 | 4.9 |
| **Iceland** | | | | | | |
| Deaths | 30.7 | 7.3 | 2.6 | 1.8 | 1.7 | 4.2 |
| DALYs | 30.0 | 8.1 | 3.4 | 2.1 | 1.7 | 6.2 |
| **Israel** | | | | | | |
| Deaths | 33.5 | 7.3 | 0.6 | 1.6 | 1.3 | 3.3 |
| DALYs | 33.5 | 7.7 | 0.6 | 1.7 | 1.4 | 4.6 |
| **Kazakhstan** | | | | | | |
| Deaths | 36.7 | 10.9 | 2.6 | 3.9 | 1.1 | 2.0 |
| DALYs | 35.7 | 10.3 | 4.1 | 3.7 | 1.2 | 4.5 |
| **Kyrgyzstan** | | | | | | |
| Deaths | 28.9 | 8 | 0.9 | 4.4 | 1.7 | 2.4 |
| DALYs | 29.3 | 9 | 2.0 | 4.1 | 1.7 | 4.5 |
| **Macedonia** | | | | | | |
| Deaths | 37.2 | 8.3 | 5.9 | 7.4 | 1.5 | 4.4 |
| DALYs | 36.9 | 9.3 | 7.0 | 7.8 | 1.5 | 6.8 |
| **Moldova** | | | | | | |
| Deaths | 32.9 | 9.9 | 4.9 | 1.2 | 1.1 | 1.5 |
| DALYs | 33.4 | 10.6 | 6.9 | 1.3 | 1.2 | 3.7 |
| **Montenegro** | | | | | | |
| Deaths | 37.4 | 11.2 | 5.6 | 7.3 | 1.1 | 4.5 |
| DALYs | 37.2 | 12.1 | 6.3 | 7.7 | 1.2 | 7.0 |
| **Norway** | | | | | | |
| Deaths | 35.9 | 5.1 | 3.0 | 1.3 | 1.3 | 4.7 |
| DALYs | 36.4 | 5.5 | 4.0 | 1.4 | 1.4 | 7.2 |
| **Principality of Monca** | | | | | | |
| Deaths | 33.6 | 8.2 | 3.6 | 1.6 | 1.2 | 3.6 |
| DALYs | 33.6 | 8.8 | 4.7 | 1.7 | 1.3 | 6.2 |
| **Republic of San Marino** | | | | | | |
| Deaths | 33.6 | 7.2 | 7.0 | 1.5 | 1.2 | 3.1 |
| DALYs | 33.8 | 7.7 | 5.6 | 1.7 | 1.3 | 5.5 |
| **Russian Federation** | | | | | | |
| Deaths | 33.7 | 9.0 | 2.1 | 1.9 | 0.9 | 1.8 |
| DALYs | 34.2 | 9.6 | 3.9 | 2.3 | 1.0 | 4.4 |
| Serbia |  |  |  |  |  |  |
| Deaths | 36.8 | 5.9 | 3.7 | 6.6 | 1.3 | 2.8 |
| DALYs | 37.2 | 7.1 | 4.7 | 7.0 | 1.3 | 5.1 |
| **Switzerland** | | | | | | |
| Deaths | 30.0 | 6.1 | 7.9 | 1.6 | 1.7 | 3.9 |
| DALYs | 29.9 | 6.3 | 9.4 | 1.7 | 1.8 | 6.3 |
| **Tajikistan** | | | | | | |
| Deaths | 28.8 | 7.5 | 0.3 | 4.6 | 2.3 | 2.8 |
| DALYs | 29.5 | 8.3 | 1.0 | 4.2 | 2.3 | 4.6 |
| Turkey |  |  |  |  |  |  |
| Deaths | 30.3 | 8.5 | 0.4 | 0.3 | 2.4 | 3.1 |
| DALYs | 30.2 | 9.1 | 0.7 | 0.4 | 2.5 | 5.2 |
| **Turkmenistan** | | | | | | |
| Deaths | 29.8 | 6.4 | 0.7 | 4.5 | 1.5 | 2.3 |
| DALYs | 30.5 | 7.5 | 1.4 | 4.2 | 1.4 | 4.1 |
| **Ukraine** | | | | | | |
| Deaths | 29.8 | 10.5 | 2.3 | 1.1 | 0.9 | 2.2 |
| DALYs | 30.5 | 11.0 | 3.9 | 1.2 | 0.9 | 4.8 |
| **Uzbekistan** | | | | | | |
| Deaths | 25.9 | 7.4 | 0.8 | 4.6 | 1.6 | 1.3 |
| DALYs | 26.3 | 8.1 | 1.6 | 4.2 | 1.5 | 2.6 |


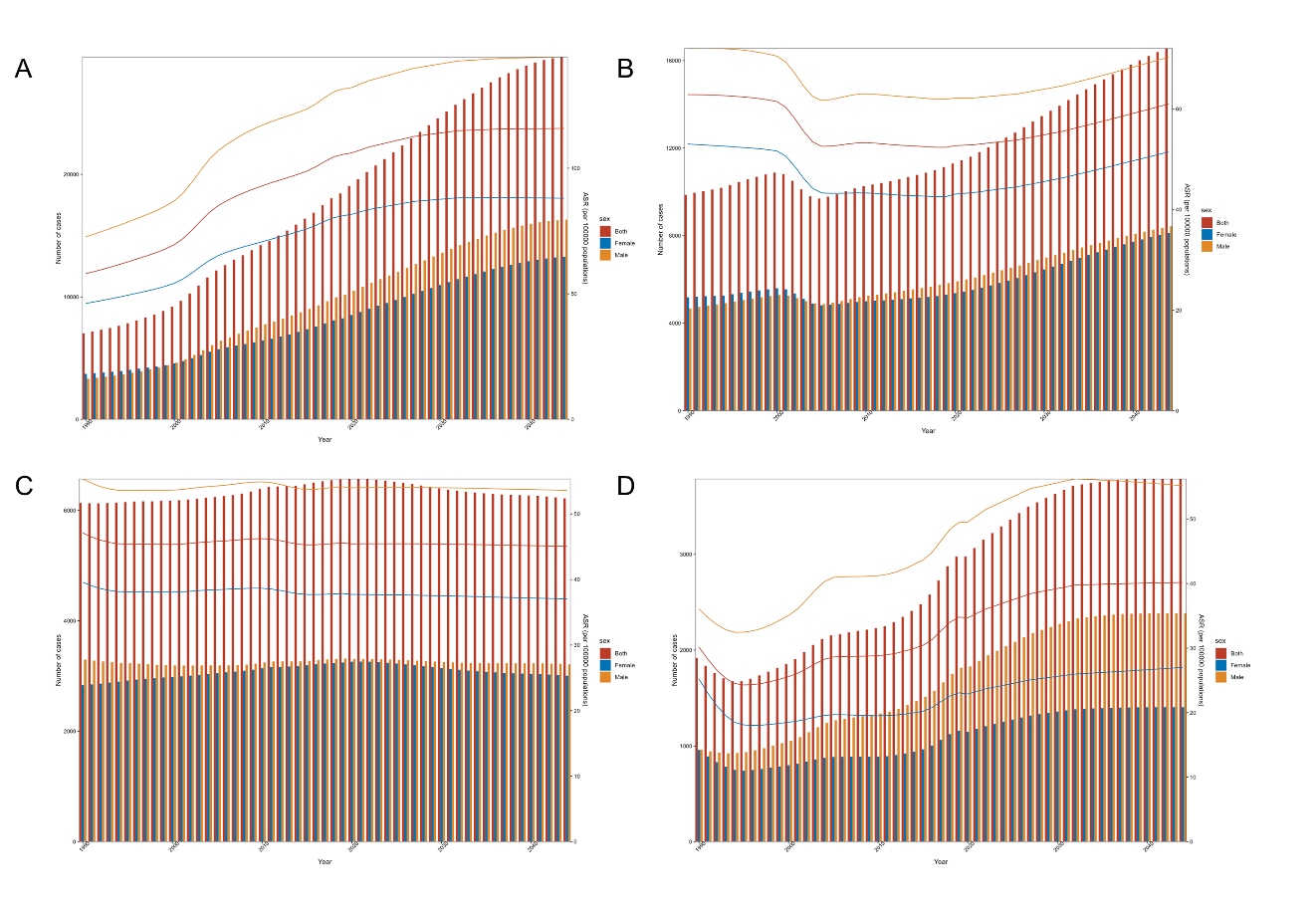


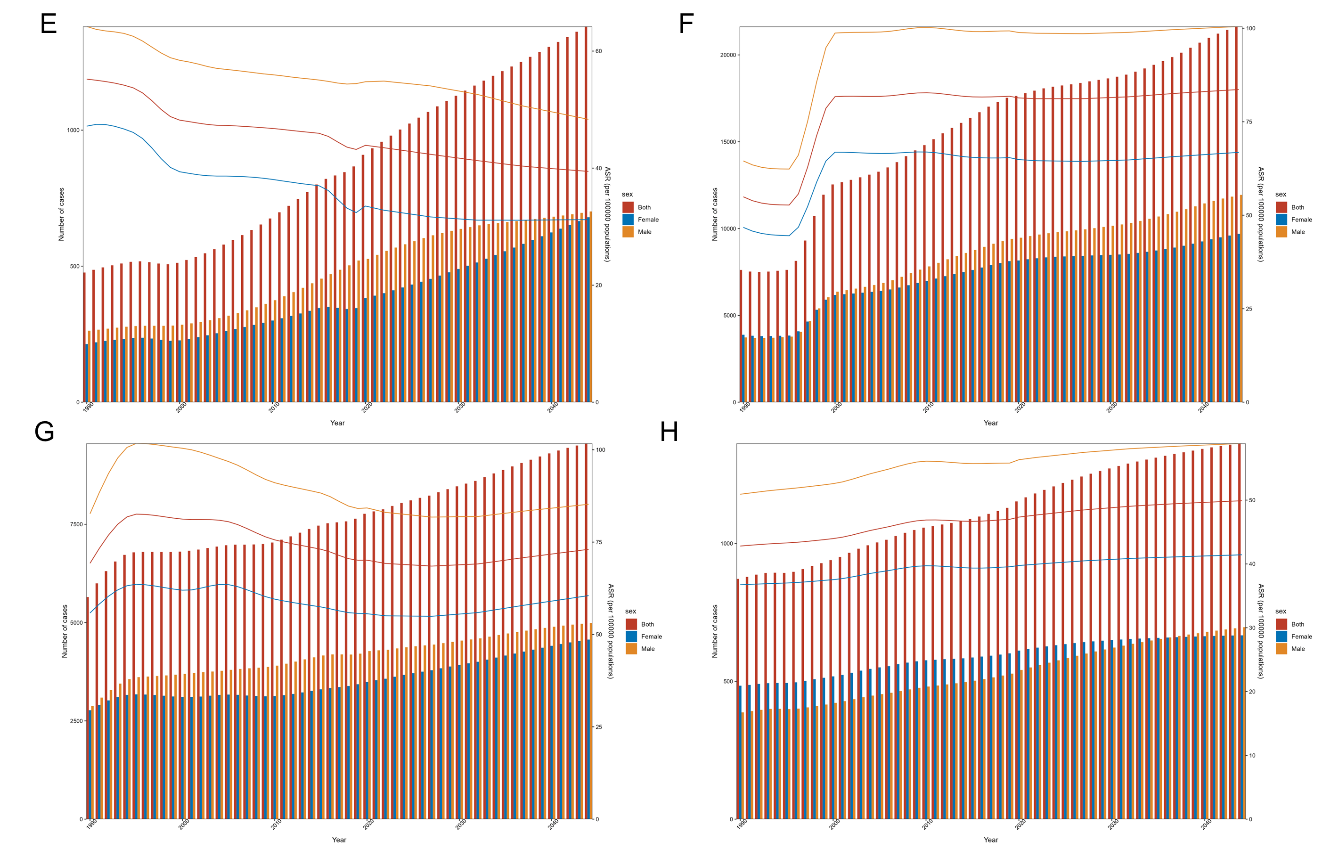


Figure S1 Number of AF/AFL Cases and Age-Standardized Incidence Rates by Sex in the EU-28 (A)Austria, (B)Belgium, (C)Bulgaria, (D)Croatia, (E)Cyprus, (F)Czech Republic, (G)Denmark, (H)Estonia from 1990 to 2044


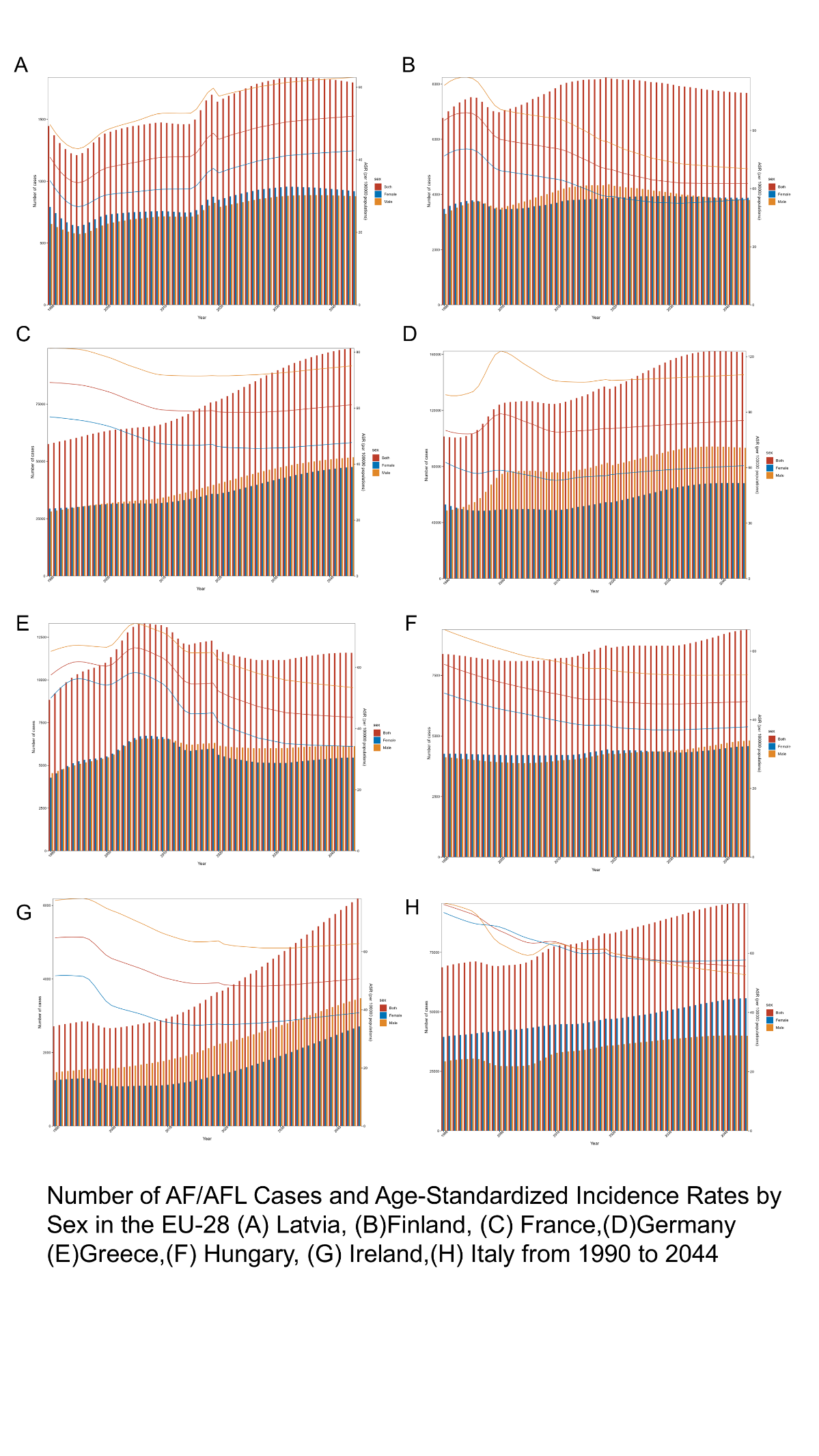


Figure S2 Number of AF/AFL Cases and Age-Standardized Incidence Rates by Sex in the EU-28 (A) Latvia, (B)Finland, (C) France, (D)Germany (E)Greece, (F) Hungary, (G) Ireland, (H) Italy from 1990 to 2044


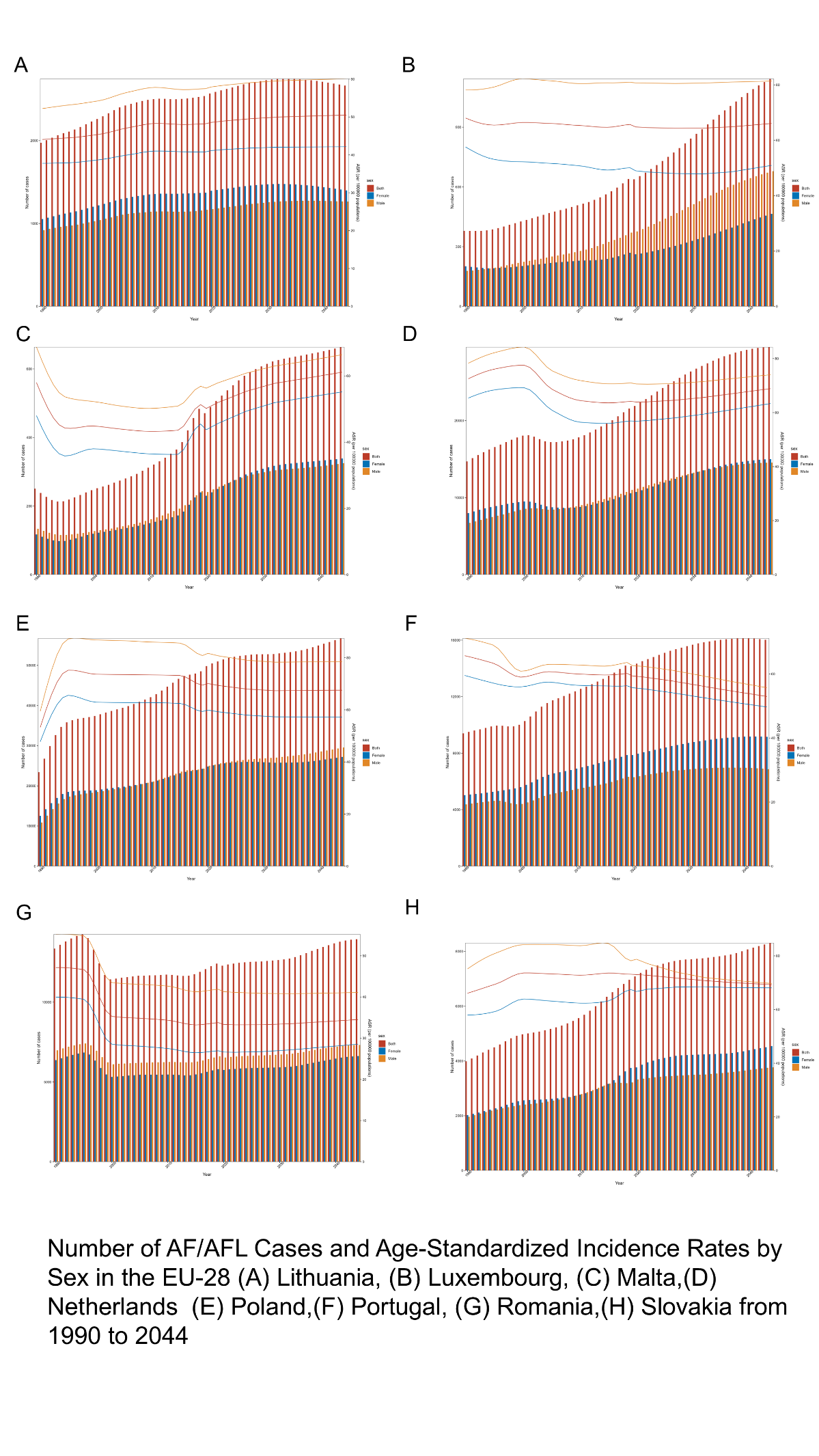


Figure S3 Number of AF/AFL Cases and Age-Standardized Incidence Rates by Sex in the EU-28 (A) Lithuania, (B) Luxembourg, (C) Malta, (D) Netherlands (E) Poland, (F) Portugal, (G) Romania, (H) Slovakia from 1990 to 2044


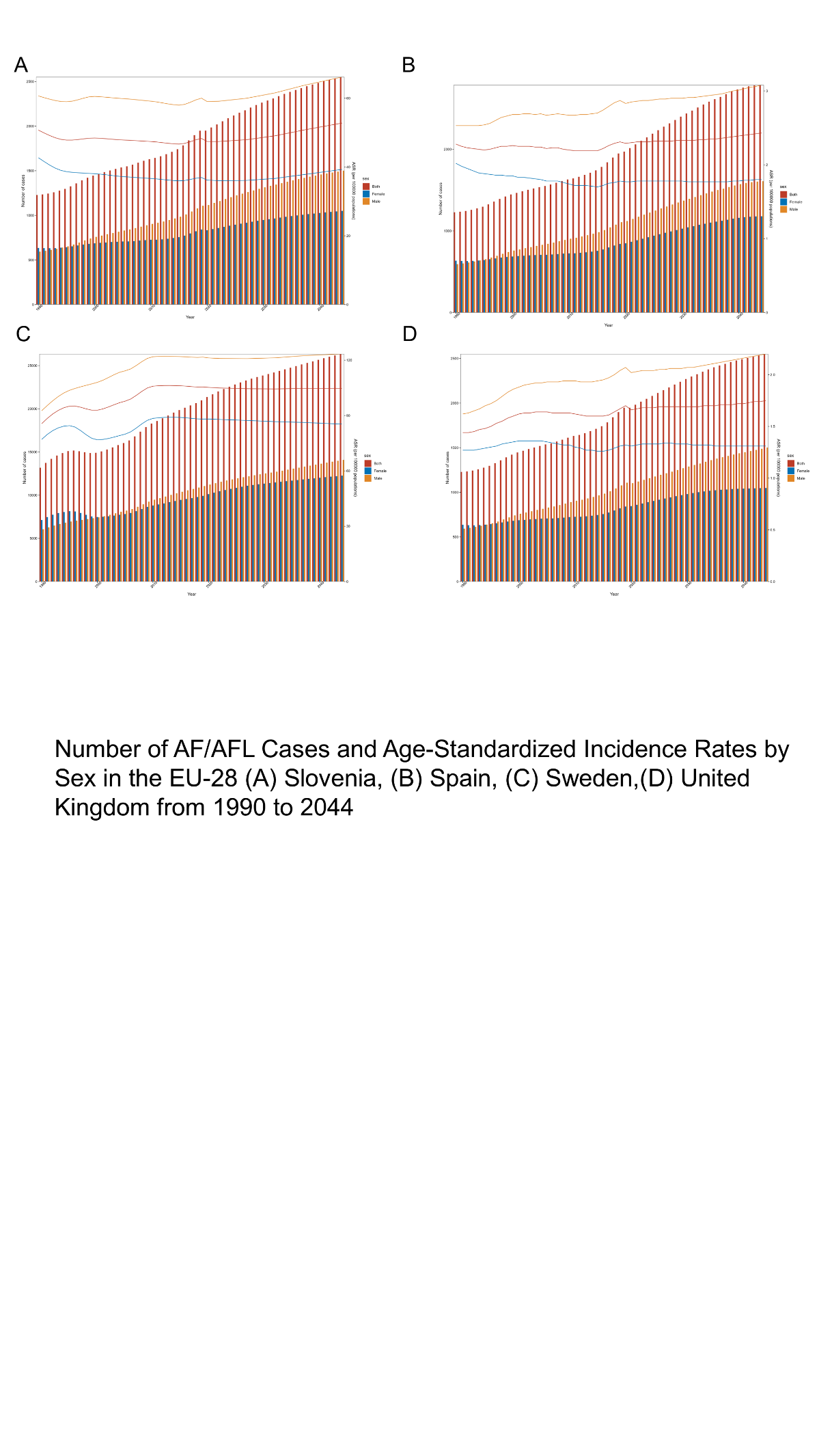


Figure S4 Number of AF/AFL Cases and Age-Standardized Incidence Rates by Sex in the EU-28 (A) Slovenia, (B) Spain, (C) Sweden, (D) United Kingdom from 1990 to 2044


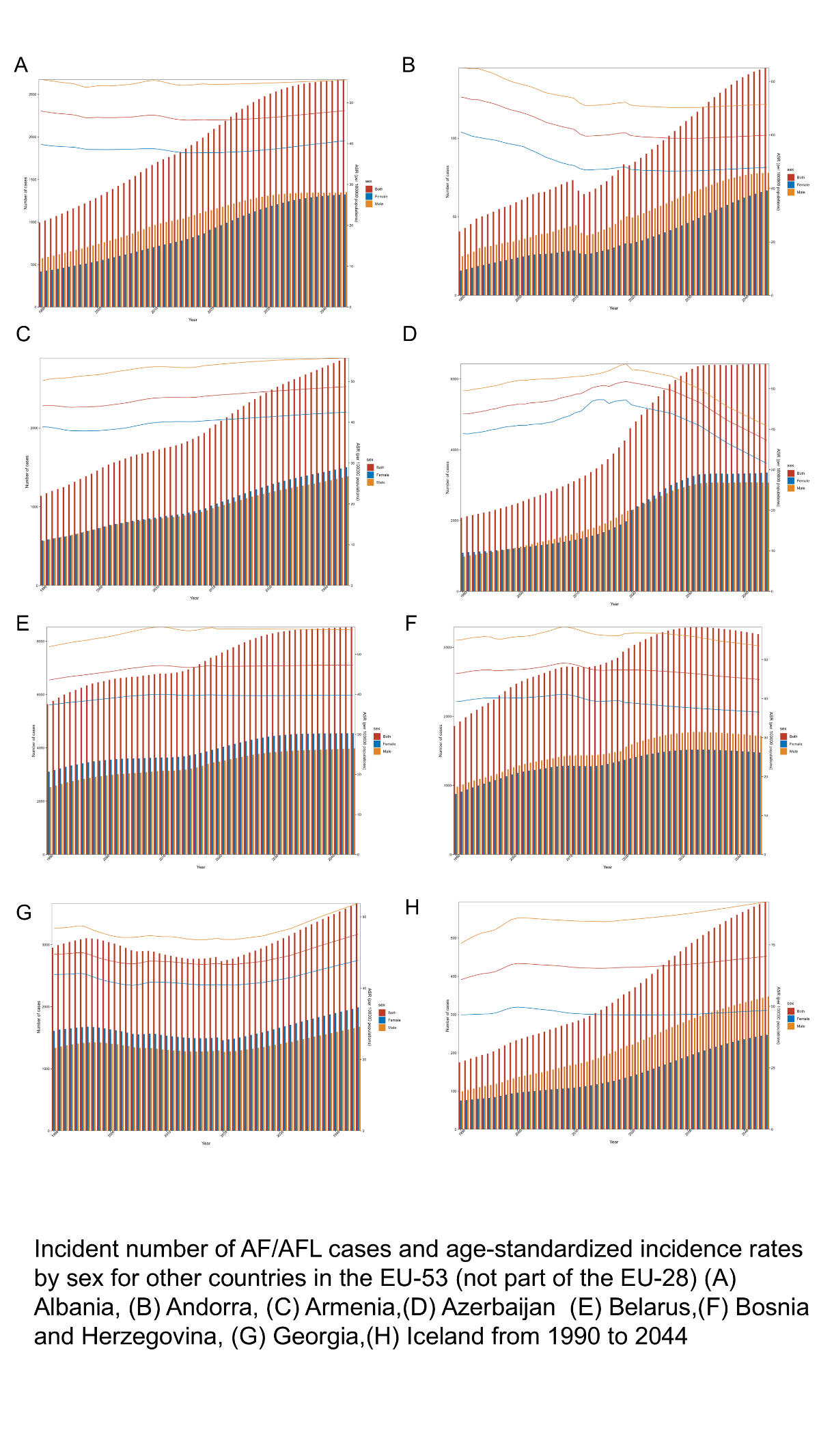


Figure S5 Incident number of AF/AFL cases and age-standardized incidence rates by sex for other countries in the EU-53 (not part of the EU-28) (A) Albania, (B) Andorra, (C) Armenia, (D) Azerbaijan, (E) Belarus, (F) Bosnia and Herzegovina, (G) Georgia, (H) Iceland from 1990 to 2044


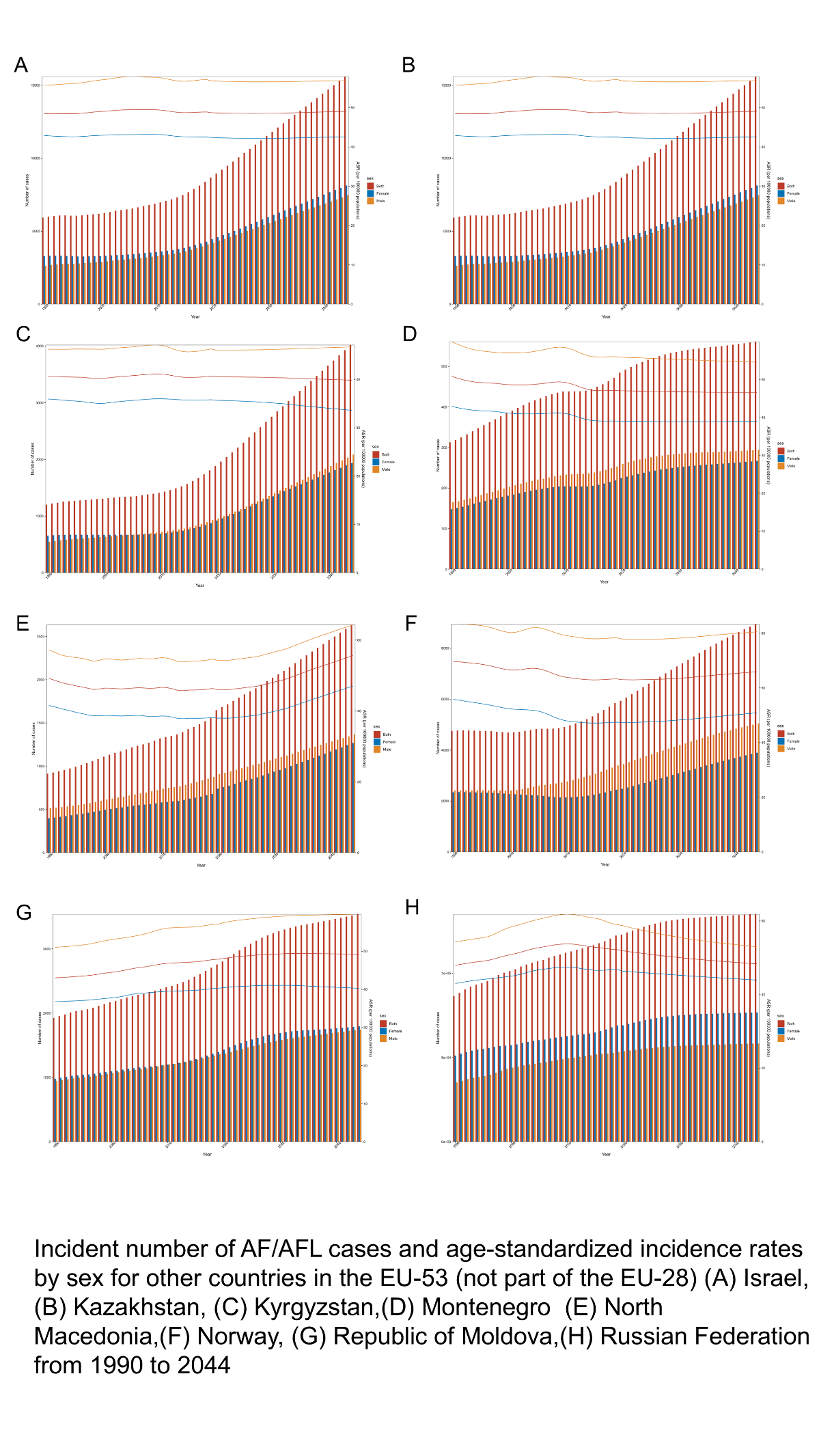


Figure S6 Incident number of AF/AFL cases and age-standardized incidence rates by sex for other countries in the EU-53 (not part of the EU-28) (A) Israel, (B) Kazakhstan, (C) Kyrgyzstan, (D) Montenegro, (E) North Macedonia, (F) Norway, (G) Republic of Moldova, (H) Russian Federation from 1990 to 2044


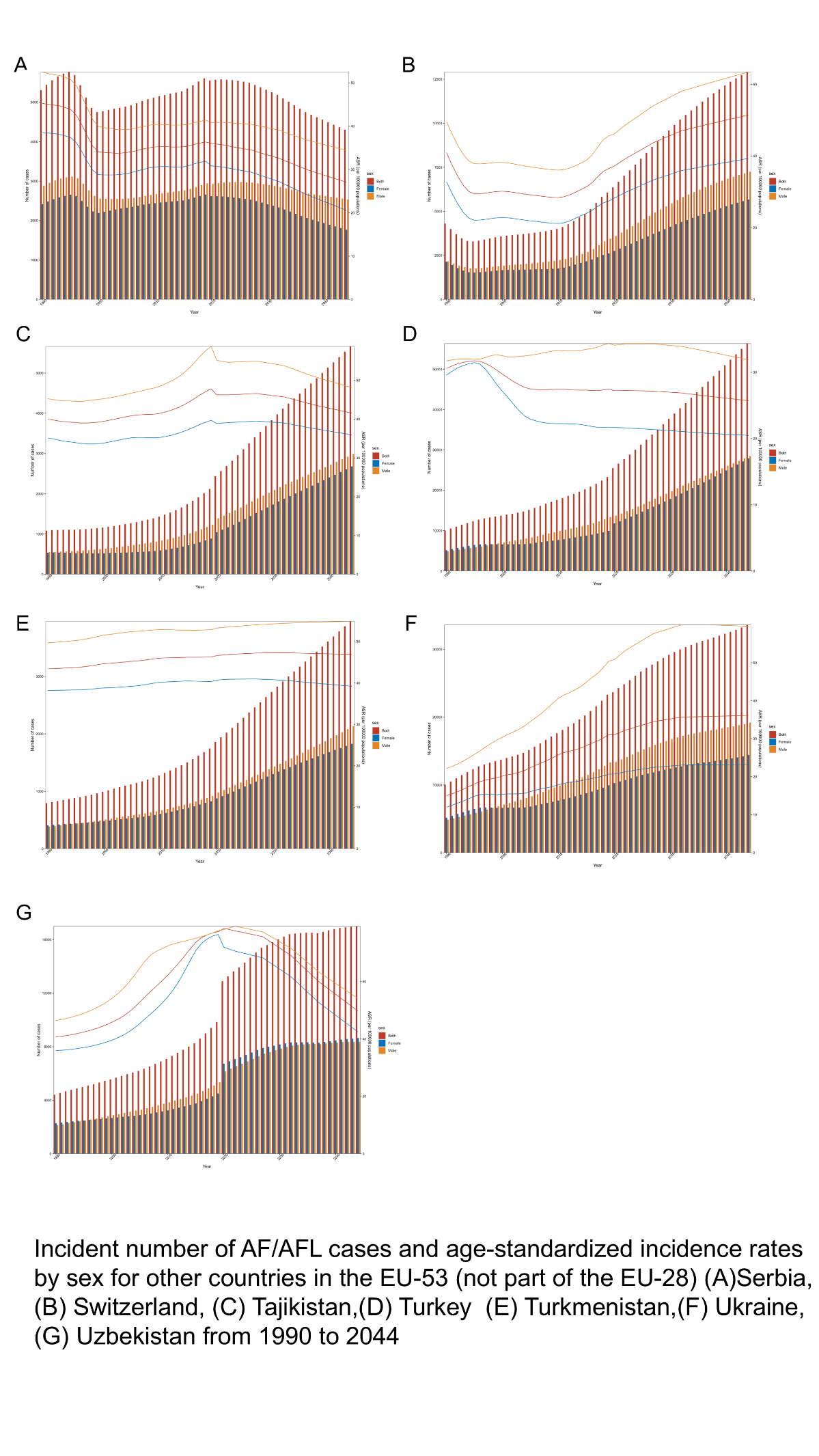


Figure S7 Incident number of AF/AFL cases and age-standardized incidence rates by sex for other countries in the EU-53 (not part of the EU-28) (A)Serbia, (B) Switzerland, (C) Tajikistan, (D) Turkey, (E) Turkmenistan, (F) Ukraine, (G) Uzbekistan from 1990 to 2044
