# Supplementary figures and images for: The burden of atrial fibrillation/atrial flutter in Europe from 1990 to 2021, with a forecast of incidence through 2044
Source: Front Cardiovasc Med. 2025 Jun 18;12:1606024. doi: 10.3389/fcvm.2025.1606024 (PMC12216976; doi:10.3389/fcvm.2025.1606024)

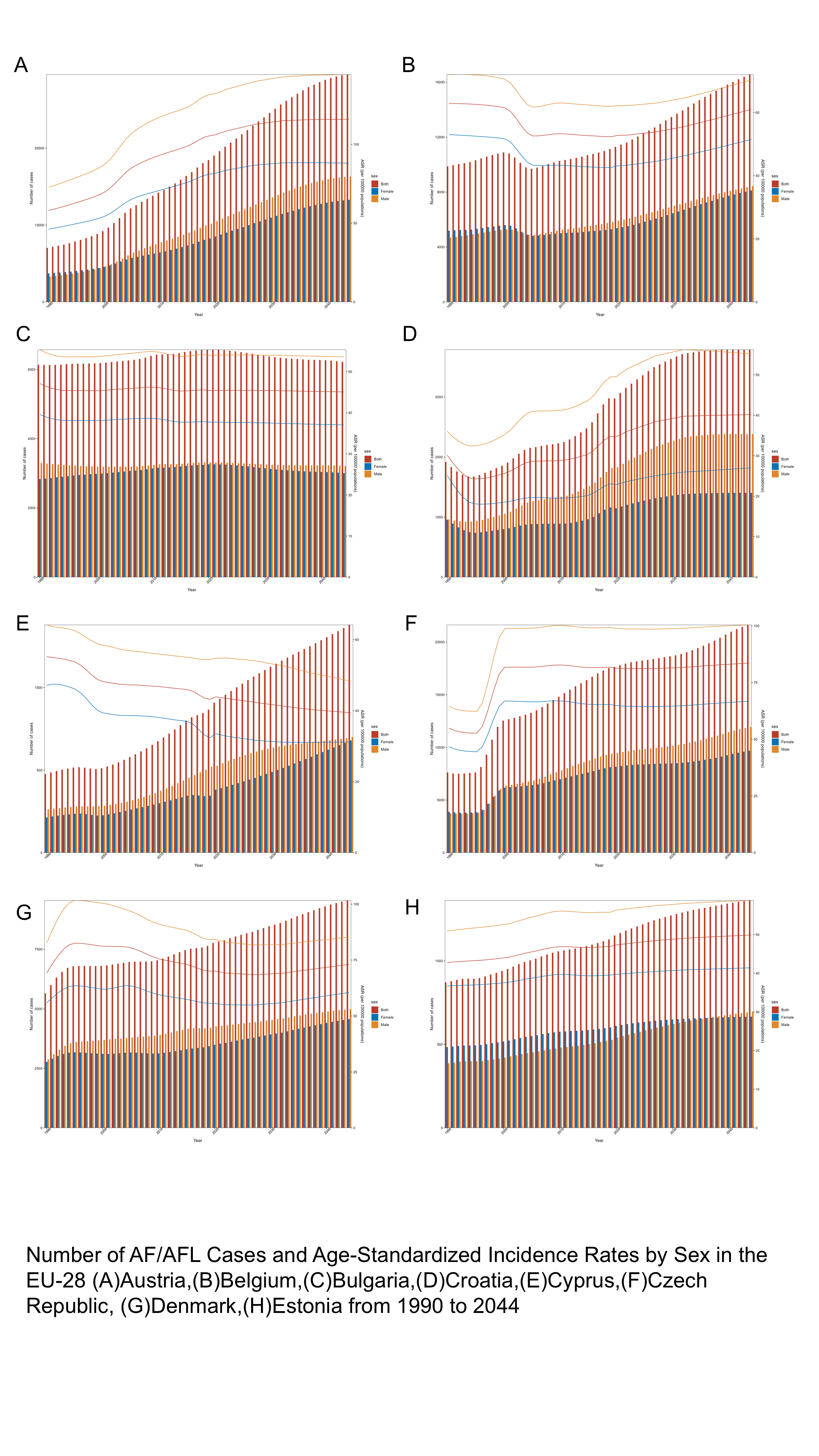

Supplement: Supplementary file 2 [file Image1.tif]

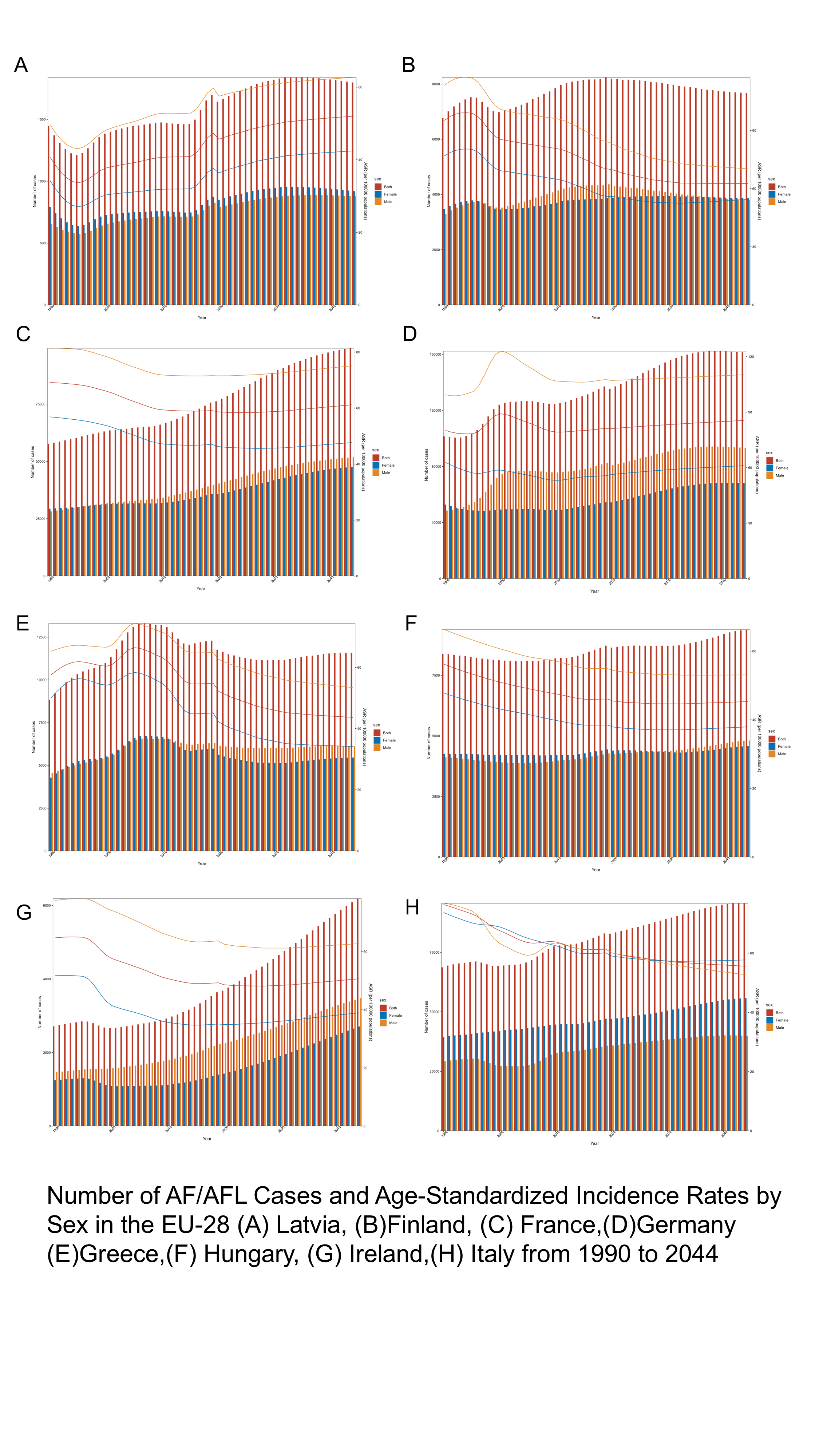

Supplement: Supplementary file 3 [file Image2.tif]

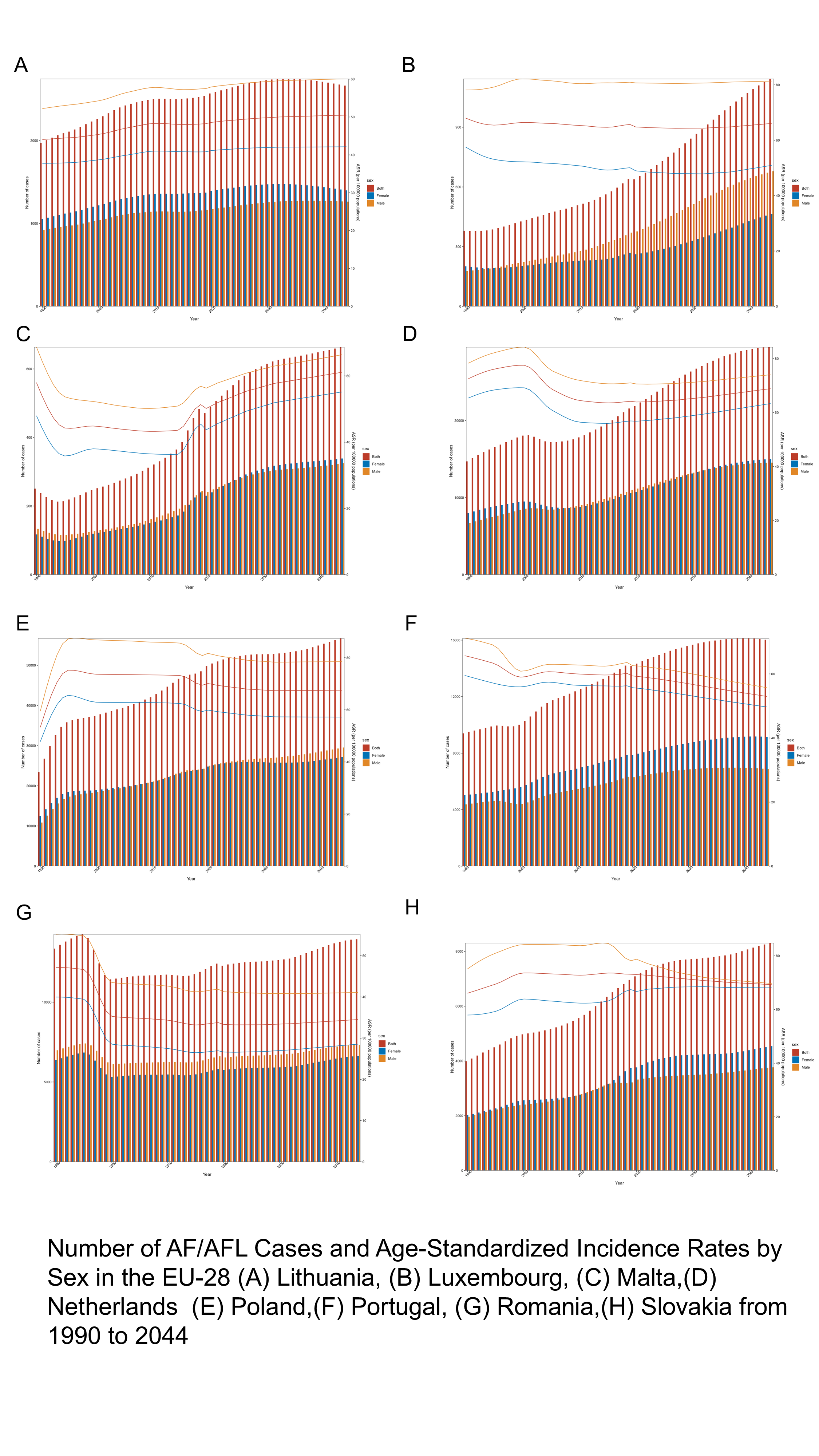

Supplement: Supplementary file 4 [file Image3.tif]

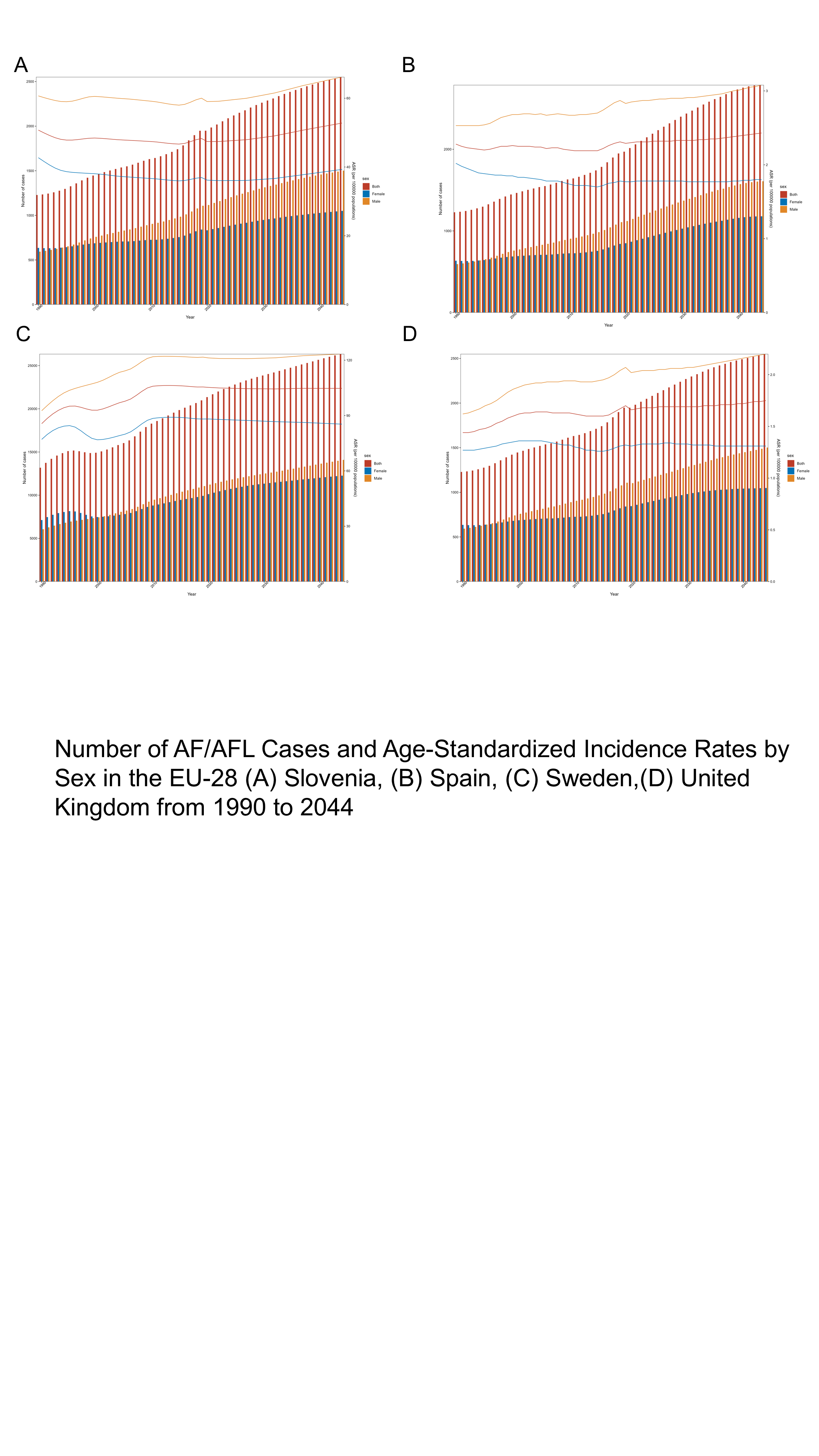

Supplement: Supplementary file 5 [file Image4.tif]

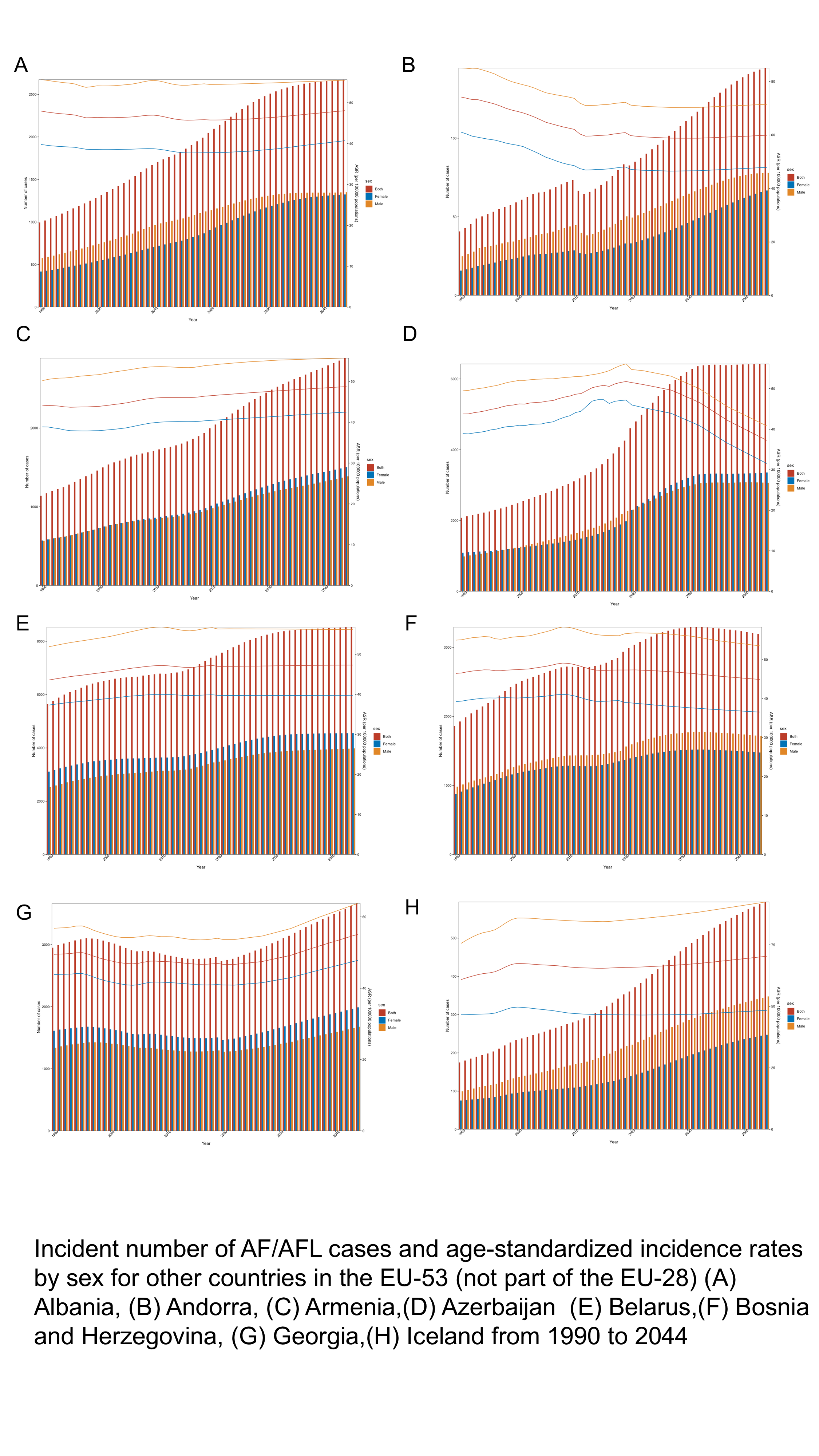

Supplement: Supplementary file 6 [file Image5.tif]

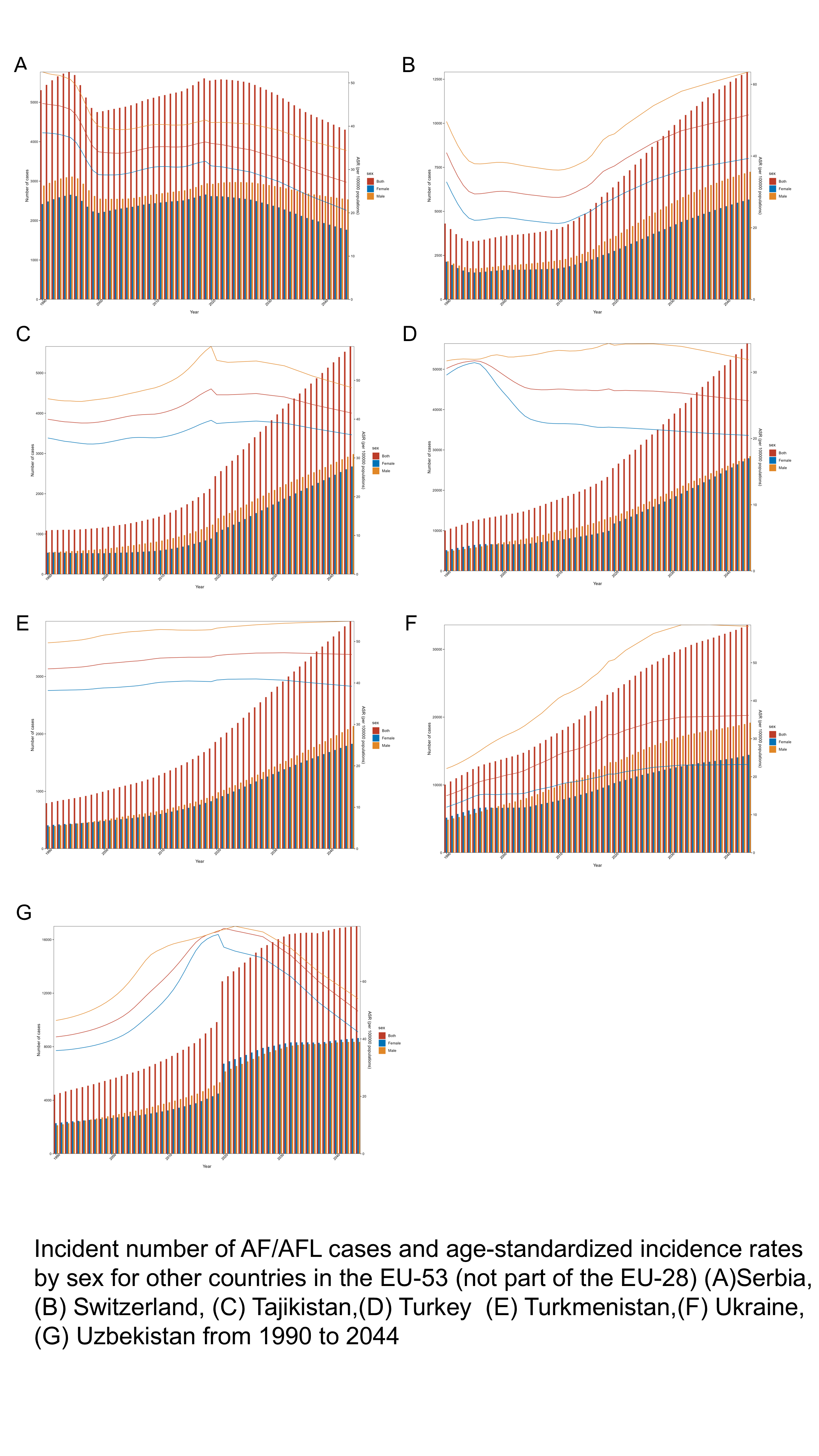

Supplement: Supplementary file 7 [file Image6.tif]
